# Local field potentials in a pre-motor region predict learned vocal sequences

**DOI:** 10.1371/journal.pcbi.1008100

**Published:** 2021-09-23

**Authors:** Daril E. Brown, Jairo I. Chavez, Derek H. Nguyen, Adam Kadwory, Bradley Voytek, Ezequiel M. Arneodo, Timothy Q. Gentner, Vikash Gilja

**Affiliations:** 1 Department of Electrical and Computer Engineering, University of California, San Diego, California, United States of America; 2 Department of Psychology, University of California, San Diego, California, United States of America; 3 Department of Cognitive Science, University of California, San Diego, California, United States of America; 4 Department of Bioengineering, University of California, San Diego, California, United States of America; 5 Kavli Institute for Brain and Mind, University of California, San Diego, California, United States of America; 6 Halıcıoğlu Data Science Institute, University of California, San Diego, California, United States of America; 7 Neurosciences Graduate Program, University of California, San Diego, California, United States of America; 8 IFLP-CONICET, Departamento de Física, Universidad Nacional de La Plata, La Plata, Argentina; 9 Neurobiology Section, Division of Biological Sciences, University of California, San Diego, California, United States of America; University of California at Berkeley, UNITED STATES

## Abstract

Neuronal activity within the premotor region HVC is tightly synchronized to, and crucial for, the articulate production of learned song in birds. Characterizations of this neural activity detail patterns of sequential bursting in small, carefully identified subsets of neurons in the HVC population. The dynamics of HVC are well described by these characterizations, but have not been verified beyond this scale of measurement. There is a rich history of using local field potentials (LFP) to extract information about behavior that extends beyond the contribution of individual cells. These signals have the advantage of being stable over longer periods of time, and they have been used to study and decode human speech and other complex motor behaviors. Here we characterize LFP signals presumptively from the HVC of freely behaving male zebra finches during song production to determine if population activity may yield similar insights into the mechanisms underlying complex motor-vocal behavior. Following an initial observation that structured changes in the LFP were distinct to all vocalizations during song, we show that it is possible to extract time-varying features from multiple frequency bands to decode the identity of specific vocalization elements (syllables) and to predict their temporal onsets within the motif. This demonstrates the utility of LFP for studying vocal behavior in songbirds. Surprisingly, the time frequency structure of HVC LFP is qualitatively similar to well-established oscillations found in both human and non-human mammalian motor areas. This physiological similarity, despite distinct anatomical structures, may give insight into common computational principles for learning and/or generating complex motor-vocal behaviors.

## Author summary

Vocalizations, such as speech and song, are a motor process that requires the coordination of numerous muscle groups receiving instructions from specific brain regions. In songbirds, HVC is a premotor brain region required for singing; it is populated by a set of neurons that fire sparsely during song. How HVC enables song generation is not well understood. Here we describe network activity presumptively from HVC that precedes the initiation of each vocal element during singing. This network activity can be used to predict both the identity of each vocal element (syllable) and when it will occur during song. In addition, this network activity is similar to activity that has been documented in human, non-human primate, and mammalian premotor regions tied to muscle movements. These similarities add to a growing body of literature that finds parallels between songbirds and humans in respect to the motor control of vocal organs. Furthermore, given the similarities of the songbird and human motor-vocal systems, these results suggest that the songbird model could be leveraged to accelerate the development of clinically translatable speech prosthesis.

## Introduction

Learned vocalizations, such as speech and song, are generated by the complex coordination of multiple muscle groups that control the vocal organs [1–3]. As with other voluntary movements, this coordinated action arises from premotor brain regions [4–8] and is prepared prior to the physical initiation of movement [4,9,10]. How these behaviors are encoded during both preparation and generation remains an important topic of ongoing research. Developing an understanding for how the brain encodes complex sequential motor movements carries implications for the development of neural prostheses that aim to return or supplement lost motor function. In addition to their clinical application, such systems will help create new tools for examining the brain’s mechanisms for learning and executing motor sequences.

At present, studying the motor encoding of speech and other complex motor movements in humans is challenging due to the intrinsic hurdles of conducting invasive human studies [11–15] and the complexity of human language [2,14]. Invasive studies that employ implanted electrodes are typically conducted in a clinical setting and clinical studies are inherently difficult. The clinical setting constrains experimental study duration and access to brain regions are limited. The study of other motor movements in humans is often supplemented by first studying simpler animal models such as non-human primates [16,17] and rodents [18–21]. However, these animal models fall short with respect to more complex freely generated motor sequences such as speech; this is primarily because none of the dominant models employed are capable of learning vocal behavior resembling the complexity of human speech [2,22]. For this reason, speech production studies, unlike other motor behavioral fields, have been limited exclusively to invasive [11,23,24,25] and non-invasive [26], clinical studies in humans.

Work in non-human primates and rodents have yielded physiological and engineering insights that enable the continued development of neurally driven upper limb prostheses for clinical functional restoration [4,16,27–32]. Given this track record of translation from animal models to clinical studies, despite anatomical differences in both limbs and brain structure, an animal model that exhibits similar vocal behavior, despite anatomical differences, could similarly benefit human speech prosthesis development. A natural candidate model is the songbird, which is widely used to study complex learned vocal behavior [33,34]. The zebra finch (*Taeniopygia guttata)*, in particular, is known for its precisely timed sequentially structured song which is stable over the course of its adult lifetime. Experiments with this system have yielded insights into the acquisition [35,36], maintenance [37,38], and generation [8,39,40] of complex motor-vocal sequences.

**Fig 1:**
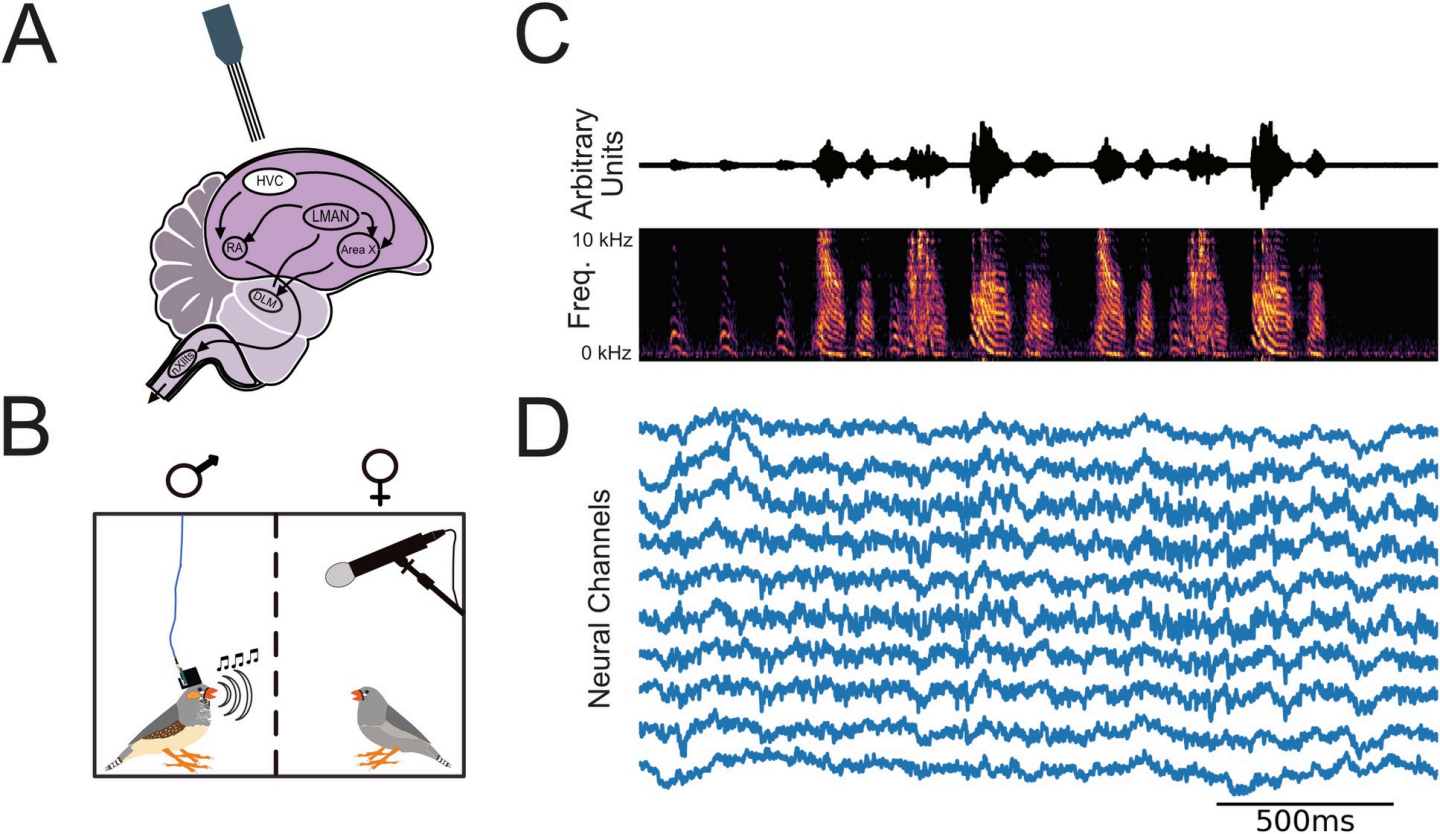
Continuous electrical and audio recording of chronically implanted freely behaving male zebra finch (Methods). (A) Four-Shank multi-site Si-Probes were chronically implanted targeting nucleus HVC. HVC provides input to two motor pathways that innervate neurons in the tracheosyringeal half of the hypoglosssal nucleus (nXIIts) that projects to vocal muscles. The posterior descending pathway (PDP) comprises a direct projection to the robust nucleus of the arcopallium (RA) and is necessary for both the acquisition and production of learned vocal behavior (song). The anterior forebrain pathway (AFP), provides an indirect pathway to RA, through Area X, the dorsolateral anterior thalamic nucleus (DLM), and LMAN, and is necessary for song acquisition. The PDP is homologous to a motor pathway in mammals that starts in the cerebral cortex and descends through the brain stem, while the AFP is homologous to a cortical pathway through the basal ganglia and thalamus. (B) Neural and audio recording apparatus. We recorded LFP and vocal activity from male zebra finches stimulated to sing by the presence of a female conspecific. (C) Exemplar sound pressure waveform (1.3 seconds in duration, top) from bird z007 above the corresponding spectrogram. (D) Voltage traces (μV) of ten randomly selected channels of simultaneously recorded neural activity aligned to the song during audio recording, after a 400 Hz low-pass filter.

Although the anatomical structure of avian and mammalian brains are divergent, relevant similarities between avian nuclei and human cortex have been identified. The premotor nucleus HVC, used as a proper name, contains neurons with sparse spiking patterns precisely aligned to vocal production [8,41]. Multiple studies suggest HVC is analogous to premotor cortex in human and non-human primates with respect to both functional [42–45] and genetic profile [46,47]. HVC provides input to two pathways that lead to neurons within nuclei that control the vocal muscles of the syrinx (Fig 1A). The first is directly through the arcopallium (RA) in the posterior descending pathway (PDP), which is necessary for both the acquisition and production of learned motor-vocal behavior. For reference to mammalian anatomy, the PDP is analogous to a motor pathway that starts in the cerebral cortex and descends through the brain stem. The second, named the anterior forebrain pathway (AFP), plays a strong role in acquisition and adjustment of vocal output throughout life and projects indirectly through several nuclei; it is analogous to a cortical pathway through the basal ganglia and thalamus in mammals [34]. These analogous regions share thousands of convergent genes despite their last shared ancestor being millions of years ago [47].

At a circuit level, the similarities between the neural activity in humans and songbirds is much harder to compare. Single-unit work and techniques, like those studied in songbirds, are difficult to conduct within a clinical setting. In contrast, given the accessibility of the system and a focus on fundamental physiology, songbird studies [6,10,34,35,37,38,40,44,48,49] have largely focused on carefully identified neural units and their spiking activity, with limited examination of other neural features that may correlate with vocalization behavior. Examining neural features readily accessible in both species and their relationship to motor-vocal behavior will enable direct comparison between the neural activity in birdsong and human speech production. Clarifying similarities (and differences) that may exist will bridge the gap between the two species.

Local field potentials (LFP) are thought to reflect both aggregate local postsynaptic activity and presynaptic inputs to the recording site [50]. As stable single units can be difficult to acquire and maintain in humans and non-human primates, there is a rich history of literature looking at both spiking and LFP for understanding, decoding, and predicting motor production [28,51–54]. At present, characterization of the neural dynamics of HVC and their relationship to behavior in songbirds has focused primarily on single- and multi-unit spiking activity [6,34,40,55,56], and limited work has focused on the structure of LFP and how it relates to song production. The most detailed characterization of this signal in songbirds is the relationship between LFP and interneuron synchrony [39]. This leaves a gap in the literature regarding the structure of LFP activity in HVC and whether its characteristics have any similarities to LFP in human, non-human primates, or mammalian premotor and motor regions.

We address this gap by chronically implanting and recording from freely behaving male zebra finch (Fig 1B) and analyzing LFP, presumptively from HVC, in relation to each bird’s performed song (Fig 1C and 1D). We identify narrow-band oscillations [57–59] similar to those reported in human, non-human primate, and rodent motor electrophysiology literature. Further we provide evidence that phase and amplitude modulation within these frequency bands is predictive of vocalization behavior.

## Results

**Fig 2:**
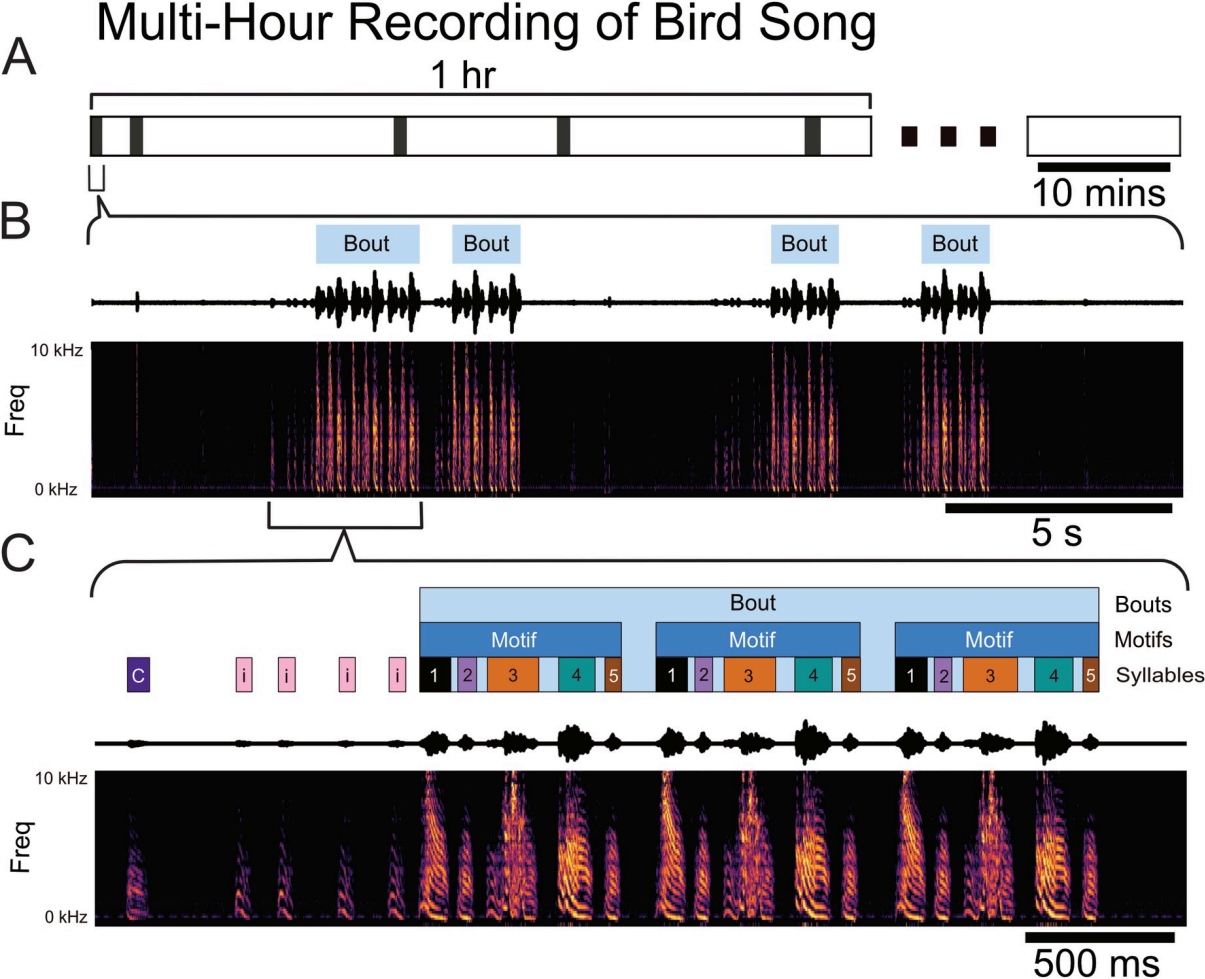
Temporal structure of vocal behavior. (A) Schematic showing the intermittent occurrence of vocally active periods (VAPs, black bar) at sporadic intervals throughout the first hour of a multiple-hours-long continuous recording session of one male zebra finch (z007). (B) Zoomed-in view of one 25-second-long VAP comprising several bouts, denoted by light-blue rectangles above the sound pressure waveform and corresponding spectrogram. (C) Zoomed-in view of the first bout in segment (B) showing introductory notes, typically repeated a variable number of times prior to the start of the bout and labeled as “’; other vocalizations not part of the courtship song, labeled ‘C’; and syllables comprising the courtship song, labeled ‘1’, ‘2’, ‘3’, ‘4’, and ‘5’, based on their sequential order in the motif.

**Fig 3:**
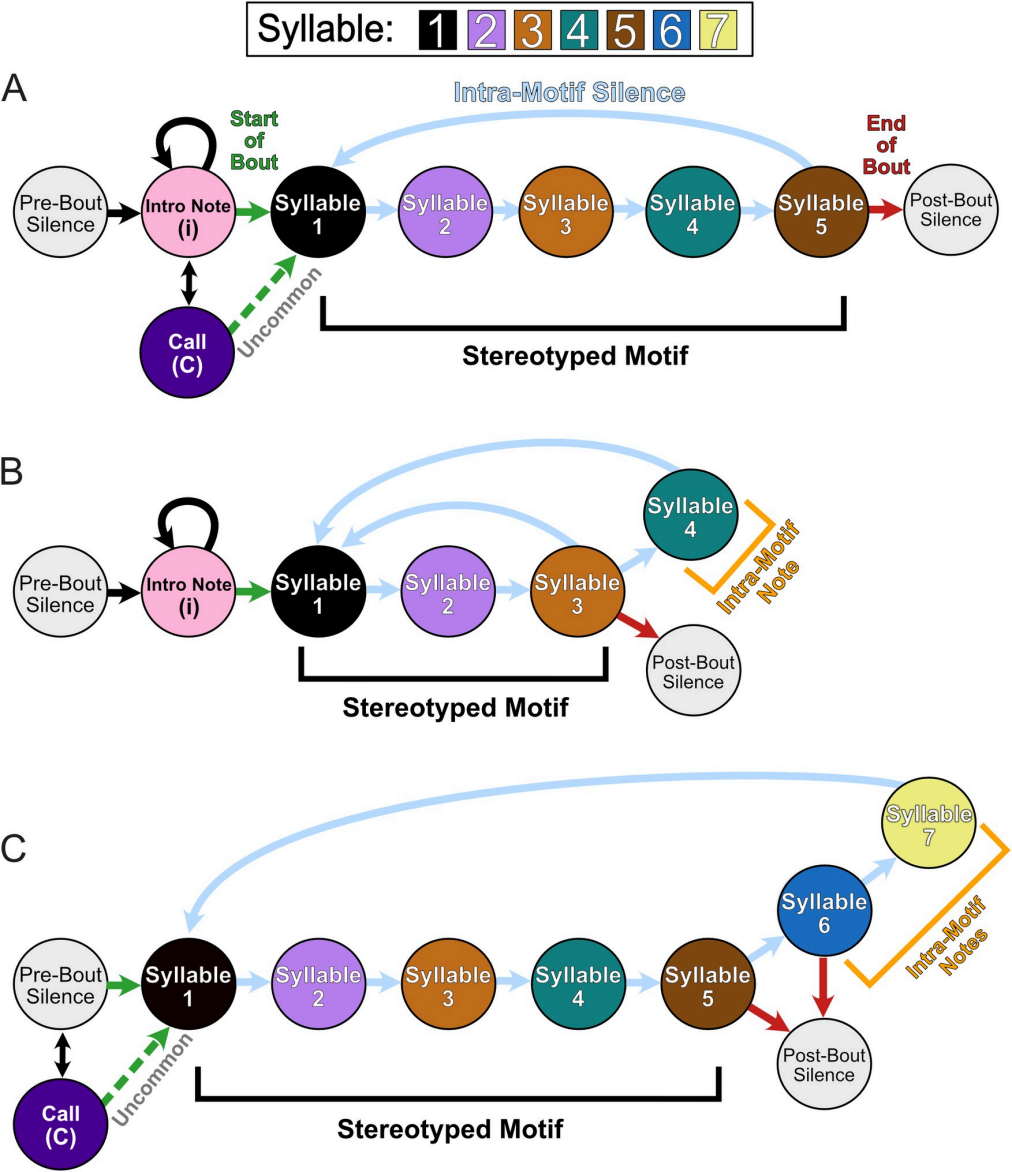
State Diagram of All Subjects Observed Song Behavior during High Yield Days. (A) Schematic showing the vocalization structure of subject z007’s song. (B) Schematic showing the vocalization structure of subject z020’s song, including its intra-motif note syllable 4. (C) Schematic showing the vocalization structure of subject z017’s song, including both of its intra-motif notes, syllable 6 and 7. For all three diagrams the green arrow represents the transition into the stereotyped motif song behavior; this sequence is underlined by a black bracket. The red arrow indicates the termination of the repeated motif sequence and the end of the bout.

Adult male zebra finches (n = 3) were chronically implanted with laminar silicone microelectrode probes (Fig 1A). Local field potentials from these probes and vocal behavior from a microphone were simultaneously recorded (Fig 1B–1D) (see Methods). All references to LFP localization are recognized to be presumptively from HVC. The male zebra finches’ learned song is structured and was annotated (Fig 2). The song consists of 1–7 repetitions of a motif, each of which is composed of a precisely ordered sequence of 3–10 discrete units called syllables. Beyond the syllables of the motif, male zebra finches may have an additional syllable or sequence of syllables that they will optionally insert between successive repetitions of motifs. These are called “connector” [60] or intra-motif notes. Song motifs are also grouped into larger structures called bouts, which consist of multiple repetitions of motifs. The bout is typically preceded by a variable number of repetitions of the same note, called Introductory Notes. Syllables of each bird’s learned song, other non-learned vocalizations, and tokens of non-vocal intervals were segmented and annotated within periods of vocal behavior, referred to as vocally active periods (VAP), using acoustic landmarks (Fig 2B and 2C). Fig 3 provides state diagrams that describe the specific transitions between syllables and other vocalization behaviors observed for all three of the subjects’ VAP (colloquially, their song’s “grammar”). Temporal boundaries of VAPs were used to define behaviorally relevant epochs in the simultaneously recorded neural signal. Subjects were recorded for 1–10 hours per day; statistics of recorded behavior data are documented in S1 and S2 Tables. For statistical power, we selected the two recording session days with the most vocal activity from each bird for the analyses reported here (S3–S5 Tables). Results from days with fewer recorded motifs were qualitatively similar.

### Song-related LFP spectral changes in HVC

**Fig 4:**
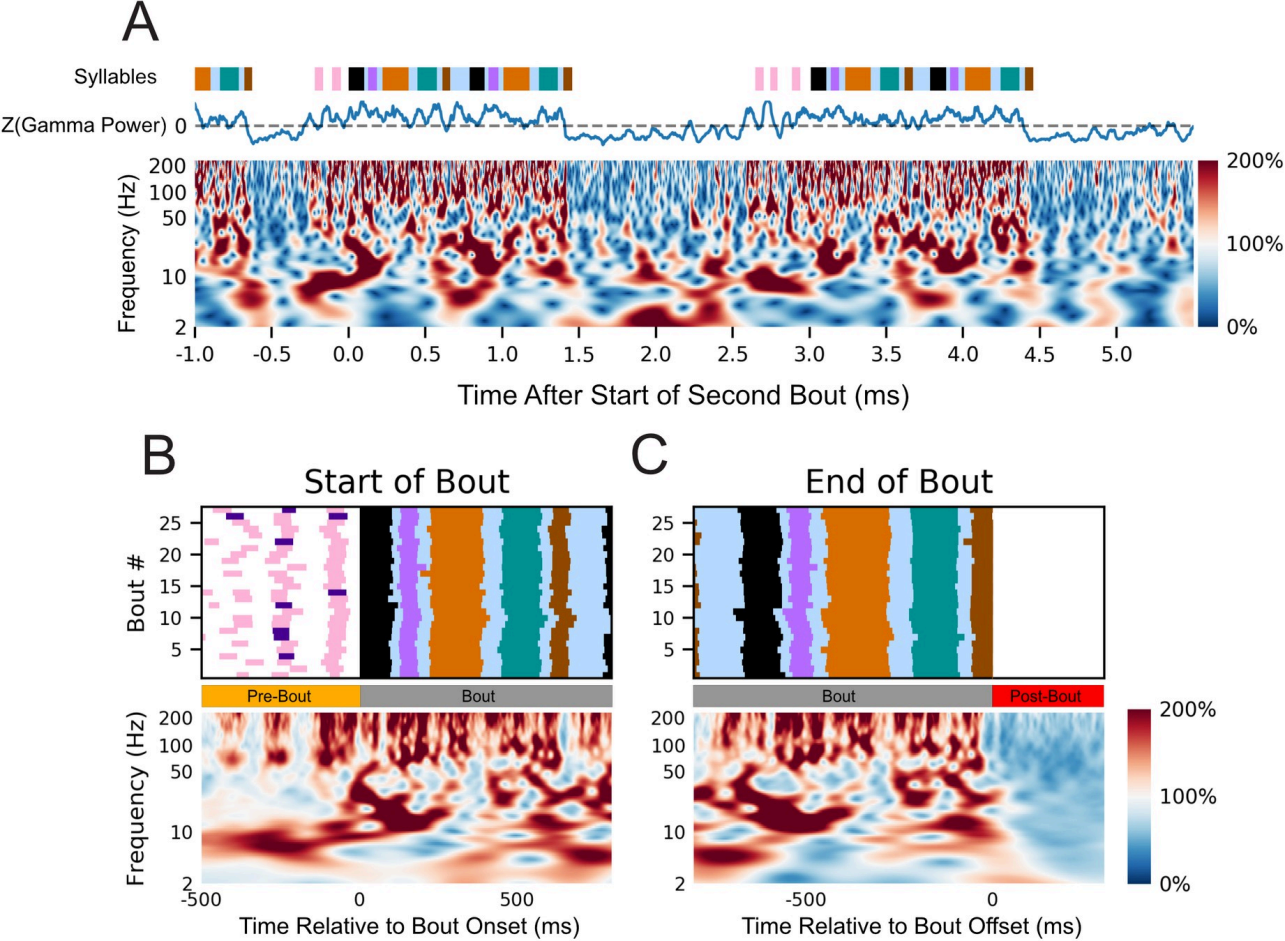
Variation in HVC LFP power correlate with vocal behavior. (A) Normalized spectrogram (bottom) of LFP activity from one representative channel (see Methods) aligned to the free vocal performance of a male zebra finch (z007) during the production of three separate bouts. Each frequency is scaled to its percentage of the mean amplitude during the respective VAP. Above the spectrogram is the z-scored power of the 50–200 Hz band smoothed with a 50ms rolling mean window aligned to the same behaviors. Colored bars above the spectrum annotate the vocal behavior, with color representing a distinct syllable with the same color-coding as Fig **2**. (B) Normalized spectrogram (bottom) from the same representative channel as (A) averaged across multiple renditions of similar bouts (n = 27, top) aligned to the start of the first motif in the bout. (C) As in (B) but aligned to the end of the last motif in the bout. Above each is a behavioral raster showing the time course of the behavior being averaged (n = 27 bouts). No dynamic-time warping was used, and all motifs were from the highest yield day for the subject. To ensure the start and end of the bout are unique time periods, only bouts with more than one motif in duration were used. Behaviorally inconsistent bouts were excluded for clarity of visualization; including them does not qualitatively alter the result.

Fig 4A illustrates the spectrotemporal characteristics of the LFP amplitude during the preparation and production of bird song, with an exemplar single trial time-varying power spectral density (PSD) during the course of three separate bouts. For each frequency, power is scaled to percentage of the mean amplitude during the respective VAP (see Methods). Through visual inspection of individual trials, relative to the post-bout period we noted a broadband increase in power in all frequencies above roughly 50 Hz prior-to and during vocal production (S3 Fig). This occurs simultaneously with rhythmic changes in frequencies below roughly 50Hz (i.e. bout aligned increases and decreases in amplitude). When assessing single trials, we noted that changes in LFP amplitude appeared to correspond with each instance of vocal behavior, including non-learned portions such as introductory notes. To evaluate the consistency of these changes in amplitude, we used the one-sided z-test to compare the distributions of power values during vocalizations to the distribution of power values during silence. A statistically significant increase in power for frequency bands above 50 Hz was seen across most, if not all, channels for all but one of the high yield days for z020 (S6 and S7 Tables). Changes in amplitude for frequency bands below 50 Hz were more nuanced, with no consistent trend across subjects; however, frequency changes tended to be consistent across days for two of the birds, particularly z017 and z007 (S6 and S7 Tables).

The spectral structure of the LFP activity immediately prior to and during the production of learned song is highly consistent across bouts, as suggested when viewed across time for multiple bouts (Fig 4A) and when they are aligned to their initiation and termination (Figs 4B and 4C and S2). Visualizations of activity around the times of bout initiation and termination were calculated by aligning to the start of the first syllable of the first motif in the bout (initiation) and the start of the last syllable of the last motif of the bout (termination) for all bouts recorded over the course of a single day. The aligned neural activity was then averaged for each time-frequency sample. These spectral structures were validated as a strong summary representation of the activity during the transition into and out of the stereotyped motif, despite trial-by-trial variability, by a modified z-score method (S2 Fig) (see Methods). As with the single trial time-varying PSD, there is an increase in amplitude for all frequencies above roughly 50 Hz (S1 and S3 Figs). The amplitude of these high frequencies were found to significantly decrease during the 100 millisecond window immediately after the end of the bout for most if not all channels for all days; with the second high-yield day of z020 being the only exception (S8 Table). Within subject, there was strong stereotyped structure for frequency bands below 50 Hz arising from co-occurrences in changes from both phase and amplitude. S4 and S5 Figs illustrate this stereotypy of time correlated spectral structure in HVC as it relates to the preparation and production of bird song. No dynamic time warping was used to align the behavior.

As in previous work [37,39], we found a strongly stereotyped 25–35 Hz power that was consistent across renditions of motifs (S4 and S5 Figs). In addition to this previously reported frequency band, structured activity was observed in several frequency ranges that are commonly studied in mammalian literature, namely 4–8 Hz, 8–12 Hz, and 35–50 Hz. This structure, observed to be stable across trials, arises from fine time-alignment of both phase and amplitude, and occurred over times-scales longer than a single cycle for a given frequency below approximately 50 Hz (S4 and S5 Figs). These oscillations, with consistency in both phase and amplitude, were observed to start just prior to song onset and to end immediately after vocal production stopped (S5 Fig). Similar patterns emerged in each of the birds and within birds across days (S4 and S5 Figs) [39]. This observation led us to ask: What frequencies in the LFP might carry information about the preparation and production of song through either phase or amplitude changes?

### Decoupling LFP power-spectrum reveals song related spectral features

**Fig 5:**
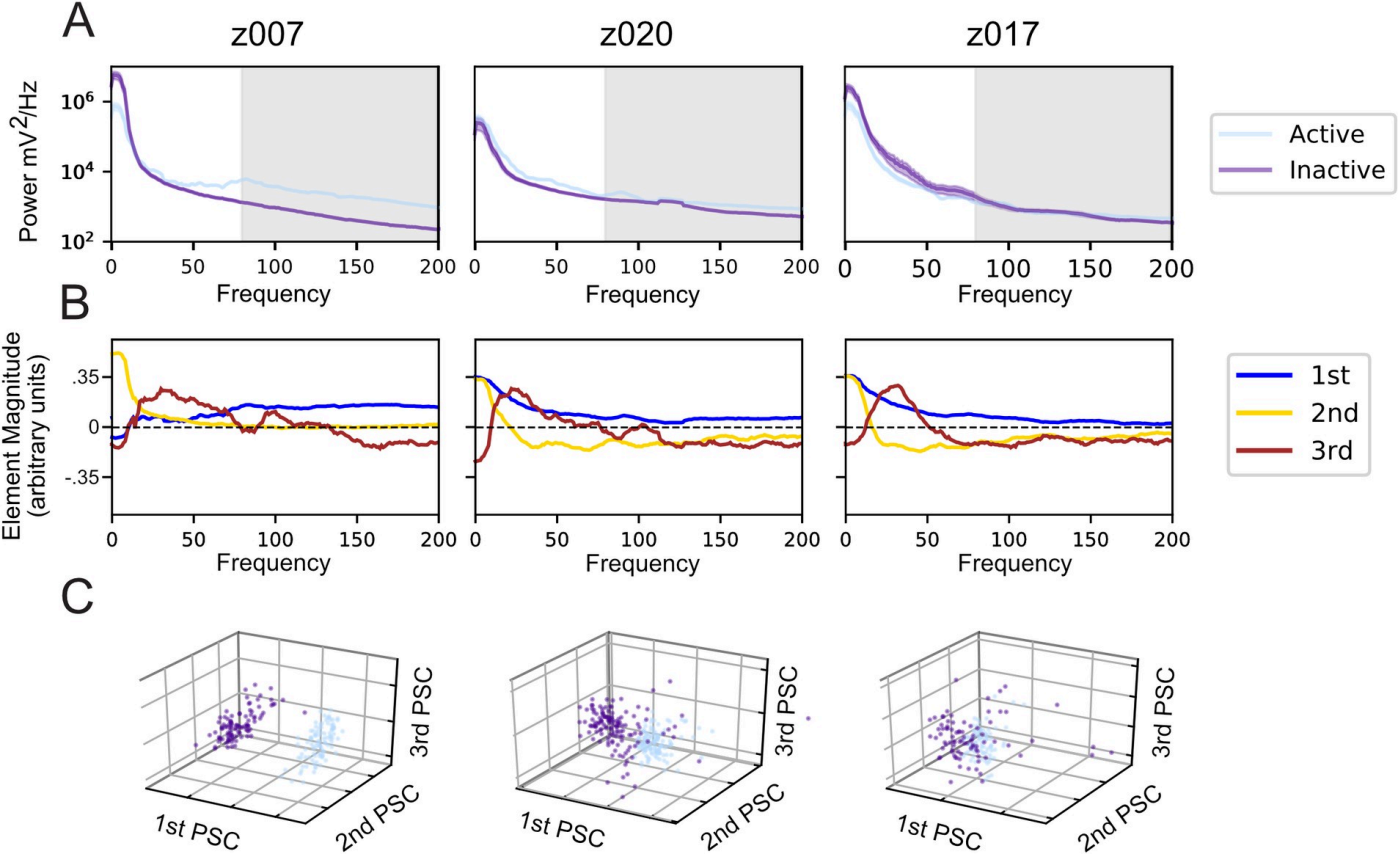
Naïve decomposition of LFP power spectra reveals song correlated features. Representative results from a single electrode channel for each of the three subjects during its highest yield day. (A) Averaged power spectra during trials centered in 1-second intervals where the bird either initiates a motif, called vocally active (light blue), or does not vocalize, called vocally inactive (purple). The 80–200 Hz, or ‘High Gamma’ band, is shaded in grey. (B) The power spectra in Fig **4**A are normalized and naively decomposed into PSCs (see Methods). The elements of the first principal spectral component (1st PSC, blue) are non-zero across all frequencies, likely due to the power law in LFP PSDs. The 2nd PSC, golden-yellow, peaks between 0 and 10 Hz. The 3rd PSC, burgundy, peaks between 10 and 30 Hz, but has variations across birds that extend into 50Hz. As the PSCs are all eigenvectors, their signs do not matter when interpreting them. This structure is largely consistent across channels and across days. (C) Projection of both the vocally active and vocally inactive trials onto the first three PSCs for the same channels in (A) and (B). The color coding is the same as (A). These projections include 84 trials per condition for z017, 100 trials per condition for z007, and 133 trials per condition for z020.

A spectral decomposition technique previously applied to human motor cortex data [61] was used to determine which frequencies’ amplitude changes were correlated with the production of birdsong. As suggested in Fig 5A and statistically assessed in S6 Table, during times of vocal activity for all birds we observe an increase in power aggregated across higher frequencies that are in a range often referred to as “High Gamma” [62,63]. The frequency ranges for “High Gamma” vary in the literature, but Miller et. al. described it as 80-200Hz. Following the steps of the spectral decomposition approach described in the Methods of Miller et. al., the principal component decomposition of these PSDs found principal spectral components (PSC) similar to those previously reported in [61] (Fig 5B and 5C). The PSC’s shown in Fig 5 are results from one channel, which was representative of all channels for each subject. The first PSC, or the most significant principal component, in Miller et al. is characterized for having most of its element magnitudes with the same sign and being consistently non-zero, with values approaching a constant across frequencies. This was subsequently described as reflecting a broad spectrum increase in power most clearly visible in High Gamma. In our analysis the most consistent PSC, which is the first principal component, was a spectrum wide-amplitude change that was consistent across channels and subjects (S6A Fig). The second PSC in Miller et al. peaked in the “alpha/low beta range”—alpha is described as 8–12 Hz and beta is described as 12–30 Hz—which mirrors PSC 3 found across subjects and channels in zebra finches (S6C Fig) [61]. These frequency bands in humans have been proposed to reflect the resting rhythms that decrease when cortical areas are activated [64]. The characteristic structure of each PSC was found to be consistent across subjects, channels, and days using the cosine similarity metric (see Methods) (S7 Fig). Cosine similarity is a measure of the degree of similarity of two non-zero vectors of an inner product space. It evaluates their orientation—and not their magnitude—with two vectors that have the exact same orientation having a value of 1 and two vectors that are orthogonal to one another having a value of 0. In addition, with this metric we also found that the similarity of each PSC to another across subjects was greater than was the case with a different PSC (S7E–S7G Fig). Although the PSCs were calculated without explicit knowledge of distinct vocally active and inactive time periods, the first three PSCs show that the power spectrum during vocal production is separable from vocally inactive periods (Fig 5C). This separation between behavioral states was evaluated using the sensitivity index, or d’, between the two behavior states; each channel was analyzed independently (S8A Fig). The sensitivity index for each PSC was compared to an estimate of the null distribution calculated via permutation analysis (n = 20,000) in which the state labels were shuffled (S8 Fig); the Benjamini-Hochberg False Discovery Rate (p<0.05 and q<0.05) was applied to the results of this permutation test to account for multiple comparisons across all of the channels (S9 Table). For all birds, channels with PSCs that have significant sensitivity indexes were found. The percentage of channels with significant sensitivity indexes varied across birds and PSCs; for the first three PSCs, channel percentages were as low as 31.25% and as high as 100%. The behavioral state dependent separation in the PSCs, which are calculated without explicit knowledge of the state labels, suggests that the frequencies scaled by the PSCs could be used to detect the onset of vocal production.

### Syllable onset is phase-aligned to underlying LFP rhythms

**Fig 6:**
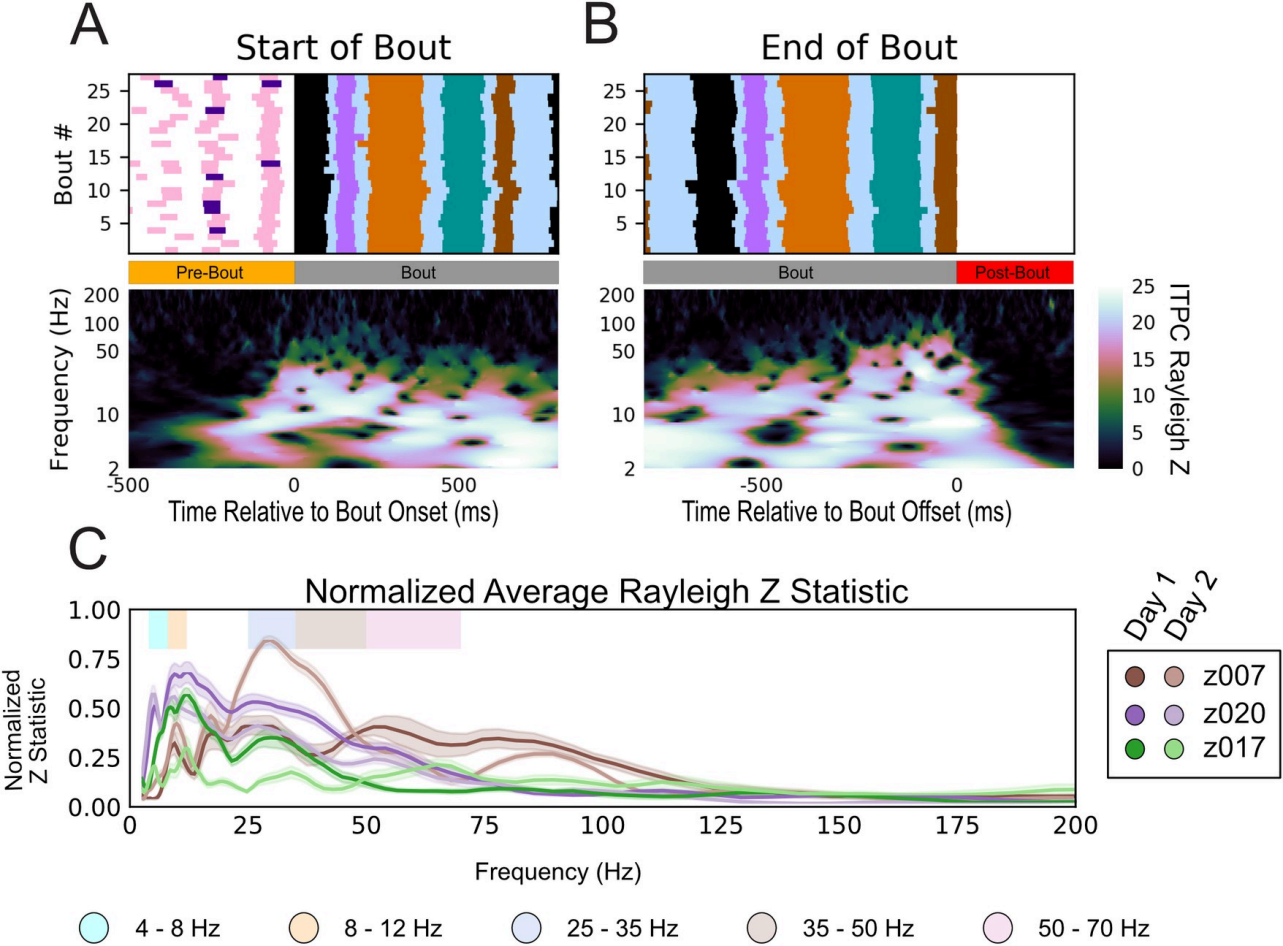
LFP Inter-trial phase coherence during production of learned sequences. (A) ITPC of LFP aligned to the start of the first motif of the bout for bird z007 (bottom) across multiple renditions of similar bouts (n = 27, top) aligned to the start of the first motif in the bout. (B) the same as (a) aligned to the end of the last motif of the bout. No dynamic-time warping was used. To ensure that the start and end of the bout are unique time periods, only bouts with more than one motif in duration were used. Behaviorally inconsistent bouts were excluded for clarity of visualization; however, including these bouts does not alter the result. (p<0.006 for all Rayleigh Z > 5; all non-black time-frequency points in this plot are above the significance threshold). (C) Normalized sustained Z-statistic of the ITPC for samples preceding the labeled start of the bout. The steps for calculating this metric are detailed in the Methods. Data reflect all bouts from both high-yield days for three birds.

We next calculated the inter-trial phase coherence (ITPC) of the spectrum (Methods) from 2–200 Hz to determine which frequencies had stable song-aligned structured changes in phase. The ITPC is a measure of the phase synchronization of neural activity in a given frequency at a specific time sample. A defining characteristic of zebra finch song is its stereotyped motif. Leveraging this stereotypy, we calculated the ITPC aligned to the first motif of all bouts recorded in a single day (n>28). Any LFP phase coherence occurring prior to song production can be attributed to the preparation of the subsequent vocalization. In two of the three birds, the first motif was almost always preceded by an introductory note. As each frequency and time sample are separate results with an attributed p-value, they are all visualized in terms of their Rayleigh Z statistic (see Methods) to allow for comparisons. By scaling the p-values of each independent ITPC result, the Rayleigh Z statistic provides a measure by which multiple comparisons in circular statistics can be accounted. Based upon the number of trials and conditions tested, a threshold value can be applied to the Rayleigh Z statistic to determine statistical significance. Fig 6A shows long-lasting phase structure that precedes the onset of the bout by up to 100 ms in frequencies lower than 50 Hz (note that in this figure and in all subsequent ITPC Rayleigh Z statistic images we have set all non-significant times and frequencies to black based upon the appropriate threshold value for the condition evaluated). This consistency in phase continues during the course of the bout and terminates soon after the end of the bout (Fig 6B). Although the precise frequency ranges vary across subjects, the subject specific pattern present prior to bout onset and sustained throughout the bout was consistently observed across channels, subjects, and days (S9 Fig).

To determine the frequencies where phase coherence is most consistent across individual subjects, we computed the normalized sustained Rayleigh Z Statistic (Methods) for the two high-yield days for all subjects (Fig 6C). The frequencies that contained sustained high ITPC values across animals include the 25–35 Hz range, as previously established [37,39], and several other frequencies: 4–8 Hz, 8–12 Hz, 35–50 Hz, and 50–70 Hz. These oscillations fall within well-documented frequency ranges in mammalian literature, namely theta (approximately 4–8 Hz), alpha (approximately 8–12 Hz), and low gamma (approximately 30–70 Hz) [65]. The lower frequencies exhibited longer periods of phase coherence through time than did the higher frequencies. These periods occurred over several cycles, and they fell out of alignment faster than a full cycle once the song terminated (Figs 6B and S9). Strong phase coherence was observed throughout the production of the motif without the need for dynamic time warping to force motifs into alignment on a warped time scale (Figs 6 and S9). However, when viewing the ITPC in Fig 6 across the longer time scale of the bout, it is important to note that there is considerable variation in the timing of individual renditions of the bout. If the dynamics of LFP phase are aligned to the time scale of underlying behavior, then the variation in motif timing across bouts will result in a reduction in ITPC at time points further away from the point of alignment. In particular, such a reduction will be more pronounced for higher frequencies which have shorter cycle times.

**Fig 7:**
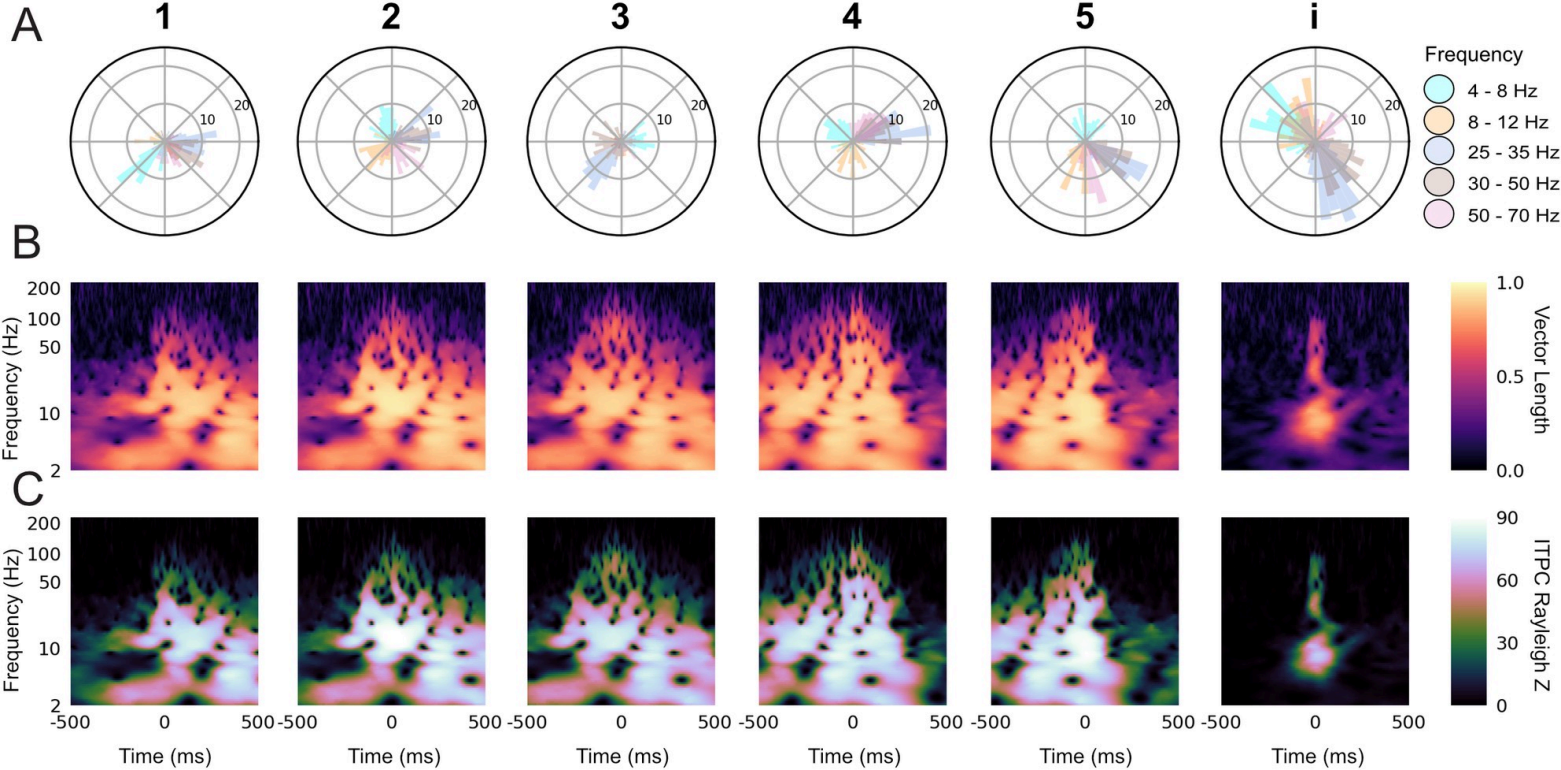
Syllable specific inter-trial phase coherence reveals phase preference to syllable onset. (A) Polar histogram of the phase for each LFP frequency band at the labeled start of all instances of a given syllable or the introductory note over the course of one day (Day 2), for bird z007 (S3 Table). (B) ITPC resultant vector length, indicating the level of phase consistency across trials, for each frequency over time relative to the labeled start of each syllable or introductory note (0 ms) over randomly downselected instances from (A) to match the number of instances per syllable. (C) Rayleigh Z-statistic of the ITPC over the same time and frequencies as (B) (p<0.007 for all Z > 5 for all syllables; all non-black time-frequency points in these plots are above the significance threshold). For (B) and (C) the number of instances (n = 98) are equal for all syllables and the introductory note, and is set by the syllable class with the fewest renditions.

**Fig 8:**
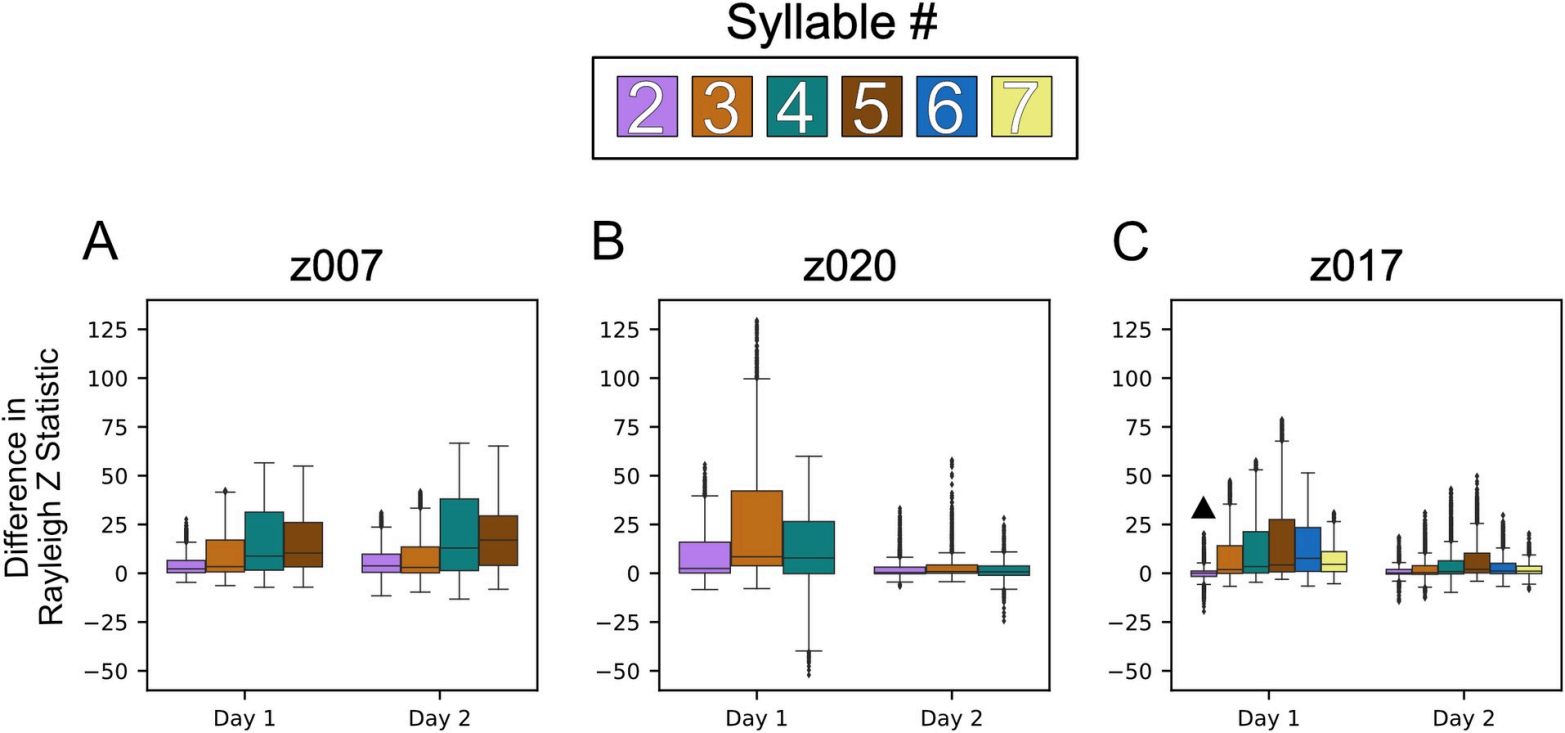
Phase preference to syllable onset is reflective of constitutive syllables rather than of the larger motif structure. Box plots of the differences in Rayleigh Statistic between the syllable aligned ITPC and the ITPC aligned to that same syllable’s stereotyped onset time within the motif. Positive differences indicate greater phase consistency at the syllable aligned time versus that of the stereotyped time across LFP frequencies (4–8 Hz, 8–12 Hz, 25–35 Hz, 35–50 Hz, and 50–70 Hz). This difference was determined by first concatenating the Z statistic for each channel centered at the designated time point to get a vector of all partially overlapping frequencies for all channels. The vector of stereotyped alignment was then subtracted from the labeled onset alignment to achieve the difference for each frequency on every channel. This was repeated for all syllables, excluding the first, with each syllable represented by a specific color, as indicated. (A) Shows the results for the two high-yield days for z007, (B) shows the results for z020, and (C) shows the results for z017. All instances of each syllable that were preceded by the first syllable were used. To determine statistical significance, we used the one-sided Wilcoxon signed-rank test, with each frequency and channel pair. ^▲^ denotes that the comparison was not statistically significant when using the using the Benjamini-Hochberg False Discovery Rate. All other results p<0.05 and q<0.05.

To better understand the dynamics underlying HVC population activity within the bout, we examined the song-aligned LFP phase coherence in greater detail. One hypothesis, given the highly stereotyped spectro-temporal structure of the motif including the brief gaps between each syllable, is that both the vocal output and the underlying HVC dynamics are largely deterministic (i.e., the observed coherence may reflect an initial, single alignment at the start of the motif, which endures only by virtue of the very low song-to-song variability). Alternatively, it could be possible that HVC population activity reflects a more tightly controlled timescale, in which the observed oscillations are aligned to shorter timescale vocal events such as syllables. Previous work has shown that local spiking activity in HVC is aligned to syllables not motif onset [55,56], however as LFP largely reflects postsynaptic and transmembrane currents coming into the region of the recording the site it is unknown whether LFP shares this temporal alignment. To determine whether alignment is tied to the motifs (hypothesis one), or is unique to each syllable (hypothesis two), a smaller scale time alignment was used. We first verified that phase-locking was found when localized to shorter sub-components of vocalization. Syllable-specific polar phase analysis (Fig 7A) shows a strong phase preference to the onset of each syllable in all of the previously determined frequency bands of interest. These phases were unique to each frequency band for each syllable. Similar results were seen for all subjects across all days (S10–S13 Figs). We also found the same phase preference to vocalization onset for the introductory note despite its huge variability in duration and structure beyond a single utterance. Fig 7C demonstrates that this stereotyped phase structure occurs prior to and during each vocalization and is statistically unlikely to have occurred by chance. To directly test the two hypotheses, we compared, relative to the onset time of the first syllable, the ITPC centered on onset time of each subsequent syllable to the ITPC centered on the stereotyped time of the same syllable (Fig 8). Centering on the actual labeled syllable start time yielded significantly stronger ITPC compared to centering on the stereotyped, or across-bout average, start time for each syllable (S10–S13 Figs). Thus, the result of this analysis supports hypothesis two as the more likely description of this phase locking characteristic.

### LFP features encode intended syllable identity

**Fig 9:**
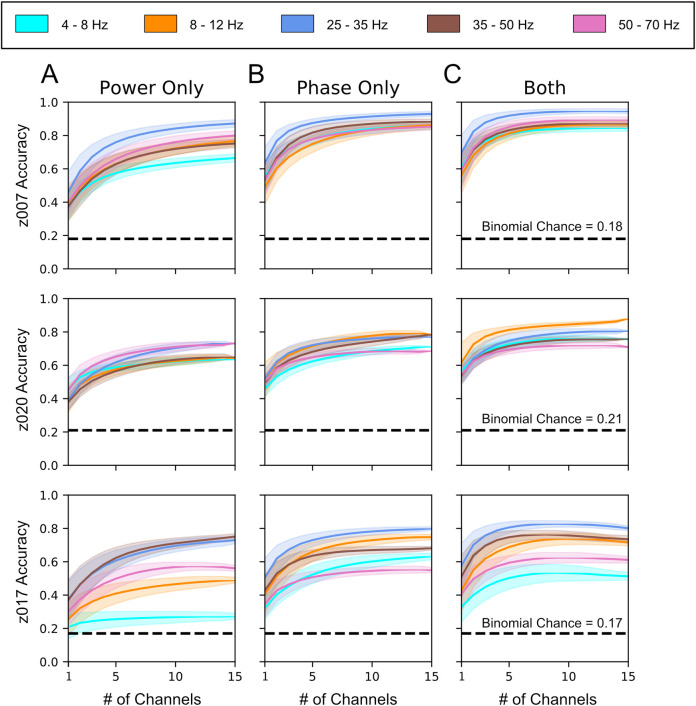
LFP phase and power provide independent and additive information for vocalization classification. Channel-adding curves calculated by repeatedly training classifiers with an increasing number of randomly selected channels (see Methods). (A-C) Channel-adding curves showing classifier performances with either (A) all LFP phase information removed, (B) all LFP power information removed, or (C) with both phase and power used as independent features. Each row corresponds to data from the highest-yield day for each bird. These vocal and non-vocal classification events are collectively referred to as classes (see Methods). z007 n = 98 for each of 7 classes (1, 2, 3, 4, 5, i (introductory note), Silence); z020 n = 91 for each of 6 classes (1, 2, 3, 4, i (introductory note), Silence); and for z017 n = 52* for each 8 classes (1, 2, 3, 4, 5, 6, 7, Silence). The results of up to 15 channels are shown to allow for direct comparison across subjects. Dark lines show the mean for each vocalization class, shaded areas give the standard deviation over the bootstrapped analysis using n = 5,000 repetitions across 5 cross-validation folds. The p-value for all of the binomial chances calculated for each bird was 0.05. *The number of instances for each class was limited by Syllable 7, which is an intra-motif note.

Having isolated potential LFP bands of interest through the decomposition of both phase and power, we next asked whether the LFP bands’ spectral features were correlated to vocalization identity. If so, then these features could be used to classify vocalization identity from neural activity alone. As the dominant characteristic of these features are their consistent structure, a promising approach was to create frequency band-specific LFP templates that could be correlated with representative time traces. It was unclear, however, what the ideal time range and latency relative to song onset for this information might be. With the goal of better understanding the ideal time range and latency, we conducted a hyperparameter search using a linear discriminant analysis (LDA) model and singular value decomposition (SVD) (see Methods). The classifier was trained with LFP templates for which the duration in samples (bin width) and the time between the event onset and the end of the template (offset) was varied in the hyperparameter search. All templates considered fully precede the onset of the event. For most frequencies found, multiple combinations of bin width and offset yielded classifiers that could distinguish, significantly above binomial chance, between song syllables, silent (non-vocal) periods, and—for the subjects that performed them—introductory notes (z020 & z007) (Figs 9 and S16). As classifiers were trained to distinguish both vocal and non-vocal events, these vocalization event types are collectively referred to as classes (see Methods). The parameter search for each frequency was performed separately, and, although there was considerable variation, the best bin width and absolute value of offset tended to decrease as LFP frequency increased. S10 and S11 Tables summarize the parameters in the search that yielded the highest classification accuracy. Nearly all frequencies we examined were useful for classifying prior neural activity as it relates to the song it will produce downstream; however, it is not immediately clear what component of these oscillations (i.e., phase or amplitude), carries information regarding song identity.

To determine which component (phase or amplitude) of each frequency band provided information about the vocalization identity, we applied the Hilbert transform to selectively remove either phase or power, while retaining only the contributions of power or phase information, respectively. Information provided by a given component was inferred from classifier accuracy. As shown in Fig 9A–9C, both phase- and amplitude-based classifiers had accuracies above binomial chance for all frequencies; from this, we inferred that they each carried song-related information. In general, however, phase had higher classification accuracy than power for the frequency bands below 50 Hz. This difference in accuracy between phase and power was greater for lower frequencies (i.e., those below 30 Hz). The highest frequency band, 50–70 Hz, had marginally higher performance for power-only than for phase-only. We also queried if the number of channels recorded were necessary for decoding song identity by running a channel-adding analysis for each frequency band (see Methods). This analysis evaluates improvements in classifier performance enabled by increasing the number of recording sites on the probe and provides insight into the point beyond which additional electrodes provide diminishing returns. As shown in Fig 9A, the LDA classifier performed well above binomial chance with only 5–10 channels of neural activity when classifying between song syllables, silence and introductory notes.

### LFP features predict syllable onsets

**Fig 10:**
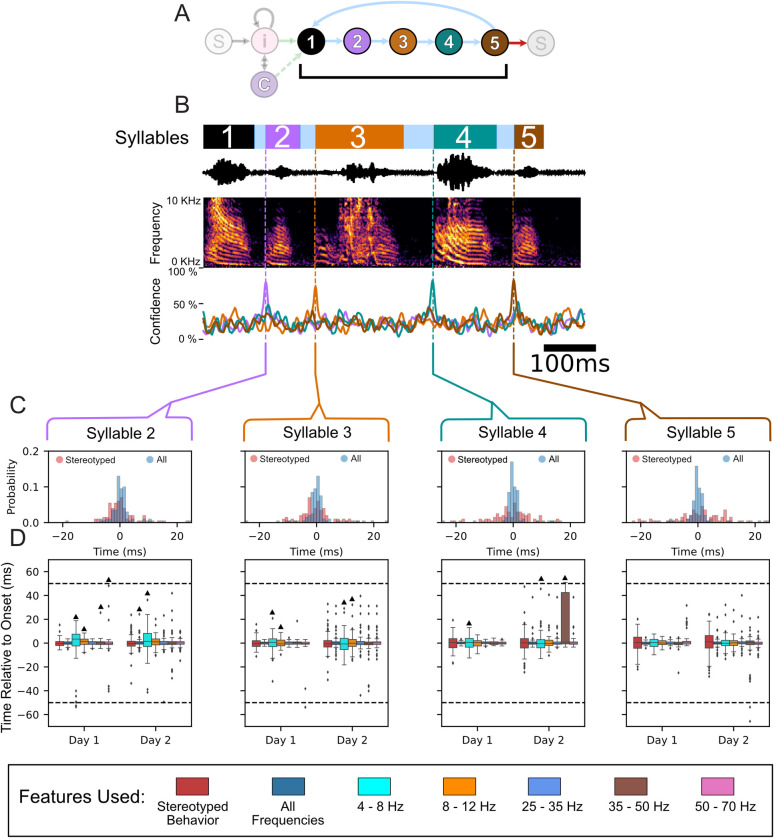
Onset detection using LFP features for Subject z007. (A) State diagram of z007’s observed song structure. Syllable colors are the same as in Figs **2** and **3**. (B) Example motif from the highest-yield day for subject z007. Annotated behavior (top) using the same color scheme as in Fig **2**, sound pressure waveform and the corresponding time-aligned spectrogram (middle), and the time-varying naïve confidence of the onset prediction (bottom) for each syllable in this example motif. Confidence signal traces are the same color as the syllable they are meant to predict. (B) Histogram of onset prediction times for each syllable relative to its annotated start time. The annotated start times are relative to the start of the first syllable of the motif that the syllable occurred in. The histogram compares two approaches: The first, in pink, uses only behavioral information (the stereotyped onset relative to the start of the first syllable), and the second, in blue, uses all the neural features to predict the start of the syllable. (C) Boxplot of onset prediction times relative to the labeled onset time for both of the high-yield days for z007. The order of each feature used is the same, going left to right: first is the stereotyped onset time using only the deterministic behavior (colored red), next are the results using all of the neural features (colored navy blue), then each frequency band only in order from least to greatest (4–8 Hz, 8–12 Hz, 25–35 Hz, 35–50 Hz, and 50–70 Hz). The time window that the neural based predictor must make a prediction within is represented by the dotted black line represents (see Methods). Statistical significance was calculated using the one sided Wilcoxon signed-rank test, and ^▲^ denotes results that are not statistically significant when using the Benjamini-Hochberg False Discovery Rate. All other results p<0.05 and q<0.05.

The demonstrated ability to accurately classify syllable identity with LFP features could be a consequence of a unique LFP signal structure associated with specific syllables or could be a consequence of a motif level LFP signal structure and stereotypy in the syllable onset time within the motif. To disambiguate these two possibilities, we examined whether the template features optimized for determining syllable identity (S10 and S11 Tables) could also predict specific onset times of each separate vocalization within the full motif. To accomplish this task, we implemented a naive pattern-matching approach (Methods). As the syllables’ onset is predicted using features derived from causal bandpass filters and samples that occur prior to their start, the features are based entirely upon signals recorded prior to syllable onset. As the stereotyped structure of the zebra finch’s song is well documented, we used the mean error between the stereotyped (average across trials) start time of each syllable—relative to its first syllable—and the actual time the syllable occurred as a benchmark to test the performance of a predictor that used the LFP features described previously. The observed behavioral variability in onset timing for each syllable relative to the first is detailed in S3–S5 Tables. An example of one motif and its corresponding confidences is shown in Fig **10**A. Across all syllables for all birds, the predictor that used all of the frequencies performed better than predication based upon the stereotyped onset of the syllable, Figs **10**B and S18–S20. Similar results were found for the 2 additional syllables for z017 as well (S20 Fig). Statistically significance was assessed based upon the Wilcoxon signed rank test and the Benjamini-Hochberg false discovery rate (FDR) correction (p<0.05; q<0.05). The paired test directly compared the error in onset time prediction between the neural predictor and the behaviorally based stereotyped start time to evaluated if the neural-based predictor was more accurate. When applying the FDR correction, the higher performance achieved by the all frequencies LFP predictor over the stereotyped onset time predictor was statistically significant for all syllables across all birds and high-yield days except for only three cases out of the 26 tested; these were syllables 2 & 4 for z020’s 2nd high-yield day and syllable 2 for z017’s 2nd high-yield day (S20 Fig).

We next asked which frequency, if any, might be best at predicting the onset time of the syllables. As demonstrated in Figs 10C and S20, no single frequency performs best for predicting onset times across all frequencies and all subjects. Although the higher frequencies tend to perform better with less variance than the lower frequencies, using all of the frequencies yields better performance than any one frequency across all subjects and sessions. There were poor prediction performances for 4–8 Hz, 8–12 Hz, and the 50–70 Hz for some syllables for certain birds with multiple examples of performance lower than that of the stereotyped onset.

**Fig 11:**
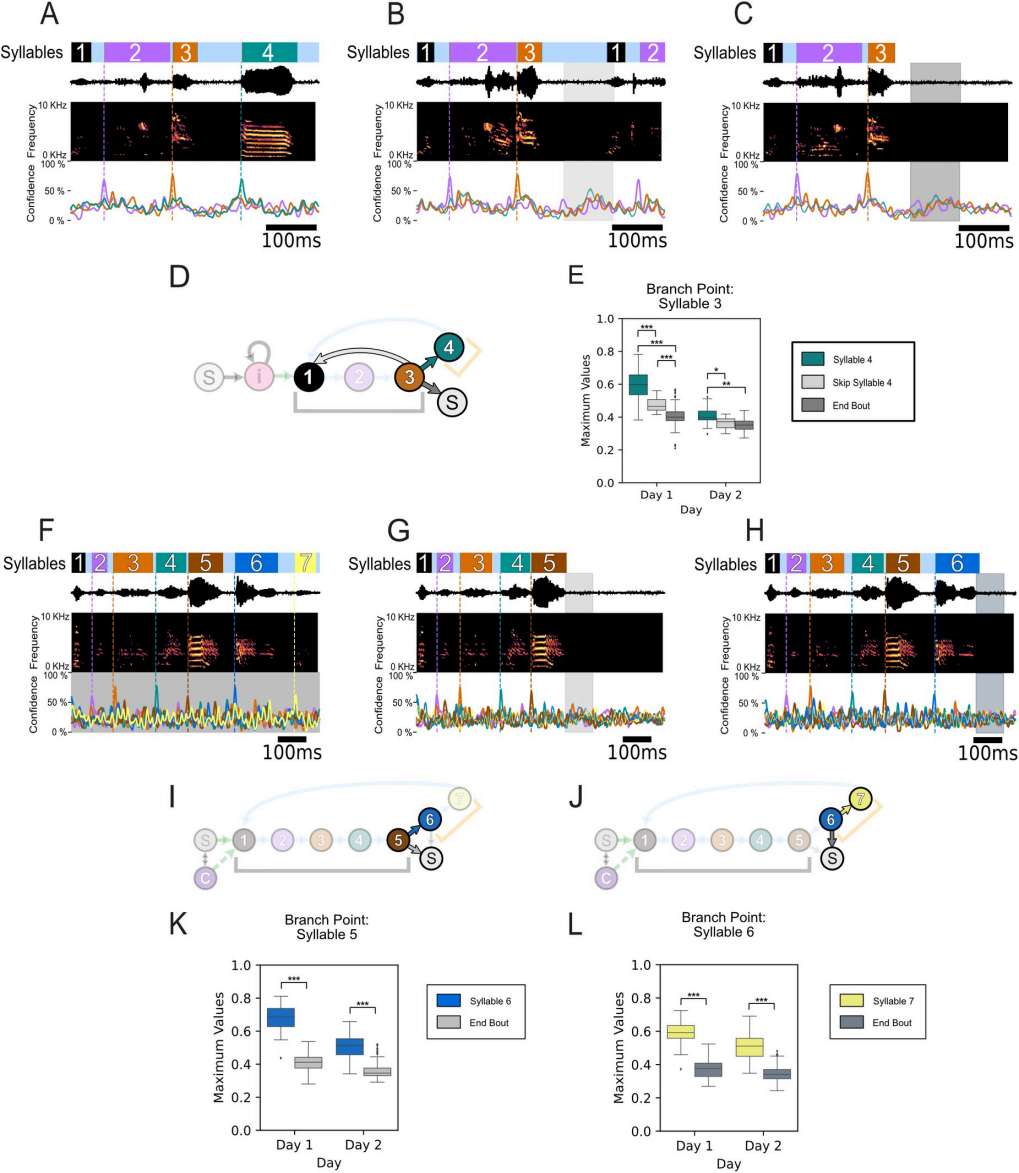
Onset prediction of non-deterministic syllables and decoding divergent behaviors using LFP features. (A) Example motif with all four syllables from the highest-yield day for subject z020. Annotated behavior (top) using the same color scheme as in Fig **3**, sound pressure waveform and the corresponding time-aligned spectrogram (middle), and the time-varying naïve confidence of the onset prediction (bottom) for each syllable in this example motif. (B) Example motif from the same high-yield day as (A) where the subject skips syllable 4 and continues the bout. The shaded lightgray time period is the time window that the behavior-based model would predict that the omitted syllable would occur. (C) Example motif from the same high-yield day as (A) where the subject ends the bout with syllable 3. The shaded darkgray time period is the time window that the behavior-based model would predict that the omitted syllable would occur. (D) State diagram of z020’s observed song structure with the three transition types highlighted; ‘Syllable 4’: syllable 3 to syllable 4 (teal) shown in (A); ‘Skip Syllable 4’: syllable 3 to syllable 1 (lightgray) shown in (B); ‘End Bout’: syllable 3 to silence (dark gray) shown in (C). Syllable colors are the same as in Fig **3**. (E) Boxplot of the difference in maximum confidence values between the three behavioral transition types shown in (A–C) using the same transition colors used in (D) across both high-yield days. (F) Example motif with all seven syllables from the highest yield day for subject z017. (G) Example motif from the same high yield day as (F) where the subject ends the bout with syllable 5. The shaded lightgray time period is the time window that the behavior-based model would predict the omitted syllable would occur. The prediction trace of the omitted syllables, both syllables 6 and 7, shows the mean across all folds, with shading indicating the standard deviation. (H) Example motif from the same high-yield day as (F) where the subject ends the bout with syllable 6. The shaded slategray time period is the time window that the behavior-based model would predict the omitted syllable would occur. The prediction trace of the omitted syllable, syllable 7 only, shows the mean across all folds with shading indicating the standard deviation across folds. (I) State diagram of z017’s observed song structure with the two transition types of the first behavioral branch point highlighted; ‘Syllable 6’: syllable 5 to syllable 6 (blue) shown in (F); ‘End Bout’: syllable 5 to silence (lightgray) shown in (G). Syllable colors are the same as in Fig **3**. (J) State diagram of z017’s observed song structure with the two transition types of the second behavioral branch point highlighted; ‘Syllable 6’: syllable 6 to syllable 7 (yellow) shown in (F); ‘End Bout’: syllable 6 to silence (slategray) shown in (G). Syllable colors are the same as in Fig **3**. (K) Boxplot of the difference in maximum confidence values between the two transition types, shown in (F) and (G), of the first behavioral branch point using the same transition colors used in (I) across both high-yield days. (L) Boxplot of the difference in maximum confidence values between the two transition types, shown in (F) and (G), of the second behavioral branch point using the same transition colors used in (J) across both high-yield days. Statistical significance was calculated using the one-sided Welch’s t-test (***p < .02 for all folds; **p < .05 for all folds; *p < .05 for four out of five folds).

As much of the sequence of the zebra finch song can be viewed as deterministic when only viewing the syllables of the stereotyped motif, it could be hypothesized that our onset prediction results are not decoding the song identity and are instead only finding the warped time of the syllable within the motif. To further evaluate this possibility, we analyzed the model’s performance during the non-deterministic syllables of the birds’ behaviors (i.e., their intra-motif notes). As shown in Fig 3B, Subject z020 has one behavioral branch point immediately after the third syllable, where it can either transition to its intra-motif note (syllable 4) or omit syllable 4. There are two different behavior options when the subject omits syllable 4; he can either immediately start another motif (skip syllable 4), or terminate the bout (end bout). Thus there are two types of omissions: (1) skip syllable 4 and (2) end bout. An example of the model’s single-trial performance for each case is shown in Fig 11A–11C. As syllable 4 does not occur during every motif, when testing the model’s performance on motifs that omit syllable 4 every template from the cross-validation folds is valid; because none of the motifs being tested can be in any of the training sets. Thus, we used each template to independently evaluate performance of each fold. Confidences that were derived from the same template across the different conditions were evaluated against one another. When comparing the distribution of the maximum confidence values during the time window the model would be required to predict this divergent behavior to occur, its confidence was statistically higher when the syllable occurred versus when it was omitted, grouping the two omission types together, across all five folds. These results were evaluated using the one-sided Welch’s t-test for each individual fold. In addition, repeating this test while treating the two syllable omission types as separate distributions yielded similar pair-wise results (Fig 11E). This same branch point analysis approach was used to evaluate the performance of subject z017, which has two intra-motif notes (Fig 3C) and two different branch points (Fig 11I and 11J). Fig 11F–11H show exemplar single-trial results that demonstrate performance that is robust to these non-deterministic behaviors. In addition, following the same branch point evaluation approach as previously described, we found when evaluating subject z020’s behavioral bifurcations the distribution of confidence values between motifs that contained the intra-motifs notes were significantly higher than when they were omitted (Fig 11K and 11L).

## Discussion

In the current study we demonstrate that LFP in zebra finch HVC carry significant information tied to vocal production. More specifically, the time-varying structure of this meso-scale signal, captured by both the power and phase of various LFP frequency bands, correlates with behaviorally-relevant timepoints during this complex, learned motor behavior. Our results builds upon previous studies in zebra finch HVC [39], by establishing that LFP features can be used to detect both when the bird will sing and the specific syllable that the bird will sing. Prior to that previous study and our work here, little was known about the relationship between LFP features and vocal production in birds. This limited the generalizability of the otherwise powerful birdsong model to other systems, including non-human primates and humans, where LFP and surface potentials are broadly used to measure the dynamics of cortical motor control [12,54,61,66]. In addition, we note that the bandwidth and features of the LFP signals investigated in this paper share similarities with LFP features tied to motor control in humans, non-human primates, and other mammals. For example, the power spectrum components most closely tied to song in finches (Figs 5 and S6–S8) match those documented in the human motor cortex during finger flexion [61]. We suggest that LFP recordings can serve as useful targets to further our understanding of songbird neurophysiology, and to more closely connect the powerful birdsong model to motor control studies in mammals, including non-human primates and humans.

A striking feature of the amplitude changes in LFP during song is the increase in higher frequencies that are within the band often referred to as “high gamma”. Although changes in this frequency band in another brain region in the song bird system, named Nif, have previously been reported [67], they were not found to be premotor related. We have shown the first preliminary evidence, to our knowledge, of premotor activity related amplitude changes in high gamma (S3 Fig). This is significant, as this band is often the feature of choice for many state-of-the-art neural decoding studies in humans and suggests designs for neurally driven speech prostheses [12,24]. Notably, these changes empirically appeared to correspond to introductory notes in addition to song syllable. Future work should evaluate if these amplitude changes also occur during calls, which are considerably less deterministic and more syntactically complex than the zebra finch motif.

A consistent feature observed in the LFP of HVC during singing is tight phase-locking to vocal behavior. In evaluating the nature of the observed song-aligned phase-locking; we proposed two hypotheses: (1) where the phase locking is related to the larger motif sequence and (2) in which the phase-locking can be attributed to the timing of each unique vocal unit (syllables). In simpler terms, we ask if the phase-locking is a process aligned to the start of the motif (hypothesis one) or if it is a process that aligns to the timing of each syllable (hypothesis two). In our examination of syllable level timing, we aligned time based upon the syllable onset time. This mirrors the time relationship between spiking activity and syllable onset [55,56]. Further examination would be required to examine the progression of phase with respect to the temporal structure within each syllable.

When directly testing these hypotheses, the second hypothesis was supported by statistical analyses of the data. However, it is important to note that while there is noticeable non-deterministic jitter in the brief gaps of silence between the syllables that make up the motif, this may not be sufficient to completely disprove hypothesis one. On the other hand, there are additional notes, beyond the stereotyped sequences of the motif, that occur non-deterministically between motifs. These notes are referred to as intra-motif notes or “connectors” [60] and introductory notes for whose timing before the motif is variable. Examination of the phase polar-plots of individual syllables would either show high selectivity of phase preference for all unique vocalizations if our second hypothesis is correct, or low levels of phase-locking for motif syllables and a random distribution about the polar axis (null) for the intra-motif notes and introductory notes if our first hypothesis is correct. As shown in Figs 7 and 8, each syllable has phase preference that is stable across every specific vocalization type, including intra-motif notes (z017 & z020) (S11–S13 Figs) and introductory notes (z020 & z007) (Figs 7 and S10). This finding demonstrates that precise phase preference is behaviorally relevant to both learned and non-learned vocalizations. We argue that the long template phase traces in lower frequencies that precede and persist during song production are a byproduct of the zebra finches’ stereotyped song structure. This structure is composed of encoding at a smaller time scale centered on each individual syllable during song production. Similar phase preference to motor onset has been found in both human and non-human motor cortex [68].

The results of the normalized sustained Z statistic of inter-trial phase coherence suggest that there are differences in magnitude of coherence peaks both between and within birds. This is likely due to significant variation in signal statistics. This variation likely has many sources, including precise electrode placement and varying signal-to-noise ratios across days. Within days, the peaks in the Z-statistic denote consistency in phase-locking. Although the magnitude of these peaks varied, the frequency bands that contained them were empirically similar to those described previously in mammalian literature. These results, the existing literature, and our empirical inspection of the narrowbands, as shown in S4 and S5 Figs, informed our narrowband feature selection.

Previous work in zebra finch by Markowitz et al. demonstrated that spiking of both excitatory and inhibitory neurons were locked to the phase of the 25–35 Hz band [37,39]. Admittedly, this previously identified phase-locking, combined with the behavior-locked manner of local spiking activity to song behavior, gives further credence to some of the phase-locking we observed. However, it is important to note that local neural spiking activity and LFP are not deterministically linked. LFP reflects the postsynaptic inputs to the local neuronal population and transmembrane currents in the recorded region, which is of course different from the spiking in that region, which reflects neuronal outputs [69]. Additionally, our work establishes that other frequency bands beside the 25–35 Hz band are phase locked to the behavior. More importantly, we find that these frequencies, and the 25–35 Hz band, are also locked to behaviors beyond the syllables of the motif, namely intra-motif notes and introductory notes. These LFP features predict the presence or absence of intra-motif notes, as well as their onset time (Fig 11). These findings suggest that additional vocalizations in the zebra finch repertoire can be used to study motor-vocal control. These behaviors are significantly less deterministic than the syllables of the motif. The stereotyped structure of the zebra finch’s song differs from the more complex structure of human speech. Broadening the vocal behaviors that can be used in physiological and neural engineering motivated studies can help mitigate this weakness in the zebra finch model. In addition, the methods and insights learned from these less deterministic vocalizations can be applied to songbirds with more complex song structures such as starlings, canaries, and Bengalese finches. Collectively these songbird models provide an opportunity to investigate vocal-motor encoding at varying levels of behavioral complexity.

Naturalistic motor behavior is intrinsically difficult to study due to its high degree of variability. Zebra finch represents a desirable model species because they mitigate this difficulty. Their song allows for repeated segments of the same sequence to be produced with two almost opposing characteristics: near perfect precision of each utterance and the natural irregularities that exist in all of nature. This provides a dataset that has both high repetitions of each syllable class and a non-deterministic structure beyond the motif level that facilitates detailed analyses. This model is a unique testbed for approaches to predict the onset of vocalizations in other animals, particularly humans. The onset prediction results in which each syllable beyond the first was predicted from LFP features, described in Fig 10, were computed with little-to-no parameter optimization. Better performance could be achieved with more elegant approaches with minimal increases in computational complexity. Furthermore, all computations were conducted with neural activity preceding syllable onset, suggesting that these features could be leveraged to predict syllable onset with low latency.

At present the state of the art for human speech decoding intended for neural prosthesis applications are non-causal, utilizing neural signals before and after the intended vocalization to synthesize sounds similar to those of the intended speech [12,24,70]. While promising, the non-causal nature of these algorithms introduces significant latency [70] and ultimately limits the quality of interaction with such a prosthesis. Even further, the limits on duration over which neural signals can be studied impedes the pace at which these methods can be established and translated into clinical application. This is where the songbird model can contribute to the development of neural prostheses: providing an animal model for proof-of-concept system development in which closed-loop interaction with prosthesis designs can be rigorously studied. Systems derived and refined in this model system could then be when translated to the human clinical setting, in which such rigor and repeatability are more challenging to achieve. These features and this approach provide a starting point for further analyses that look to zebra finch and other songbirds as more than a model for vocal learning and sequence generation, but also as a model of vocal prediction and neural prosthesis development.

## Methods

### Ethics statement

The care of animals in this study was carried out in accordance with the University of California IACUC protocol number S15027.

### Summary

We implanted male zebra finch with laminar electrodes and simultaneously recorded their vocal behavior and neural activity (Fig 1). Through a series of steps, explained in detail in the Annotation and Alignment of Behavioral Data section, we found segments of the recording that contained songs and hand-annotated them using Praat (Fig 2). These behavioral labels were used to analyze the neural activity in relation to specific classes of behavior to determine what frequencies, if any, correlated to the behavior (Figs 4–7). Finally, the resulting frequencies were used to classify and predict behavior onset to clarify their relationship to the vocalizations (Figs 9–11).

### Subjects

All procedures were approved by the Institutional Animal Care and Use Committee of the University of California (protocol number S15027). Electrophysiology data were collected from three adult male zebra finches. Birds were individually housed for the entire duration of the experiment and kept on a 14-hour light-dark cycle (Fig 1A). Each day had one session that lasted multiple hours: recording days were unstructured, as they depended on the subject’s willingness to sing (Fig 2A). All available days were analyzed; however, the two highest motif yielding days, hereafter referred to as *high-yield days*, were reported and used for statistical analysis. The full duration of chronic recordings ranged from 5 to 19 days. The birds were not used in any other experiments.

### Electrophysiology and audio recording

Electrophysiological recordings were gathered from 3 subjects, in which two were implanted with 16-channel probes and one with a 32-channel probe (Fig 1A). We used 4-shank, 16/32 site Si-Probes (Neuronexus), which were PEDOT-coated in-house (https://www.protocols.io/view/EDOT-PSS-c2syed). The probes were mounted on a custom designed prinable microdrive [71] and implanted targeting nucleus HVC (Fig 1A and 1B). Audio (vocalizations) was recorded through a microphone (Earthworks M30) connected to a preamplifier (ART Tube MP) and registered temporally to ongoing neural activity (Fig 1)]. Extracellular voltage waveforms and pre-amplified audio were amplified and digitized at 30 kHz using an intan RHD2000 acquisition system, Open Ephys and custom software (Fig 1D) [72].

### Electrode implantation procedure

Animals were anesthetized with a gaseous mixture of Isoflurane/oxygen (1–2.5%, 0.7 lpm) and placed in a stereotaxic frame. Analgesia was provided by means of a 2mg/kg dose of carprofen (Rimadyl) administered I.M. The scalp was partially removed and the upper layer of the skull over the y-sinus was uncovered. The probe was attached to the shaft of a microdrive of our design (https://github.com/singingfinch/bernardo/tree/master/hardware/printable_microdrive), which was printed in-house using a b9 Creator printer and the BR-9 resin. A craniotomy site was open 2400 μm medial to the y-sinus (right/left hemispheres). The dura was removed and the electrode array was lowered to a 300–500μm depth. The opening was then covered with artificial dura (DOWSIL 3–4680 Silicone Gel Kit) and the microdrive was cemented to the skull using dental cement (C&B Metabond). A reference wire was made with a 0.5 mm segment of platinum-iridium wire (0.002”) soldered to a silver wire lead and inserted between the dura and the skull through a craniotomy roughly 3mm medial (contralateral to the hemisphere where the electrode was inserted) and 1.5 mm anterior to the y-sinus. The reference electrode was cemented to the skull and the silver lead was soldered to the ref and gnd leads of the neuronexus probe. The craniotomy, the electrode, and the Microdrive were then covered with a chamber designed and 3D printed in-house, and which was subsequently cemented to the skull. The skin incision was sutured and adhered to the chamber with adhesive. The mass of the probe, Microdrive, and protective chamber were measured to be 1.2–1.4g. Upon the finches returning to a single-housing cage, a weight reliever mechanism was attached using the end of a thin nylon wire that was attached to an ad-hoc pin in the chamber; the other end routed through a set of pulleys and attached to a counterweight mass of ~1g [72].

### Analysis of electrophysiology data

Extracellular voltage traces were multiplexed and digitized at 30kHz on the headstage, and stored for offline analysis. **Offline:** Data were then low-passed filtered at 400 Hz using a Hamming finite impulse response (FIR) filter and downsampled to 1 kHz. The group delay introduced by the filter is compensated by introducing a temporal shift to the filter output [73].

### Annotation and alignment of behavioral data

The song of an adult male zebra finch can be partitioned and labeled in multiple ways. However, the most fundamental characteristic of their song that is agreed upon is their stereotyped motif. The motif is a consistent sequence of sounds interleaved with silent periods that is learned from a tutor while they are young [10,35,36]. Song motifs are also grouped into larger structures called bouts, which consist of multiple repetitions of motifs [10,35,60]. Depending on the definition used, bouts can include a short, repeated vocalization that typically precedes the first motif of the bout. For the purpose of the analysis done, the start of the bout is explicitly defined to be the start of the first motif in that bout. This is due to the focus of understanding the correlation between LFP and learned motor sequences. Beyond the syllables of the motif male zebra finch may have an additional syllable or sequence of syllables that they will optionally insert between successive repetitions of motifs. This is called a “connector” [60] or intra-motif note. In our recordings, both z017 and z020 had intra-motif notes. As z007 did not have any intra-motif notes, and therefore had a more stereotyped song, this bird will be used for all of the empirical figures shown.

A custom template matching algorithm written in Python was used to find potential instances of vocal activity (using an exemplar motif as the template); these instances were then curated manually to rule out false positives [72]. The curated motif start times were grouped into larger time segments that ensured that the gap between each motif was no greater than 20 seconds (Fig 2B). These chunks of time are subsequently referred to as vocally active periods (VAP). These VAPs were then manually labeled using the Praat speech analysis software [74]. Vocal events were segmented by hand (Fig 2C) and labeled based on identity. There is a unique label for each individual syllable and introductory note (if the bird demonstrated one). All other male vocalizations were grouped together and labeled as a call. Calls are short, simple vocalizations that are produced by both sexes and which mostly do not have learned components [36]. There were also two labels for silence in these VAPs: one for the gaps between song syllables, and another for silence not during song in the VAP.

To leverage these behavioral annotations for contextual analysis of their synchronously recorded neural activity, custom Python software was developed [BirdSongToolbox]. This software added additional labels to the hand labels based on their sequence and context within the larger bout structure (e.g., first or last motif in bout). Labeled vocalization segmentation times were adjusted to the proper sampling rate for the downsampled neural data using this same software. Finally, the software used these additional contextual labels to select particular event times to align both vocal and neural data that fit certain criteria matching the particular vocalization of interest.

### Time series power spectrum

Spectrograms were calculated by first filtering the data with 100 partially overlapping narrow frequency bands that were equal width in log-space. These filtered time series were Hilbert-transformed to extract the analytic amplitude at each time point. Each narrowband amplitude time series was then normalized by the mean of that frequency band over the entire duration of its VAP.

### Cross trial z-scored ratings of averaged spectrograms

The cross trial z-scored ratings of averaged spectrograms was calculated by first mean centering each frequency by its trial-wise mean

$$\hat{s}(f,t,n)=s(f,t,n)-\frac{1}{T*N}\sum_{m=1}^{N}\sum_{\tau =1}^{t}s(f,\tau ,m)$$ (1)

Where s(f,t,n) represents every time, t, frequency, f, sample for each of the trials, n, spectrogram which were averaged in the manner used to create Fig 4. The mean of each time-frequency sample was then divided by the standard deviation of its time-frequency sample across trials.


$$S(f,t)=\frac{\frac{1}{N}\sum_{n=1}^{N}\hat{s}(f,t,n)}{{stdev}_{n}(\hat{s}(f,t,n))}$$ (2)

This approach calculates a z-score of each time-frequency mean to evaluate how well it represents that result across all trials. The closer to zero the value the more accurately it reflects the spectral change across all renditions of the motif. The greater in absolute magnitude the value the more it is above or below the norm across trials. A value of +/-2 is a approximately to within a 95% confidence interval for all trials values.

### Principal spectral decomposition

Following Human ECoG studies [61], a principal component method was applied to find consistent changes in LFP spectrum related to motor-vocal behavior. This method required calculation of the power spectral density (PSD) for windows of time during either vocal activity or inactivity. Although the behavioral changes in birdsong are on a smaller timescale (20–100 ms) than human limb movements, to keep methods consistent between studies the same window size of 1 second was used for the trial length, *τ_q_*.

PSDs of vocally active trials centered on song activity, which included introductory notes and calls, were analyzed alongside trials of neural activity centered within larger periods of vocal inactivity longer than 2 seconds in duration. All PSDs were calculated using the multitaper method with the time_frequency.psd_array_multitaper function from the MNE-Python software package [73]. The frequency range was set from 0 to 200 Hz with 14 orthogonal slepian tapers. Each PSD, *P*(*f, τ_q_*), was individually normalized using two steps: each spectral sample was elementwise divided by the mean across the ensemble, at each frequency, and then the log was taken. This centers the data around the log of the mean spectrum.


$$\bar{P}(f,\tau_{q})=\ln\left(P(f,\tau_{q})\right)-\ln\left(\frac{1}{N_{q}}\sum_{p=1}^{N_{q}}P(f,\tau_{p})\right)$$ (3)

The label *q* refers to the times centered within periods of high vocal activity and vocal inactivity (silence). The number of instances, or trials, per class were balanced to be equal; their combined total number of PSDs is denoted by *N_q_*. The order of the trials was explicitly ignored, meaning that they together represent a balanced ensemble of *N_q_* independent measurements of the power spectrum during the two conditions. The covariance matrix $C(f,\tilde{f})$ between frequencies of these normalized PSDs were calculated:

$$C(f,\tilde{f})=\sum_{\tau_{q}}\tilde{P}(f,\tau_{q})\tilde{P}(\tilde{f},\tau_{q})$$ (4)

The covariance measure is centered with respect to the log of the mean spectrum. The eigenvalues, *λ_k_*, and eigenvectors, ${\vec{e}}_{k}$, of this matrix elucidate common features during song production. These eigenvectors, ${\vec{e}}_{k}$, are referred to as “principal spectral components” (PSCs) and they have been described in prior literature to reveal which frequencies vary together [61].

### Cosine similarity

Cosine similarity is a measure of similarity between two non-zero vectors of an inner product space. For the purpose of comparing the principal spectral components, cosine similarity evaluates the orientation, not magnitude, of two vectors in relation to one another. To calculate this, one takes the inner product of two unit vectors, or two vectors that have each been normalized to have a length Of 1.


Similarity=Α·Β=‖Α‖‖Β‖cosθ (5)

To calculate the cosine similarity for the PSCs we must take the dot product between two unit vectors. As the PSCs are all eigenvectors calculated using PCA they are already unit vectors and their sign can thus be flipped without altering their information. A template of each PSC is calculated by taking the mean of the PSCs across all good channels after all of their signs have been aligned. This template PSC is then normalized to have a length of one by dividing it by its norm. The cosine similarity matrix is then calculated by taking the dot product of these two unit vectors. This is expressed below with the template PSC represented as T and the PSC symbolized by P from a selected channel, c.


$$Similarity=P_{c}\cdot \frac{Τ}{\|Τ\|}$$ (6)

### Feature extraction

Feature extraction from the preprocessed data comprised a series of steps that involved first Common Average Referencing and then narrow band-pass filtering. Common Average Referencing has been shown to minimize or remove the contributions of correlated noise sources such as 60 Hz noise and intermittent noise sources such as movement artifacts [75]. We used the Hilbert transform to compute the analytic signal. To extract oscillatory power, we used the absolute value of the analytical signal; this effectively removed all phase-related information. To extract oscillatory phase, we used the instantaneous angle of the complex argument of the analytical signal; this was further smoothed using a sine function. This effectively removed all information related to power from the signal.

These signals, whether Hilbert-transformed or not, were then sub-selected at times relative to the label onset for a syllable using two parameters: bin width and offset. The bin width is the number of samples used prior to the offset, and the offset is the number of samples, or milliseconds, prior to the labeled start time to a specific syllable. All combinations of these hyperparameters used offset that were prior to the start of the vocal behavior.

#### Band templates and Pearson correlation features

Using the optimized bin width and offset for a particular frequency band, we calculated a template for that band by taking the mean of the LFP traces of the training set of a specific behavior class. This template represents the average LFP activity prior to and during the production of a particular vocal behavior. This template was then used to extract features from a narrow-band LFP trace by taking the Pearson correlation of the template from a segment of neural activity of the same length in samples. This correlation value is set between -1 and 1. For the behavioral classification results, a segment of the corresponding LFP frequency band that is set by the optimized bin width and offset is used as the feature. For the onset detection analysis, the Pearson correlation for each sliding segment equal in length to the optimal bin width is used to detect behavior that corresponds to its optimal offset.

### Computation and statistical testing of phase locking across renditions

We computed the inter-trial phase coherence (ITPC) to assess trial-to-trial synchronization of LFP activity with respect to time-locked syllables within the motif. The ITPC is a measure of the phase synchronization of neural activity in a given frequency, calculated relative to critical event times that are repeated over an experimental paradigm. To calculate the ITPC across trials, for each channel, data were first filtered in 100 partially overlapping narrow frequency bands that were equal width in log-space. These filtered time series were then Hilbert-transformed to extract the analytic phase at each time point. Then, at each time point, for each channel and frequency band, we calculated the phase-consistency across trials to estimate the ITPC. Here, ‘trials’ were defined as the start or end of a specific type of vocal event, which could be further refined by its context within the bout (e.g., first or last in bout). Once all instances of the event of interest were selected, a window of time centered on each was implemented. The ITPC for each frequency and time sample pair must be calculated independently. The equation for calculating the ITPC is described as below, for n trials, if, F_k_(f, t) is the spectral estimate of trial k at frequency f and time sample t

$$ITPC\;(f,t)=\frac{1}{n}\sum_{k=1}^{n}\frac{F_{k}(f,t)}{\left|F_{k}(f,t)\right|}$$ (7)

where |*x*| represents the complex norm of *x* [76]. The spectral estimate, F_k_(f, t), is the instantaneous phase of frequency f at time sample t. This was repeated for all narrow-band frequencies and time samples within the event window.

The ITPC value, or resultant vector length, scales from 0 to 1, with 0 being the null hypothesis where phases are uniformly distributed about the polar axis and 1 being perfect synchrony. To determine the significance of this vector length, and to determine if it could have been randomly sampled from a uniform distribution by chance, p-values were calculated for each frequency and time sample. The mean resultant vector of the instantaneous phase across trials for a specific time sample, and its corresponding bootstrapped p-value, were calculated using the pycircstats toolbox. To enable visualization of the results for comparing ITPC values while accounting for all of the frequency and time sample combinations, the Rayleigh Z statistic was calculated.

#### Rayleigh statistic

The ITPC over the time length of a syllable or motif is evaluated in terms of the Rayleigh Z statistic and is defined as $Z=\frac{R^{2}}{n}$where R is the Rayleigh’s R, ***R = nr*** where r is the resultant vector length, and n is the number of trials. P-values were estimated using the following equation [77–80]:

$$P=exp[\sqrt{1+4n+4(n^{2}-R^{2})-(1+2n)}]$$ (8)

#### Normalized sustained Rayleigh Z statistic

To determine which, if any, frequency bands could potentially be used as a feature to decode vocal behavior, a measure of how consistently significant a band stayed phase-locked was created. As we don’t expect the Rayleigh Z statistic across both channels and frequencies to be exactly the same, we wanted to understand the relative significance across birds and channels. The measure ranks how consistently phase-locked across time a frequency was during song production overall for one recording session. The mean Rayleigh Z-statistic of samples over the course of one oscillation for a set frequency normalized by the maximum Z-statistic value for the entire time period analyzed was used, and will be referred to as the normalized sustained Z statistic. This analysis informed the frequency bands used for the classification and onset detection analysis.

### Linear classifier

To classify behavior using the LFP features, a linear discriminant analysis (LDA) model using singular value decomposition (SVD) was trained using the scikit-learn toolbox [81] in Python. This classifier is tasked to correctly classify examples of all vocalizations during song, both motif syllables and intra-motif notes, in addition to introductory notes and periods of silence. As the classifier must learn how to distinguish between both vocal and non-vocal events, these events are collectively referred to as classes. All priors for each class were set equal, and all classes were balanced in the datasets used for classification analyses. Detailed information regarding the number of instances in each class can be found in S3–S5 Tables. No shrinkage or regularization penalties were used; however, the SVD optimizer was applied to regularize the covariance matrices and avoid ill-conditioning. Results were validated using 5-fold cross-validation. Templates for feature extraction were created by taking the mean across the training set. These templates were used to extract features from both the training set and the testing set. All frequencies were trained and tested independently of one another.

#### Channel-adding analysis

Channel-adding curves were calculated using a bootstrap approach to determine how many channels were needed until additive information saturation. The channel-adding curves were calculated by first training and testing a classifier with the neural features of only one channel, using the steps described previously, then repeating this analysis after adding the features of a another randomly selected channel. This repeated training and testing after adding a random channel is considered complete once a classifier that uses the features from all available good recording channels is evaluated. This channel-adding analysis was repeated 5,000 times, changing the order in which each channel was included, with 5-fold cross validation. The order in which channels were added over the 5,000 repetitions was maintained across the folds to enable fair calculation of their validated mean. These are subsequently used to calculate the mean and standard deviation across repetitions.

### Syllable onset detection

In order to analyze the temporal relationship between LFP and song production, we used a template-matching approach to determine whether LFP can predict syllable onset. Each syllable of the motif—excluding the first—had every labeled instance recorded in the same day split into training and testing sets using a 5-fold stratified split (80% training set and 20% testing) (S3–S5 Tables). Both the template and the stereotyped onset of the syllable were calculated from the training set. Templates are the mean of the LFP traces of the training set. The stereotyped onset is the average time the syllable occurs in the training set with respect to the first syllable of the motif it occurred in. These templates were then used to compute the Pearson correlation across time for each of the motifs that contain the syllables of the test set, maintaining the temporal relationship of the optimal offset and the bin width for its respective frequency.

The prediction confidence of a single frequency band was calculated by first thresholding the Pearson correlation values at zero, and taking the average of the resultant time series across channels. The maximum confidence within a 100-millisecond window centered on the behavior-derived stereotyped onset time is then used as the frequency’s prediction for that instance of the syllable. We followed the same steps as previously stated in order to produce the results of a predictor that uses all of the frequencies; only the prediction confidence across all frequencies and channels is used. The prediction of the syllable onset using the birds’ stereotyped behavior was determined by adding the stereotyped onset time, calculated from the training set, to the actual start of the first syllable for the motif the syllable occurred in. It is important to note two things with this approach: (1) neither approach receives information on the actual start time of the syllable in its respective motif and (2) this 100-millisecond window is significantly larger than the natural variability of syllable onsets, providing the neural-based predictor a more difficult task than the baseline stereotyped comparison. In addition, for later syllables of the motif there are a few instances where the syllable occurs after the 100-millisecond window; meaning that the neural detector will be forced to predict the syllable in a window where it hasn’t yet occurred. Due to this the neural decoder has a larger possible maximum prediction error than the behaviorally based predictor. Both predictions were then normalized by taking the difference between the predicted time and the actual labeled start time of the syllable within the same motif.

Statistical significance of the results was calculated with a one-sided Wilcoxon signed-rank test using the difference between the relative predictions of the stereotyped behavioral model and the neural features models. Two null hypotheses were tested, first that there was no difference between the stereotyped behavior and the prediction using neural features, and the second being that the predictor using the neural features was closer to the actual labeled onset time. The second null hypothesis requires that the LFP based predictor must outperform the behavior-based predictor. Results must pass both tests with a p-value less than .05 to be considered significant. The Benjamini-Hochberg procedure was used to control the False Discovery Rate to account for the multiple comparisons done. The procedure was implemented for each day treating the results of each individual frequency and the predictor which uses all frequencies as separate results (n = 6, q = .05).

## Supporting information

S1 Table Overall Description of Subject Data. (XLSX)

S2 Table Detailed Description of available labeled data. (XLSX)

S3 Table Behavior of Subject z007 during High Yield Days. (XLSX)

S4 Table Behavior of Subject z020 during High Yield Days. (XLSX)

S5 Table Behavior of Subject z017 during High Yield Days. (XLSX)

S6 Table Characteristic Increases in Power in Narrowband Frequencies during Song Across Channels. The percentage of channels whose distribution of power values were greater during periods of vocal activity than during silence. To determine statistical significance between the two distributions the one-sided z-test was used with each frequency and channel pair. The percentages shown are the number of good channels that still were significant after using the Benjamini-Hochberg False Discovery Rate (p<0.05 and q<0.05).
(XLSX)

S7 Table Characteristic Decrease in Power in Narrowband Frequencies during Song Across Channels. The percentage of channels whose distribution of power values were smaller during periods of vocal activity than during silence. To determine statistical significance between the two distributions, the one-sided z-test was used with each frequency and channel pair. The percentages shown are the number of good channels that still were significant after using the Benjamini-Hochberg False Discovery Rate (p<0.05 and q<0.05).
(XLSX)

S8 Table Characteristic Decrease in High Gamma Power After Bout Termination Across Channels. The percentage of channels whose distribution of power values for 80–200 Hz, or High Gamma, were smaller during the 100ms silent period after the bout ends than the 100ms period prior to the end of the bout. To determine statistical significance between the two distributions, the one-sided Welch’s t-test was used for each channel. The percentages shown are the number of good channels that still were significant after using the Benjamini-Hochberg False Discovery Rate (p<0.05 and q<0.05).
(XLSX)

S9 Table Sensitivity of Channels as Assessed by Permutation Test. A sensitivity index is assessed between the vocally active period and inactive period for the first 9 PSCs calculated for each channel. The number (and percentage) of channels with a sensitivity index significantly greater than the null distribution, as estimated by a permutation analysis, and after application of the Benjamini-Hochberg False Discovery Rate (p<0.05 and 1<0.05) was assessed.
(XLSX)

S10 Table. Bin Width Hyperparameter Search Results (XLSX)

S11 Table. Offset Hyperparameter Search Results (XLSX)

S1 Fig Song-correlated modulation of LFP power across subjects and days. Averaged spectrotemporal power activity (see Methods) aligned to the start of the first motif in the bout, left, and the last motif in the bout, right, for the additional recording days that are not plotted in Fig 4. Shown above all results is a behavioral raster showing the time course of the behavior being averaged. (A) The averaged results for the second highest-yielding day, designated Day 1, for z007 (n = 25 Bouts). The other subjects’ results are show as follows; (B) z020’s first high yield day (n = 29 Bouts), (C) z020’s second high-yield day (n = 25 Bouts), (D) z017’s first high-yield day (n = 27 Bouts), and (E) z017’s second high-yield day (n = 21 Bouts). As z017 would end its bout on either syllable ‘5’ or ‘6’, the end of the bout was aligned to syllable ‘5’. No dynamic-time warping was used. To ensure the start and end of the bout are unique time periods, only bouts with more than one motif in duration were used. Behaviorally inconsistent bouts were excluded for clarity of visualization; however, results are consistent when including them in the analysis.
(TIF)

S2 Fig Evaluation of consistency across trials of song correlated modulation of LFP power across subjects and days. Cross trial z-scored ratings of average spectrograms (see Methods) aligned to the start of the first motif in the bout, left, and the last motif in the bout, right, for each recording day. The z-score metric normalizes the modulation in LFP power at each frequency and time point by its standard deviation across trials. Thus the metric quantifies the number of standard deviations, as measured across trials, between the mean LFP power at each timepoint/frequency and the mean LFP power across the VAP. Shown above all results is a behavioral raster showing the time course of the behavior being evaluated. (A) The z-scored results for the first high yield day, designated Day 1, for z007 (n = 25 Bouts) (B) The z-scored results for the second high-yield day, designated Day 2, for z007 (n = 27 Bouts). The other subjects’ results are shown as follows; (C) z020’s first high yield day (n = 29 Bouts), (D) z020’s second high-yield day (n = 25 Bouts), (E) z017’s first high-yield day (n = 27 Bouts), and (F) z017’s second high-yield day (n = 21 Bouts). As z017 would end its bout on either syllable ‘5’ or ‘6’, the end of the bout was aligned to syllable ‘5’. No dynamic-time warping was used. To ensure the start and end of the bout are unique time periods, only bouts with more than one motif in duration were used. Behaviorally inconsistent bouts were excluded for clarity of visualization; however, results are consistent when including them in the analysis.
(TIF)

S3 Fig Song correlated changes in power of 50–200 Hz band across days and subjects. Z-scored changes in power of the 50–200 Hz band aligned to the start of the first motif in the bout, left, and the last motif in the bout, right, for each high-yield recording day. Black traces in each subpanel show the mean, and the green shading is the standard deviation. The end of the bout, and the subsequent drop in power are annotated by a black arrow. Above all results is a behavioral raster showing the time course of the behavior being averaged. (A) The results of the first-high yield day of z007 (n = 27 Bouts). (B) The results for the second high-yield day for z007 (n = 25 Bouts). The other subjects results are shown as follows; (C) z020’s first high-yield day (n = 29 Bouts), (D) z020’s second high-yield day (n = 25 Bouts), (E) z017’s first high yield day (n = 27 Bouts), and (F) z017’s second high-yield day (n = 21 Bouts). As z017 ends its bout on either syllable ‘5’ or ‘6’, both types of bout endings are shown separately. No dynamic-time warping is used. To ensure that the start and end of the bout are unique time periods, only bouts with more than one motif in duration are used. Behaviorally inconsistent bouts are excluded for clarity of visualization; however, results are consistent when including them in the analysis.
(TIF)

S4 Fig Stereotyped song correlated rhythmic changes in LFP across days and subjects (single trial). Single-trial z-scored LFP traces of four narrowband frequency bands aligned to the start of the first motif in the bout, left, and the last motif in the bout, right, for each high-yield recording day. Each trace is colored its respective narrowband frequency. Shown above all results are behavioral rasters showing the time course of the behaviors being shown. The black line in each row below the behavior shows the time point the trials are aligned to, and the blue line denotes the time prior to (left) or after (right) one full cycle of the highest frequency in the narrowband frequency. (A) The results of the first high-yield Day of z007 (n = 27 Bouts) (B) The results for the second high-yield day for z007 (n = 25 Bouts). The other subjects’ results are shown as follows; (C) z020’s first high-yield day (n = 29 Bouts), (D) z020’s second high-yield day (n = 25 Bouts), (E) z017’s first high-yield day (n = 27 Bouts), and (F) z017’s second high-yield day (n = 21 Bouts). As z017 would end its bout on either syllable ‘5’ or ‘6’, the end of the bout was aligned to syllable ‘5’. No dynamic-time warping was used. To ensure that the start and end of the bout are unique time periods, only bouts with more than one motif in duration were used. Behaviorally inconsistent bouts were excluded for clarity of visualization; however, results are consistent when including them in the analysis.
(TIF)

S5 Fig Stereotyped song correlated rhythmic changes in LFP across days and subjects (mean and standard deviation). The mean and standard deviation of the z-scored LFP traces of four narrowband frequency bands aligned to the start of the first motif in the bout, left, and the last motif in the bout, right, for each high-yield recording day. Each row is colored its respective narrowband frequency. Above all results is a behavioral raster showing the time course of the behaviors being shown. The black line in each row below the behavior shows the time point that the trials are aligned to, and the blue line denotes the time prior to (left) or after (right) one full cycle of the highest frequency in the narrowband frequency. (A) The results of the first high yield day of z007 (n = 27 Bouts) (B) The results for the second high-yield day for z007 (n = 25 Bouts). The other subjects’ results are shown as follows; (C) z020’s first high-yield day (n = 29 Bouts), (D) z020’s second high-yield day (n = 25 Bouts), (E) z017’s first high-yield day (n = 27 Bouts), and (F) z017’s second high-yield day (n = 21 Bouts). As z017 would end its bout on either syllable ‘5’ or ‘6’, the end of the bout was aligned to syllable ‘5’. No dynamic-time warping was used. To ensure that the start and end of the bout are unique time periods, only bouts with more than one motif in duration were used. Behaviorally inconsistent bouts were excluded for clarity of visualization, however results are consistent when including them in the analysis.
(TIF)

S6 Fig Summary of principle spectral component analysis. (A) The 1st Principle spectral components for every channel (light grey) with the mean across channels in Blue for all High Yield Days for each subject. (B) The 2nd Principle spectral components for every channel (light grey) with the mean across channels (golden-yellow) for all High Yield Days for each subject. (C) The 3rd Principle spectral components for every channel (light grey) with the mean across channels (burgundy) for all High Yield Days for each subject.
(TIF)

S7 Fig Similarity of principal spectral components across channels, days and subjects. Boxplot of the distribution of cosine similarity metric values between a template, which is created by taking the mean across the sign-aligned PSC of all channels for a specific PSC. The cosine similarity matrix ranges from 1 and -1, however, the absolute value of the metric is shown (see Methods). (A) The cosine similarity of each channel’s PSC to the same recording day’s template PSC for both high-yield days for subject z007. (B) The cosine similarity of each channel’s PSC to the same recording day’s template PSC for both high-yield days for subject z020. (C) The cosine similarity of each channel’s PSC to the same recording day’s template PSC for both high-yield days for subject z017. (D) The cosine similarity of each channel’s PSC from the second high-yield day with the template of the PSC from the first high-yield day. (E) All templates for PSC 1 compared either with the PSC 1 for the other two birds, same, or the PSC for one of the other PSCs for the other two birds, other. (F) All templates for PSC 2 compared either with the PSC 2 for the other two birds, same, or the PSC for one of the other PSCs for the other two birds, other. (E) All templates for PSC 3 compared either with the PSC3 for the other two birds, same, or the PSC for one of the other PSCs for the other two birds, other.
(TIF)

S8 Fig Separability of Active vs. Inactive trials using principal spectral components. (A) Box plots of the sensitivity indexes (d’) for the separation of the Active and Inactive trials for all channels using the first 9 PSCs. The plots show the distribution of values for both high yield days for all three subjects. (B) Blox plots of the p-values of the sensitivity indexes shown in (A) when tested against a bootstrapped shuffle control (N = 20,000 Shuffles).
(TIF)

S9 Fig Inter-trial phase coherence of LFP phase during production of learned sequences across subjects and days. ITPC of LFP aligned to the start of the first motif in the bout, left, and the last motif in the bout, right, for the additional recording days that are not plotted in Fig **6**. Shown above all results is a behavioral raster showing the time course of the behavior being averaged. (A) The averaged results for the second highest-yielding day, designated Day 1, for z007 (n = 25 bouts). The other subjects’ results are show as follows; (B) z020’s first high-yield day (n = 29 bouts), (C) z020’s second high-yield day (n = 25 bouts), (D) z017’s first high-yield day (n = 27 bouts), and (E) z017’s second high-yield day (n = 21 bouts). As z017 would end its bout on either syllable ‘5’ or ‘6’, the end of the bout was aligned to syllable ‘5’. No dynamic-time warping was used. To ensure that the start and end of the bout are unique time periods, only bouts with more than one motif in duration were used. Behaviorally inconsistent bouts were excluded for clarity of visualization; however, results are consistent when including them in calculating the ITPC. (p<0.006 for all Z > 5 for all subjects and days; all non-black time-frequency pints in this plot are above the significance threshold).
(TIF)

S10 Fig Unique LFP phase preferences at each syllable onset (z007, day 1). (A) Polar histogram of the phase for each LFP frequency band at the labeled start of all instances of a given syllable or the introductory note over the course of one day (Day 1), for one bird (z007) (S3 Table). (B) ITPC resultant vector length for each frequency over time relative to the labeled start of each syllable or introductory note (0 ms) over randomly downselected instances from (A) to match the number of instances per syllable. (C) Rayleigh Z-statistic of the ITPC over the same time and frequencies as (B). (p<0.007 for all Z > 5 for all syllables and the introductory note; all non-black time-frequency pints in this plot are above the significance threshold). For (B) and (C) the number of instances (n = 71) are equal for all syllables and the introductory note, and are set by the syllable class with the fewest renditions.
(TIF)

S11 Fig Unique LFP phase preferences at each syllable onset (z020). (A) Polar histogram of the phase for each LFP frequency band at the labeled start of all instances of a given syllable or the introductory note over the course of one day (Day 1), for one bird (z020) (S4 Table). (B) ITPC resultant vector length for each frequency over time relative to the labeled start of each syllable or introductory note (0 ms) over randomly downselected instances from (A) to match the number of instances per syllable. (C) Rayleigh Z-statistic of the ITPC over the same time and frequencies as (B). For (B) and (C) the number of instances (n = 91) are equal for all syllables and the introductory note, and are set by the syllable class with the fewest renditions. (D) Polar histogram of the phase for each LFP frequency band at the labeled start of all instances of a given syllable over the course of one day (Day 2), for one bird (z020) (S4 Table). (E) ITPC resultant vector length for each frequency over time relative to the labeled start of each syllable (0 ms) over randomly downselected instances from (D). (F) Rayleigh Z-statistic of the ITPC over the same time and frequencies as (E). For (E) and (F) the number of instances (n = 75) are equal for all syllables and the introductory note, and are set by the syllable class with the fewest renditions. (p<0.007 for all Z > 5 for all syllables and the introductory note for both days; all non-black time-frequency pints in this plot are above the significance threshold).
(TIF)

S12 Fig Unique LFP phase preferences at each syllable onset (z017). (A) Polar histogram of the phase for each LFP frequency band at the labeled start of all instances of a given syllable over the course of one day (Day 1), for one bird (z020) (S5 Table). (B) ITPC resultant vector length for each frequency over time relative to the labeled start of each syllable (0 ms) over randomly downselected instances from (A) to match the number of instances per syllable. (C) Rayleigh Z-statistic of the ITPC over the same time and frequencies as (B). For (B) and (C) the number of instances (n = 79) are equal for all syllables, and set by the syllable class with the fewest renditions. (D) Polar histogram of the phase for each LFP frequency band at the labeled start of all instances of a given syllable over the course of one day (Day 2), for one bird (z020) (S5 Table). (E) ITPC resultant vector length for each frequency over time relative to the labeled start of each syllable (0 ms) over randomly downselected instances from (D). (F) Rayleigh Z-statistic of the ITPC over the same time and frequencies as (E). For (E) and (F) the number of instances (n = 66) are equal for all syllables, and set by the syllable class with the fewest renditions. (p<0.007 for all Z > 5 for all syllables for both days; all non-black time-frequency pints in this plot are above the significance threshold).
(TIF)

S13 Fig Unique LFP phase preferences for sparsely used intra-motif note onset (z017). (A) Polar histogram of the phase for each LFP frequency band at the labeled start of all instances (n = 52) of syllable 7 over the course of one day (Day 1), for bird z017. (B) ITPC resultant vector length for each frequency over time relative to the labeled start of syllable 7 (0 ms) over the same instances as in (A). (C) Rayleigh Z-statistic of the ITPC over the same time and frequencies as (B). (D) Polar histogram of the phase for each LFP frequency band at the labeled start of all instances (n = 41) of syllable 7 over the course of one day (Day 2), for bird z017. (E) ITPC resultant vector length for each frequency over time relative to the labeled start of syllable 7 (0 ms) over the same instances as in (D). (F) Rayleigh Z-statistic of the ITPC over the same time and frequencies as (E). (p<0.007 for all Z > 5 for both syllables; all non-black time-frequency pints in this plot are above the significance threshold).
(TIF)

S14 Fig Detailed view of the syllable phase preference to syllable onset for z007. Detailed rendering of the phase preference to syllable onset for z007 shown in Fig **7**A. Each row shows a different vocalization type, which includes the five syllables of the motif and the introductory note. Each column shows a different frequency band and is organized top to bottom from least to greatest. As such they are the polar plots for the (A) 4–8 Hz band, (B) 8–12 Hz band, (C) 25–35 Hz band, (D) 35–50 Hz band, and (E) the 50–70 Hz band. The number of instances have been balanced to match the class with the least number of instances (n = 98) for each class.
(TIF)

S15 Fig Both Phase and Power has independent/additive Information to classify syllable identity. Channel-adding curves calculated by repeatedly training classifiers with an increasing number of randomly selected channels (see Methods). Channel-adding curves of classifier performances with either (A) all information about phase removed, (B) all information about power removed, or (C) with both phase and power used as independent features. Each row corresponds to data from the highest-yield day for each bird. z007 n = 98 for each class n = 7 (1, 2, 3, 4, 5, i, Silence), z020 n = 91 for each class n = 6 (1, 2, 3, 4, i, Silence), and for z017 n = 52* for each class n = 8 (1, 2, 3, 4, 5, 6, 7, Silence). Error bars represent the standard deviation over the bootstrapped analysis using n = 5,000 repetitions across 5 cross- validation folds. The p-value for all of the binomial chances calculated for each bird was 0.05. *The number of instances for each class was limited by Syllable 7, which is an intra-motif note.
(TIF)

S16 Fig Channel-adding Curves for the second highest-yield days across subjects. Channel-adding curves calculated by repeatedly training classifiers with an increasing number of randomly selected channels (see Methods). Channel-adding curves of classifier performances with either (A) all phase related information removed, (B) all power related information removed, or (C) with both phase and power used as independent features. Each row corresponds to data from the second highest yielding day for each bird. z007 n = 71 for each class n = 7 (1, 2, 3, 4, 5, i, Silence), z020 n = 75 for each class n = 6 (1, 2, 3, 4, i, Silence), and for z017 n = 41* for each class n = 8 (1, 2, 3, 4, 5, 6, 7, Silence). Error bars represent the standard deviation over the bootstrapped analysis using n = 5,000 repetitions across 5 cross-validation folds. The p-value for all of the binomial chances calculated for each bird was 0.05. *The number of instances for each class was limited by Syllable 7, which is an intra-motif note.
(TIF)

S17 Fig Difference in Decoding Accuracy between Phase Only and Power Only Classification. Classification accuracy for each high-yield day for each bird with 15 channels of neural data when all phase-related information is removed, left, and all power-related information is removed, right, for (A) 4–8 Hz band, (B) 8–12 Hz band, (C) 25–35 Hz band, (D) 35–50 Hz band, and 50–70 Hz band.
(TIF)

S18 Fig Onset detection and branch behavior analysis using LFP features for Subject z020. (A) State diagram of z020’s observed song structure. Syllable colors are the same as in Fig **3**. (B) Example motif from the highest-yield day for subject z020. Annotated behavior (top) using the same color scheme as in Fig **3**, sound pressure waveform and the corresponding time-aligned spectrogram (middle), and the time-varying naïve confidence of the onset prediction (bottom) for each syllable in this example motif. Confidence signal traces are the same color as the syllable they are meant to predict. (C) Boxplot of onset prediction times relative to the labeled onset time for both of the high-yield days for z020. The order of each feature used is the same, going left to right, as is shown in Fig **10**. The time window that the neural based predictor must make a prediction within is represented by the dotted black line (see Methods). Statistical significance was calculated using the one-sided Wilcoxon signed-rank test, and ^▲^ denotes results that are not statistically significant when using the Benjamini-Hochberg False Discovery Rate. All other results p<0.05 and q<0.05.
(TIF)

S19 Fig Onset detection and branch behavior analysis using LFP features for Subject z017. (A) State diagram of z017’s observed song structure. Syllable colors are the same as in Fig **3**. (B) Example motif from the highest yield day for subject z017. Annotated behavior (top) using the same color scheme as in Fig **3**, sound pressure waveform and the corresponding time-aligned spectrogram (middle), and the time-varying naïve confidence of the onset prediction (bottom) for each syllable in this example motif. Confidence signal traces are the same color as the syllable they are meant to predict. (C) Boxplot of onset prediction times relative to the labeled onset time for both of the high-yield days for z017. The order of each feature used is the same, going left to right, as shown in Fig **10**. The time window that the neural based predictor must make a prediction within is represented by the dotted black line (see Methods). Statistical significance was calculated using the one-sided Wilcoxon signed-rank test, and denotes results that are not statistically significant when using the Benjamini-Hochberg False Discovery Rate. All other results p<0.05 and q<0.05.
(TIF)

S20 Fig Onset detection across all high-yield days. **Boxplots of onset prediction times relative to the labeled onset time for each bird for every syllable for two highest-yielding days.** Each column reflects the result for syllable number within the motif. Each row is for a specific bird with (A) corresponding to z007, (B) corresponding to z020, and (C) corresponding to z017. The order of each feature used is the same, going left to right: first is the stereotyped onset time using only the deterministic behavior, next is the results using all of the neural features, then each frequency band only in order from least to greatest (4–8 Hz, 8–12 Hz, 25–35 Hz, 35–50 Hz, and finally 50–70 Hz). The recording day designation number refers to the chronological order that the recordings took place. Statistical significance was calculated using the one-sided Wilcoxon signed-rank test, and ^▲^ denotes results that were not statistically significant when using the Benjamini-Hochberg False Discovery Rate. All other results p<0.05 and q<0.05.
(TIF)
