# Supplementary figures and images for: Local field potentials in a pre-motor region predict learned vocal sequences
Source: PLoS Comput Biol. 2021 Sep 23;17(9):e1008100. doi: 10.1371/journal.pcbi.1008100 (PMC8460039; doi:10.1371/journal.pcbi.1008100)

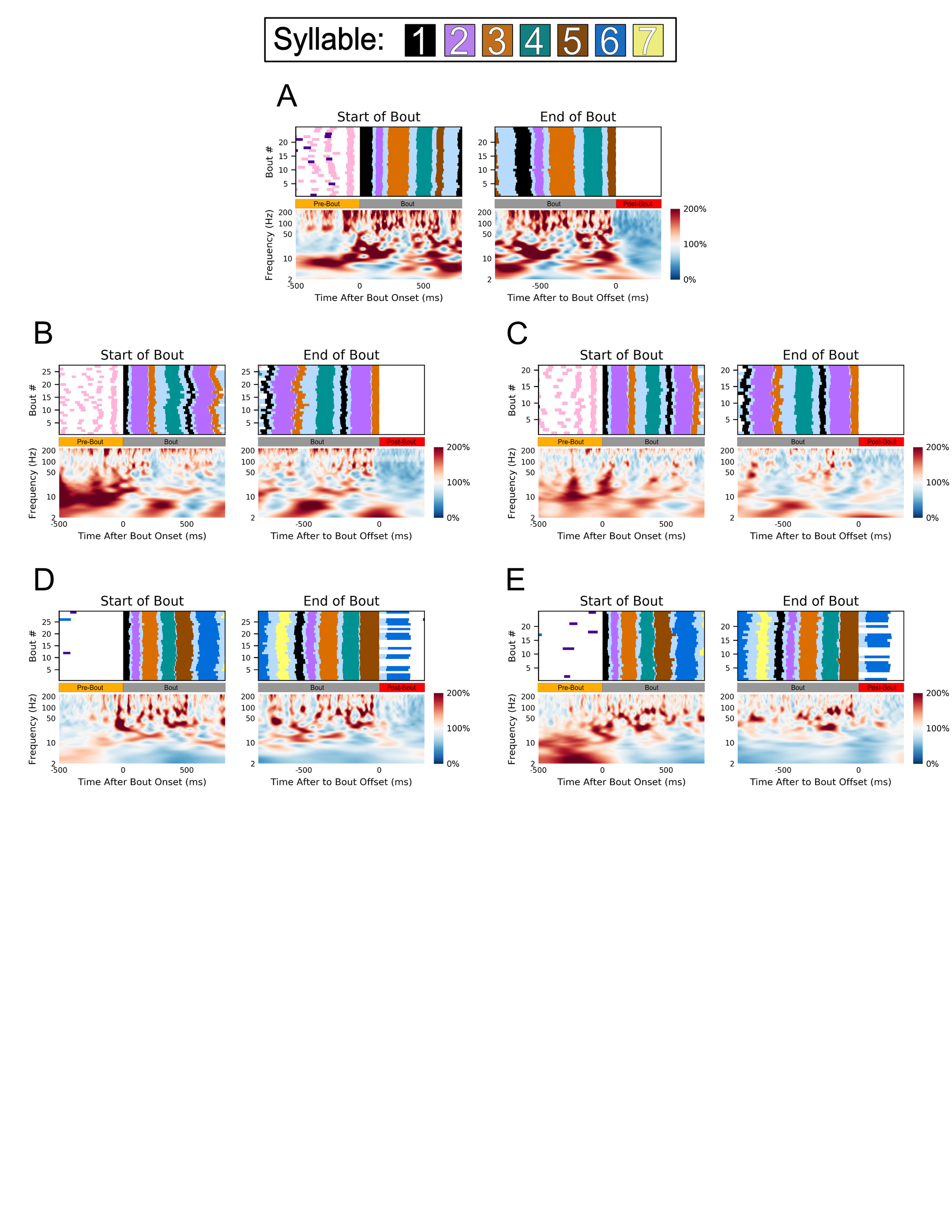

Supplement: S1 Fig — Averaged spectrotemporal power activity (see Methods) aligned to the start of the first motif in the bout, left, and the last motif in the bout, right, for the additional recording days that are not plotted in Fig 4. Shown above all results is a behavioral raster showing the time course of the behavior being averaged. (A) The averaged results for the second highest-yielding day, designated Day 1, for z007 (n = 25 Bouts). The other subjects’ results are show as follows; (B) z020’s first high yield day (n = 29 Bouts), (C) z020’s second high-yield day (n = 25 Bouts), (D) z017’s first high-yield day (n = 27 Bouts), and (E) z017’s second high-yield day (n = 21 Bouts). As z017 would end its bout on either syllable ‘5’ or ‘6’, the end of the bout was aligned to syllable ‘5’. No dynamic-time warping was used. To ensure the start and end of the bout are unique time periods, only bouts with more than one motif in duration were used. Behaviorally inconsistent bouts were excluded for clarity of visualization; however, results are consistent when including them in the analysis. (TIF) [file pcbi.1008100.s012.tif]

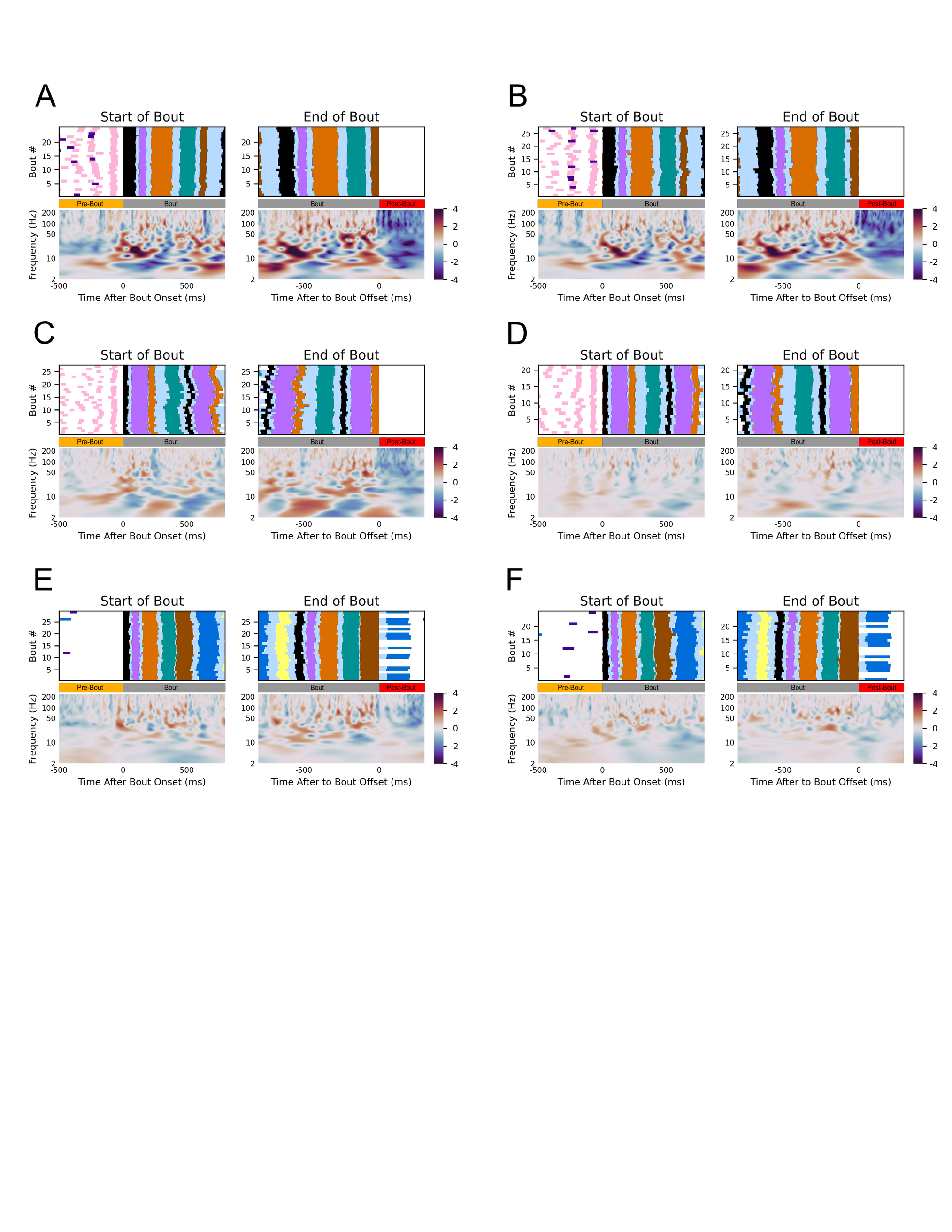

Supplement: S2 Fig — Cross trial z-scored ratings of average spectrograms (see Methods) aligned to the start of the first motif in the bout, left, and the last motif in the bout, right, for each recording day. The z-score metric normalizes the modulation in LFP power at each frequency and time point by its standard deviation across trials. Thus the metric quantifies the number of standard deviations, as measured across trials, between the mean LFP power at each timepoint/frequency and the mean LFP power across the VAP. Shown above all results is a behavioral raster showing the time course of the behavior being evaluated. (A) The z-scored results for the first high yield day, designated Day 1, for z007 (n = 25 Bouts) (B) The z-scored results for the second high-yield day, designated Day 2, for z007 (n = 27 Bouts). The other subjects’ results are shown as follows; (C) z020’s first high yield day (n = 29 Bouts), (D) z020’s second high-yield day (n = 25 Bouts), (E) z017’s first high-yield day (n = 27 Bouts), and (F) z017’s second high-yield day (n = 21 Bouts). As z017 would end its bout on either syllable ‘5’ or ‘6’, the end of the bout was aligned to syllable ‘5’. No dynamic-time warping was used. To ensure the start and end of the bout are unique time periods, only bouts with more than one motif in duration were used. Behaviorally inconsistent bouts were excluded for clarity of visualization; however, results are consistent when including them in the analysis. (TIF) [file pcbi.1008100.s013.tif]

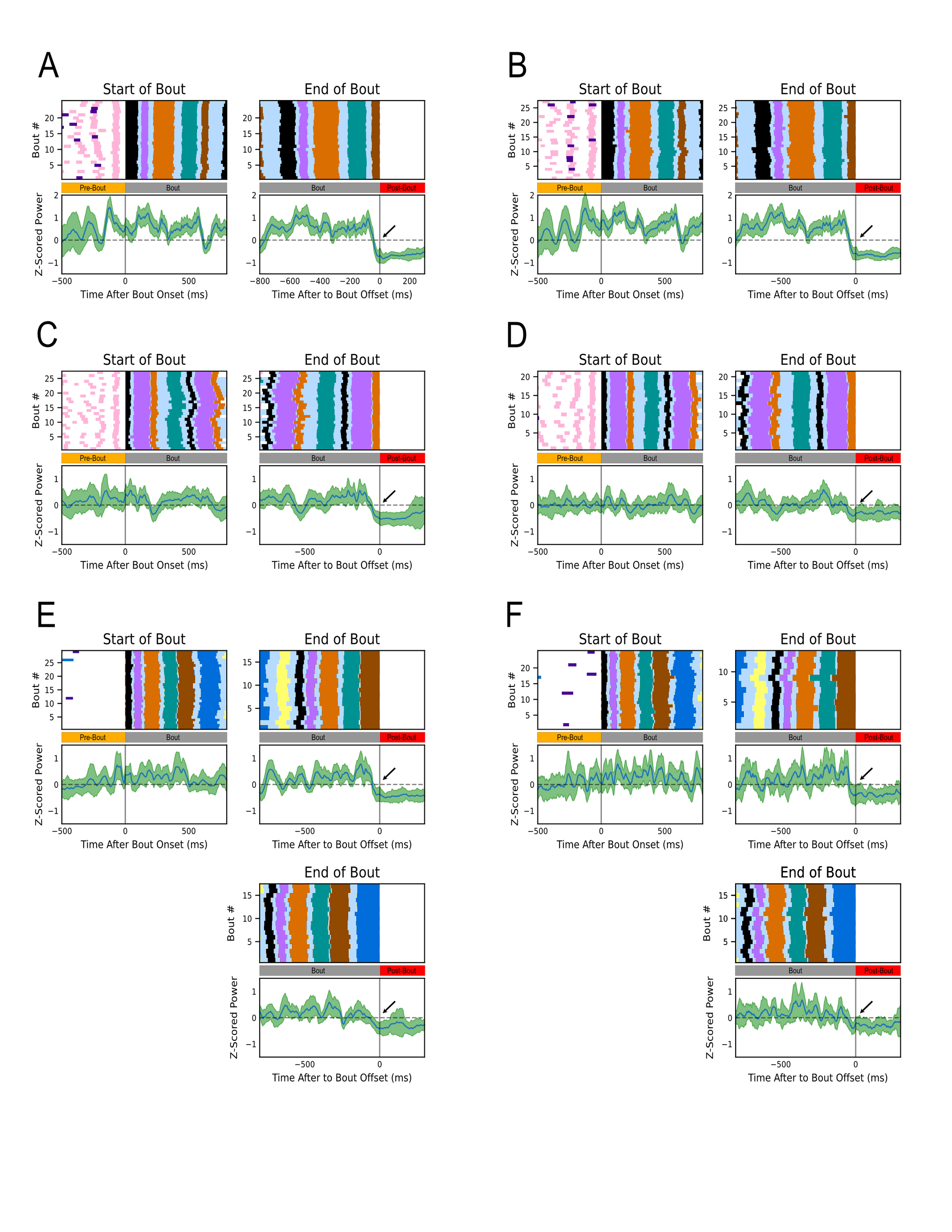

Supplement: S3 Fig — Z-scored changes in power of the 50–200 Hz band aligned to the start of the first motif in the bout, left, and the last motif in the bout, right, for each high-yield recording day. Black traces in each subpanel show the mean, and the green shading is the standard deviation. The end of the bout, and the subsequent drop in power are annotated by a black arrow. Above all results is a behavioral raster showing the time course of the behavior being averaged. (A) The results of the first-high yield day of z007 (n = 27 Bouts). (B) The results for the second high-yield day for z007 (n = 25 Bouts). The other subjects results are shown as follows; (C) z020’s first high-yield day (n = 29 Bouts), (D) z020’s second high-yield day (n = 25 Bouts), (E) z017’s first high yield day (n = 27 Bouts), and (F) z017’s second high-yield day (n = 21 Bouts). As z017 ends its bout on either syllable ‘5’ or ‘6’, both types of bout endings are shown separately. No dynamic-time warping is used. To ensure that the start and end of the bout are unique time periods, only bouts with more than one motif in duration are used. Behaviorally inconsistent bouts are excluded for clarity of visualization; however, results are consistent when including them in the analysis. (TIF) [file pcbi.1008100.s014.tif]

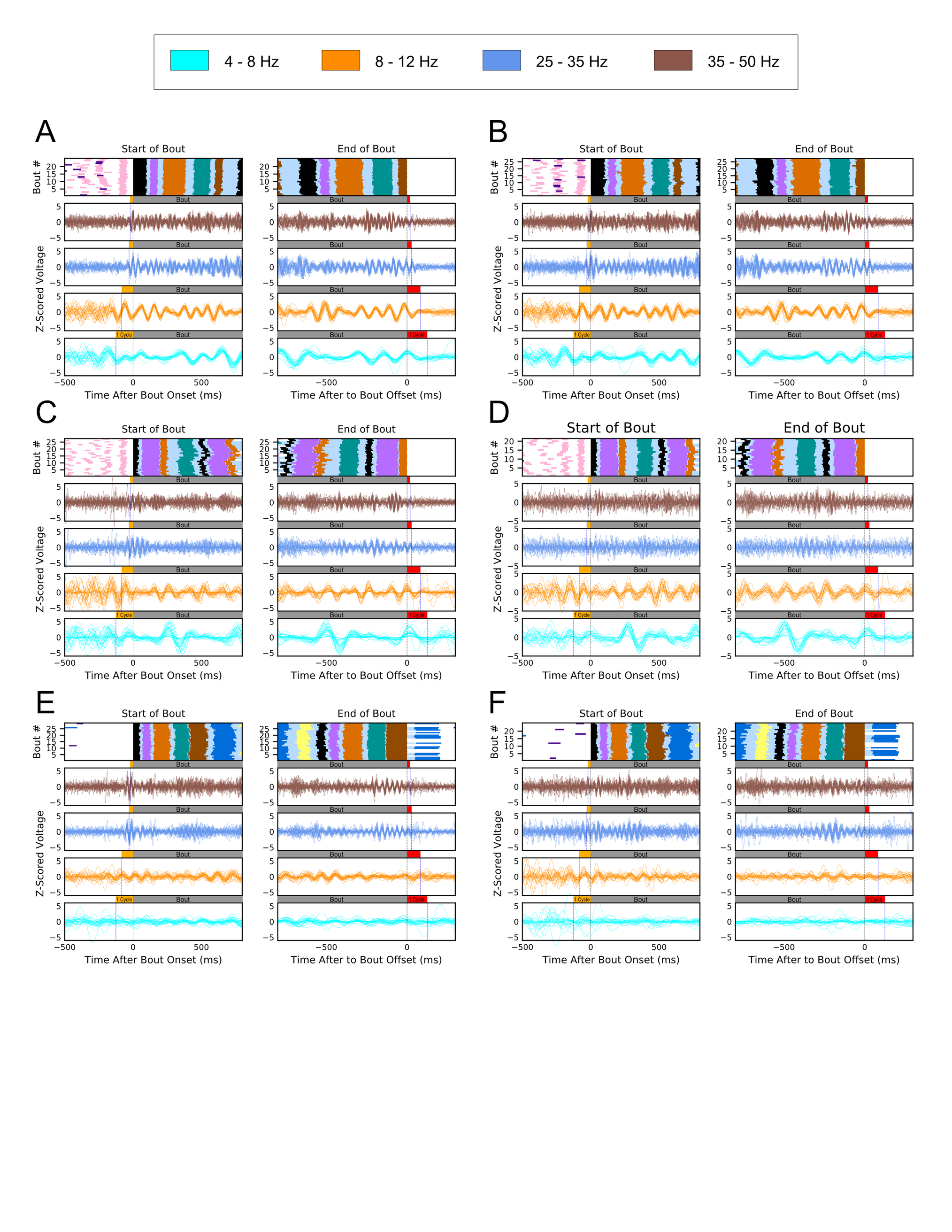

Supplement: S4 Fig — Single-trial z-scored LFP traces of four narrowband frequency bands aligned to the start of the first motif in the bout, left, and the last motif in the bout, right, for each high-yield recording day. Each trace is colored its respective narrowband frequency. Shown above all results are behavioral rasters showing the time course of the behaviors being shown. The black line in each row below the behavior shows the time point the trials are aligned to, and the blue line denotes the time prior to (left) or after (right) one full cycle of the highest frequency in the narrowband frequency. (A) The results of the first high-yield Day of z007 (n = 27 Bouts) (B) The results for the second high-yield day for z007 (n = 25 Bouts). The other subjects’ results are shown as follows; (C) z020’s first high-yield day (n = 29 Bouts), (D) z020’s second high-yield day (n = 25 Bouts), (E) z017’s first high-yield day (n = 27 Bouts), and (F) z017’s second high-yield day (n = 21 Bouts). As z017 would end its bout on either syllable ‘5’ or ‘6’, the end of the bout was aligned to syllable ‘5’. No dynamic-time warping was used. To ensure that the start and end of the bout are unique time periods, only bouts with more than one motif in duration were used. Behaviorally inconsistent bouts were excluded for clarity of visualization; however, results are consistent when including them in the analysis. (TIF) [file pcbi.1008100.s015.tif]

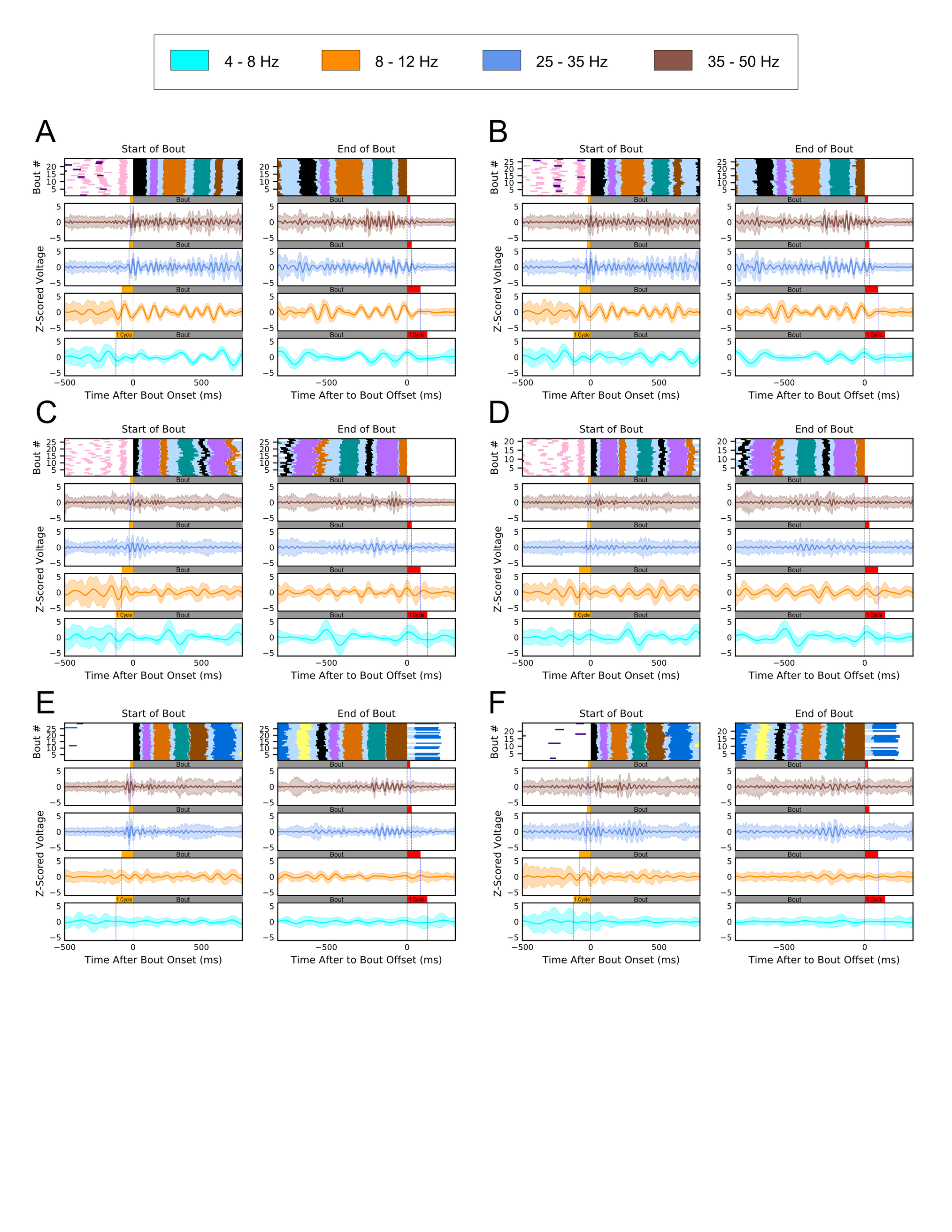

Supplement: S5 Fig — The mean and standard deviation of the z-scored LFP traces of four narrowband frequency bands aligned to the start of the first motif in the bout, left, and the last motif in the bout, right, for each high-yield recording day. Each row is colored its respective narrowband frequency. Above all results is a behavioral raster showing the time course of the behaviors being shown. The black line in each row below the behavior shows the time point that the trials are aligned to, and the blue line denotes the time prior to (left) or after (right) one full cycle of the highest frequency in the narrowband frequency. (A) The results of the first high yield day of z007 (n = 27 Bouts) (B) The results for the second high-yield day for z007 (n = 25 Bouts). The other subjects’ results are shown as follows; (C) z020’s first high-yield day (n = 29 Bouts), (D) z020’s second high-yield day (n = 25 Bouts), (E) z017’s first high-yield day (n = 27 Bouts), and (F) z017’s second high-yield day (n = 21 Bouts). As z017 would end its bout on either syllable ‘5’ or ‘6’, the end of the bout was aligned to syllable ‘5’. No dynamic-time warping was used. To ensure that the start and end of the bout are unique time periods, only bouts with more than one motif in duration were used. Behaviorally inconsistent bouts were excluded for clarity of visualization, however results are consistent when including them in the analysis. (TIF) [file pcbi.1008100.s016.tif]

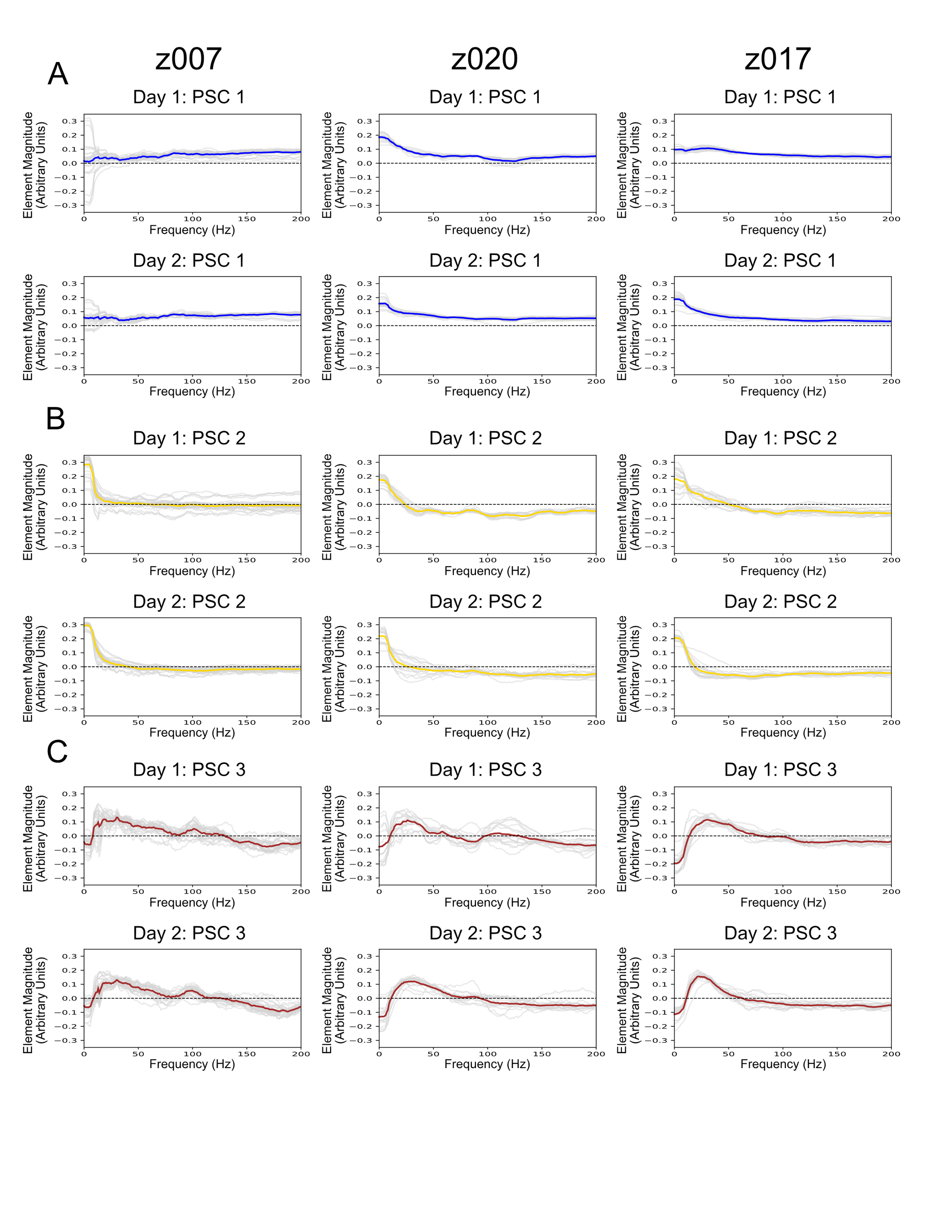

Supplement: S6 Fig — (A) The 1st Principle spectral components for every channel (light grey) with the mean across channels in Blue for all High Yield Days for each subject. (B) The 2nd Principle spectral components for every channel (light grey) with the mean across channels (golden-yellow) for all High Yield Days for each subject. (C) The 3rd Principle spectral components for every channel (light grey) with the mean across channels (burgundy) for all High Yield Days for each subject. (TIF) [file pcbi.1008100.s017.tif]

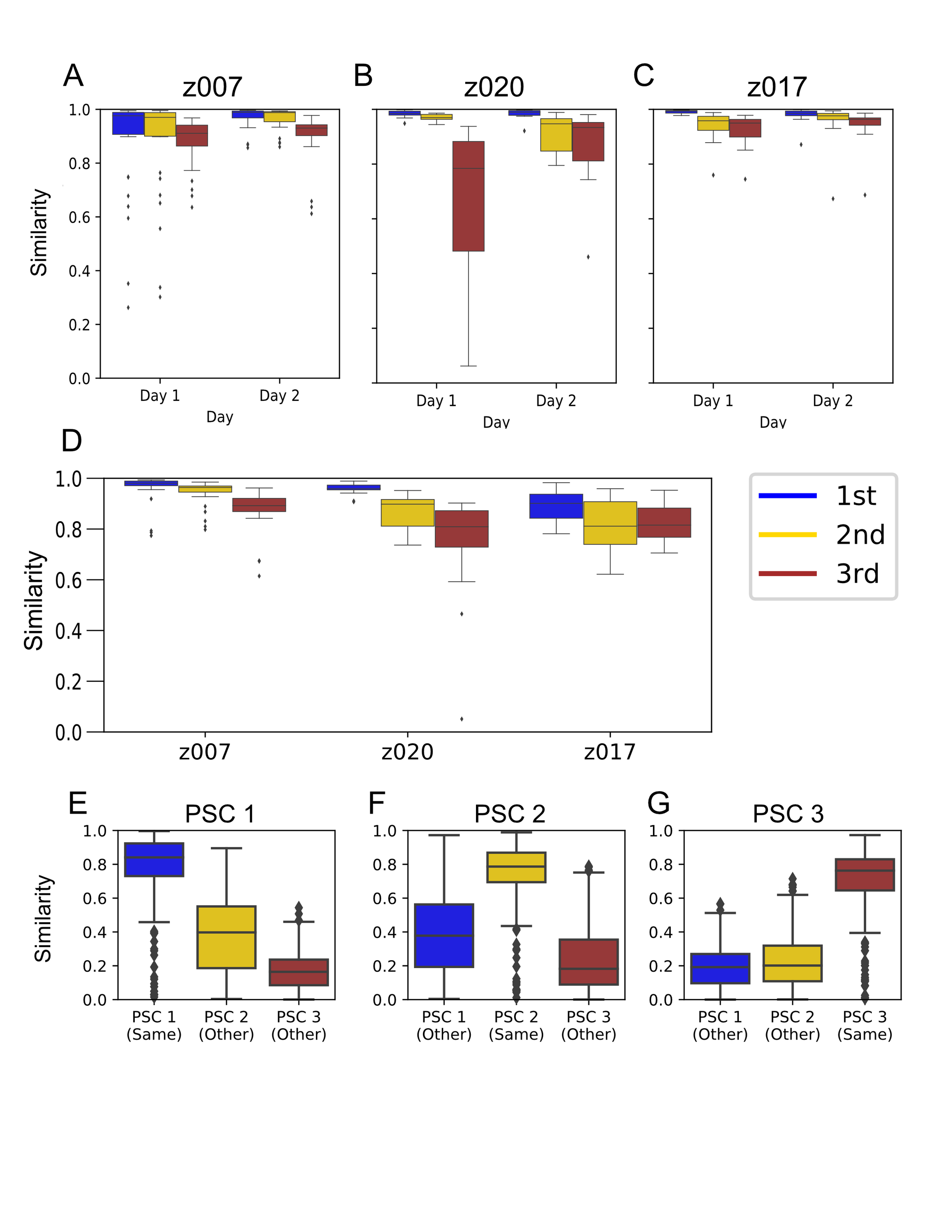

Supplement: S7 Fig — Boxplot of the distribution of cosine similarity metric values between a template, which is created by taking the mean across the sign-aligned PSC of all channels for a specific PSC. The cosine similarity matrix ranges from 1 and -1, however, the absolute value of the metric is shown (see Methods). (A) The cosine similarity of each channel’s PSC to the same recording day’s template PSC for both high-yield days for subject z007. (B) The cosine similarity of each channel’s PSC to the same recording day’s template PSC for both high-yield days for subject z020. (C) The cosine similarity of each channel’s PSC to the same recording day’s template PSC for both high-yield days for subject z017. (D) The cosine similarity of each channel’s PSC from the second high-yield day with the template of the PSC from the first high-yield day. (E) All templates for PSC 1 compared either with the PSC 1 for the other two birds, same, or the PSC for one of the other PSCs for the other two birds, other. (F) All templates for PSC 2 compared either with the PSC 2 for the other two birds, same, or the PSC for one of the other PSCs for the other two birds, other. (E) All templates for PSC 3 compared either with the PSC3 for the other two birds, same, or the PSC for one of the other PSCs for the other two birds, other. (TIF) [file pcbi.1008100.s018.tif]

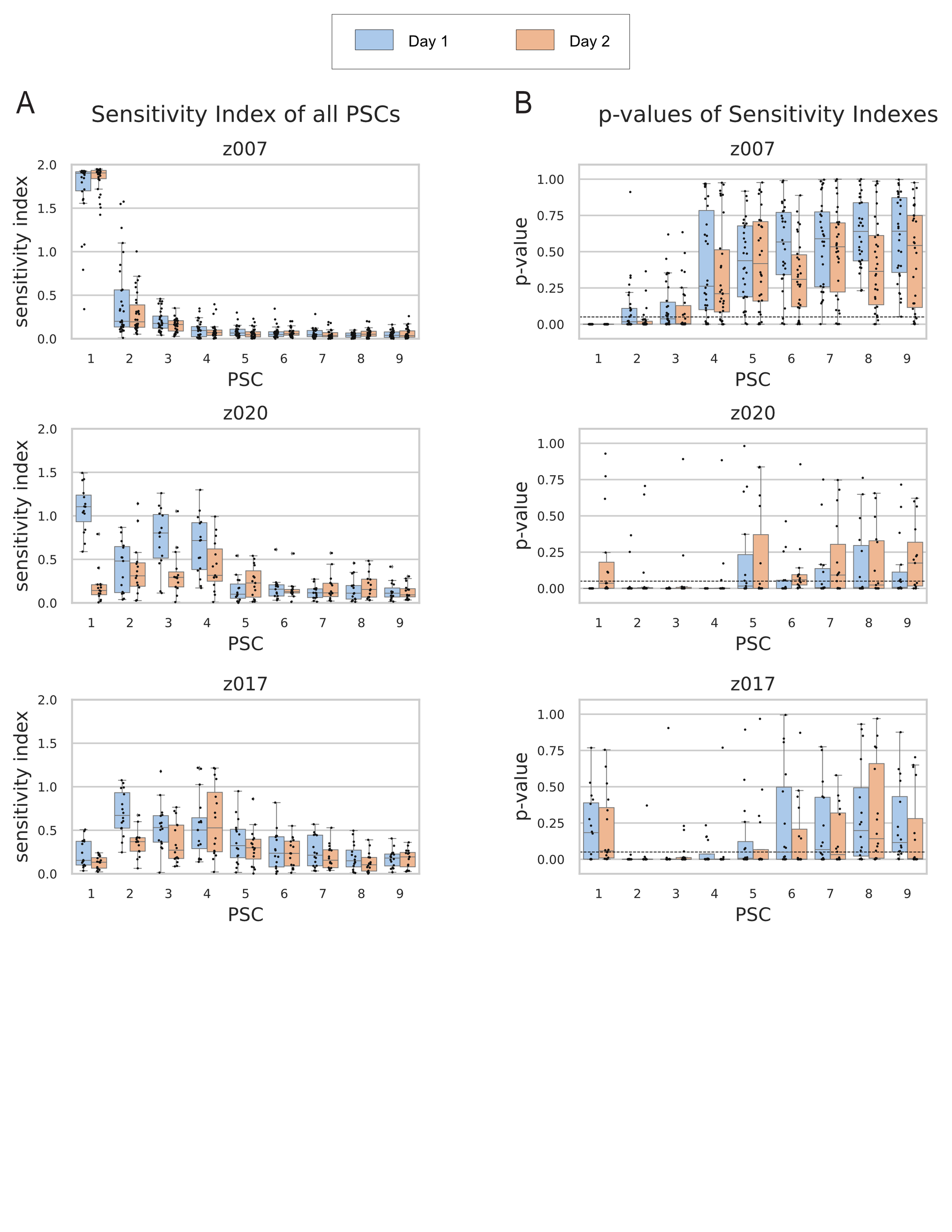

Supplement: S8 Fig — (A) Box plots of the sensitivity indexes (d’) for the separation of the Active and Inactive trials for all channels using the first 9 PSCs. The plots show the distribution of values for both high yield days for all three subjects. (B) Blox plots of the p-values of the sensitivity indexes shown in (A) when tested against a bootstrapped shuffle control (N = 20,000 Shuffles). (TIF) [file pcbi.1008100.s019.tif]

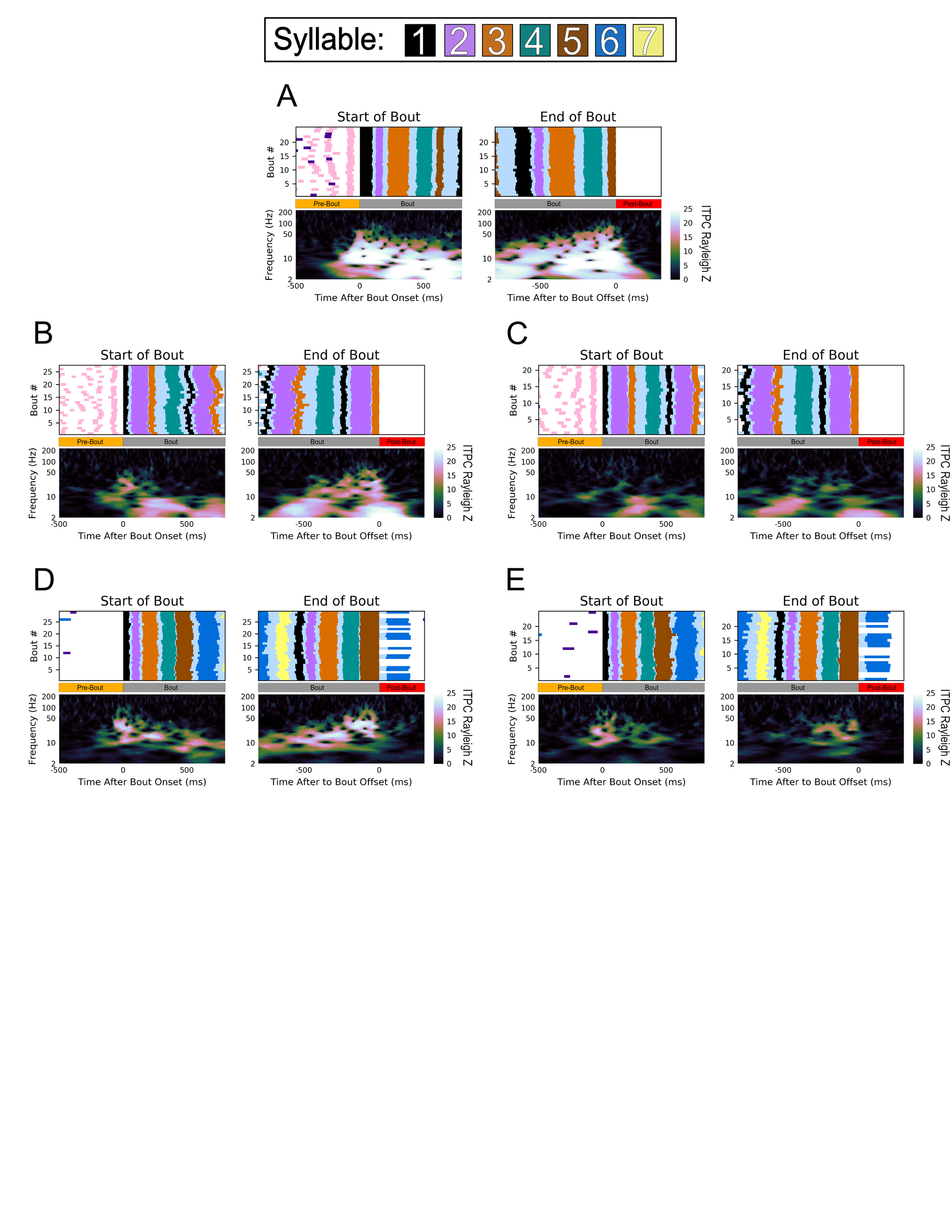

Supplement: S9 Fig — ITPC of LFP aligned to the start of the first motif in the bout, left, and the last motif in the bout, right, for the additional recording days that are not plotted in Fig 6. Shown above all results is a behavioral raster showing the time course of the behavior being averaged. (A) The averaged results for the second highest-yielding day, designated Day 1, for z007 (n = 25 bouts). The other subjects’ results are show as follows; (B) z020’s first high-yield day (n = 29 bouts), (C) z020’s second high-yield day (n = 25 bouts), (D) z017’s first high-yield day (n = 27 bouts), and (E) z017’s second high-yield day (n = 21 bouts). As z017 would end its bout on either syllable ‘5’ or ‘6’, the end of the bout was aligned to syllable ‘5’. No dynamic-time warping was used. To ensure that the start and end of the bout are unique time periods, only bouts with more than one motif in duration were used. Behaviorally inconsistent bouts were excluded for clarity of visualization; however, results are consistent when including them in calculating the ITPC. (p<0.006 for all Z > 5 for all subjects and days; all non-black time-frequency pints in this plot are above the significance threshold). (TIF) [file pcbi.1008100.s020.tif]

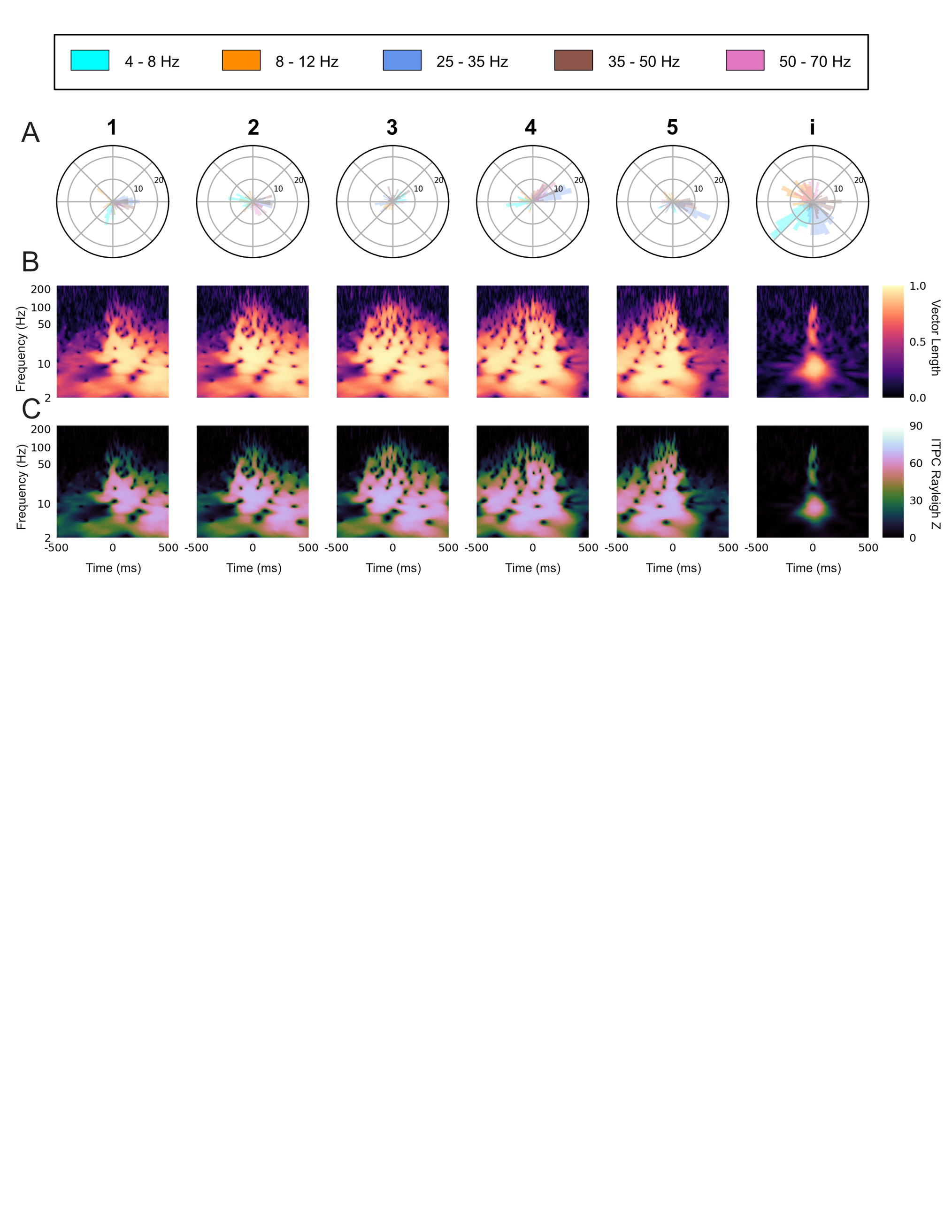

Supplement: S10 Fig — (A) Polar histogram of the phase for each LFP frequency band at the labeled start of all instances of a given syllable or the introductory note over the course of one day (Day 1), for one bird (z007) (S3 Table). (B) ITPC resultant vector length for each frequency over time relative to the labeled start of each syllable or introductory note (0 ms) over randomly downselected instances from (A) to match the number of instances per syllable. (C) Rayleigh Z-statistic of the ITPC over the same time and frequencies as (B). (p<0.007 for all Z > 5 for all syllables and the introductory note; all non-black time-frequency pints in this plot are above the significance threshold). For (B) and (C) the number of instances (n = 71) are equal for all syllables and the introductory note, and are set by the syllable class with the fewest renditions. (TIF) [file pcbi.1008100.s021.tif]

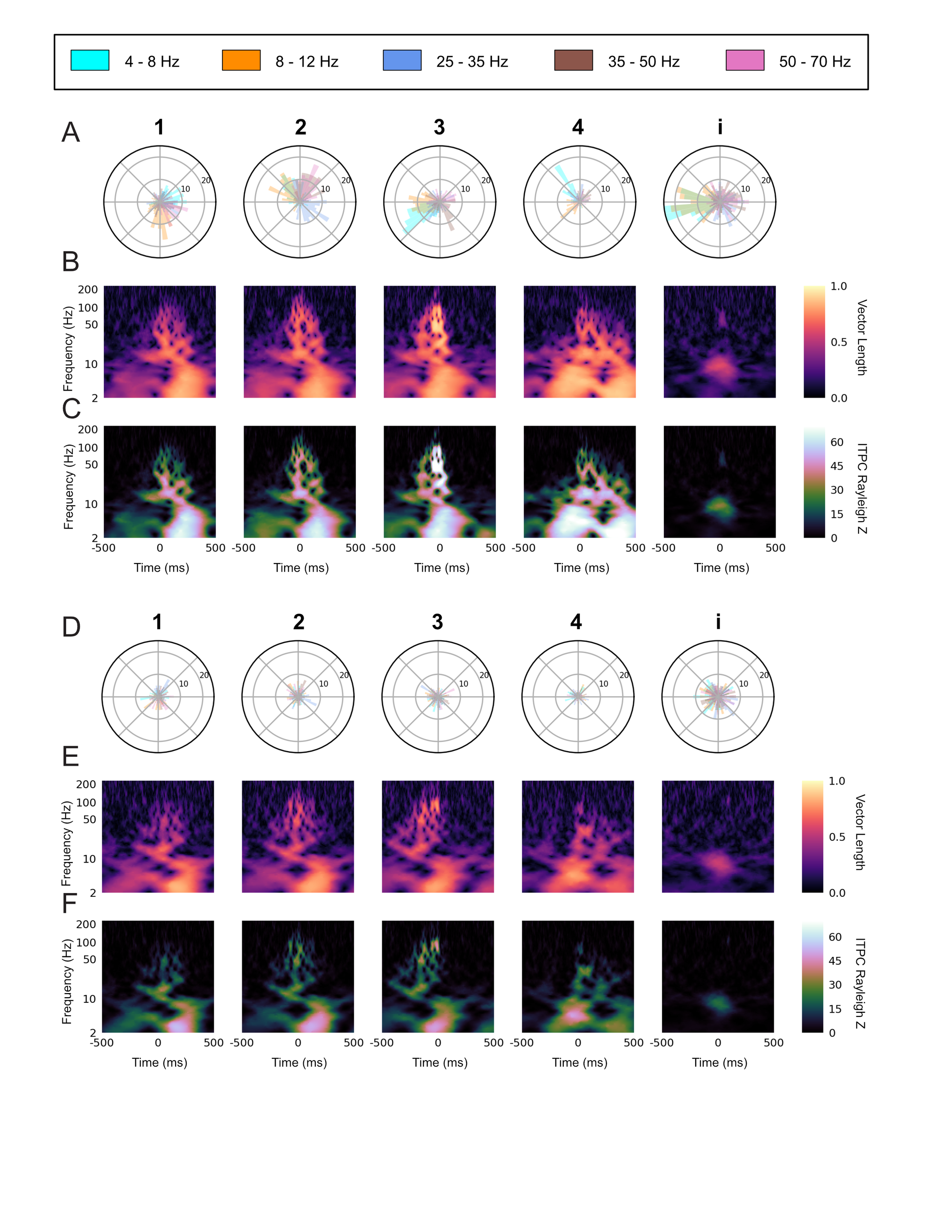

Supplement: S11 Fig — (A) Polar histogram of the phase for each LFP frequency band at the labeled start of all instances of a given syllable or the introductory note over the course of one day (Day 1), for one bird (z020) (S4 Table). (B) ITPC resultant vector length for each frequency over time relative to the labeled start of each syllable or introductory note (0 ms) over randomly downselected instances from (A) to match the number of instances per syllable. (C) Rayleigh Z-statistic of the ITPC over the same time and frequencies as (B). For (B) and (C) the number of instances (n = 91) are equal for all syllables and the introductory note, and are set by the syllable class with the fewest renditions. (D) Polar histogram of the phase for each LFP frequency band at the labeled start of all instances of a given syllable over the course of one day (Day 2), for one bird (z020) (S4 Table). (E) ITPC resultant vector length for each frequency over time relative to the labeled start of each syllable (0 ms) over randomly downselected instances from (D). (F) Rayleigh Z-statistic of the ITPC over the same time and frequencies as (E). For (E) and (F) the number of instances (n = 75) are equal for all syllables and the introductory note, and are set by the syllable class with the fewest renditions. (p<0.007 for all Z > 5 for all syllables and the introductory note for both days; all non-black time-frequency pints in this plot are above the significance threshold). (TIF) [file pcbi.1008100.s022.tif]

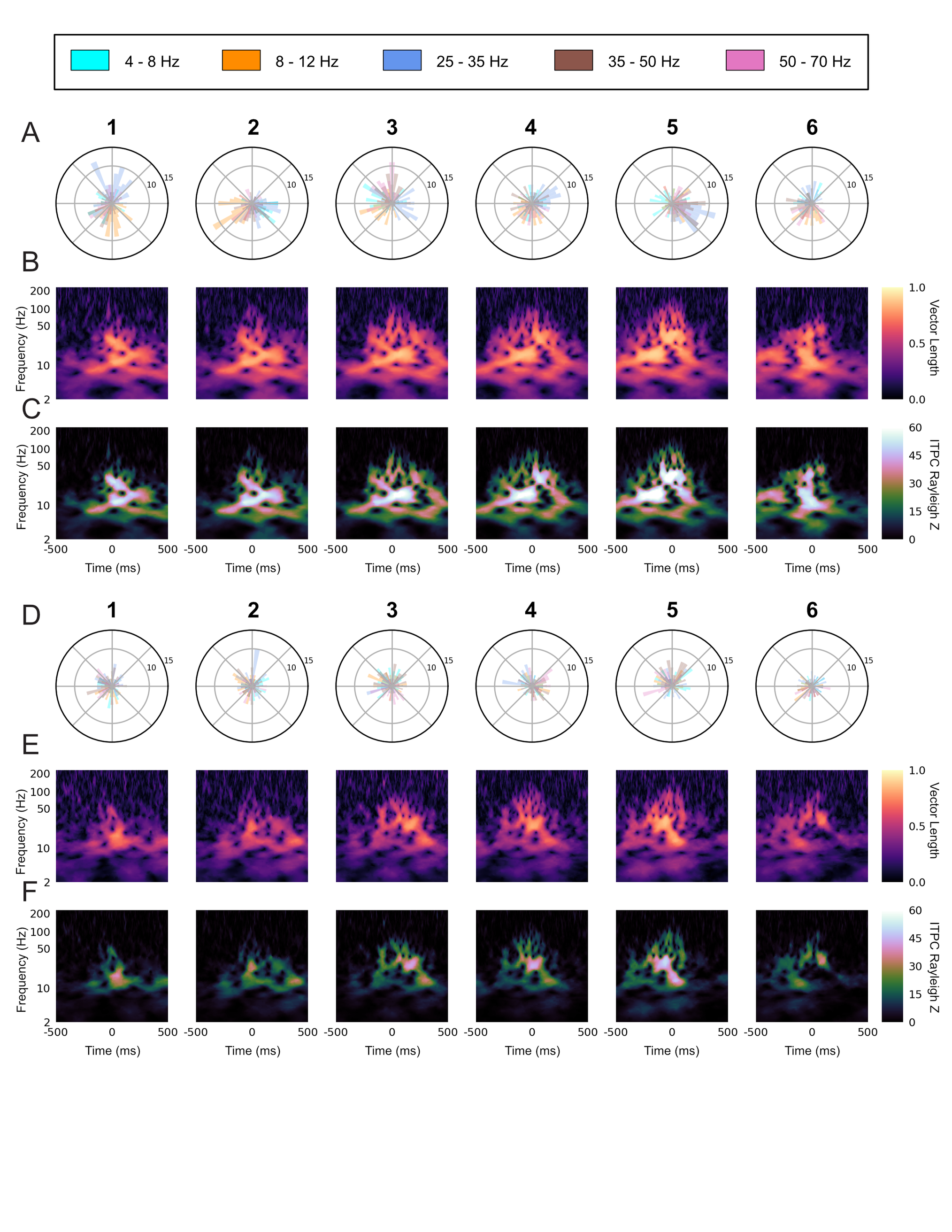

Supplement: S12 Fig — (A) Polar histogram of the phase for each LFP frequency band at the labeled start of all instances of a given syllable over the course of one day (Day 1), for one bird (z020) (S5 Table). (B) ITPC resultant vector length for each frequency over time relative to the labeled start of each syllable (0 ms) over randomly downselected instances from (A) to match the number of instances per syllable. (C) Rayleigh Z-statistic of the ITPC over the same time and frequencies as (B). For (B) and (C) the number of instances (n = 79) are equal for all syllables, and set by the syllable class with the fewest renditions. (D) Polar histogram of the phase for each LFP frequency band at the labeled start of all instances of a given syllable over the course of one day (Day 2), for one bird (z020) (S5 Table). (E) ITPC resultant vector length for each frequency over time relative to the labeled start of each syllable (0 ms) over randomly downselected instances from (D). (F) Rayleigh Z-statistic of the ITPC over the same time and frequencies as (E). For (E) and (F) the number of instances (n = 66) are equal for all syllables, and set by the syllable class with the fewest renditions. (p<0.007 for all Z > 5 for all syllables for both days; all non-black time-frequency pints in this plot are above the significance threshold). (TIF) [file pcbi.1008100.s023.tif]

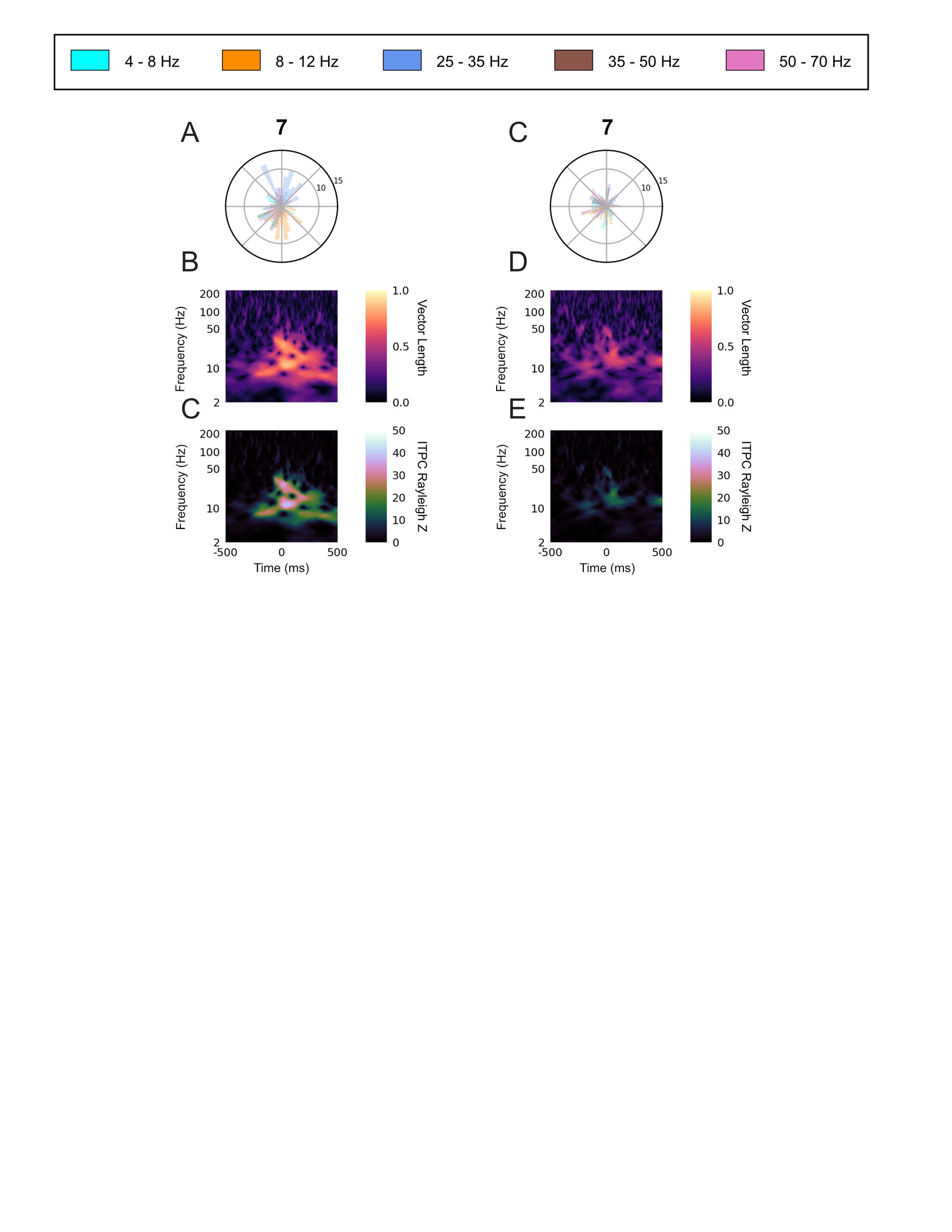

Supplement: S13 Fig — (A) Polar histogram of the phase for each LFP frequency band at the labeled start of all instances (n = 52) of syllable 7 over the course of one day (Day 1), for bird z017. (B) ITPC resultant vector length for each frequency over time relative to the labeled start of syllable 7 (0 ms) over the same instances as in (A). (C) Rayleigh Z-statistic of the ITPC over the same time and frequencies as (B). (D) Polar histogram of the phase for each LFP frequency band at the labeled start of all instances (n = 41) of syllable 7 over the course of one day (Day 2), for bird z017. (E) ITPC resultant vector length for each frequency over time relative to the labeled start of syllable 7 (0 ms) over the same instances as in (D). (F) Rayleigh Z-statistic of the ITPC over the same time and frequencies as (E). (p<0.007 for all Z > 5 for both syllables; all non-black time-frequency pints in this plot are above the significance threshold). (TIF) [file pcbi.1008100.s024.tif]

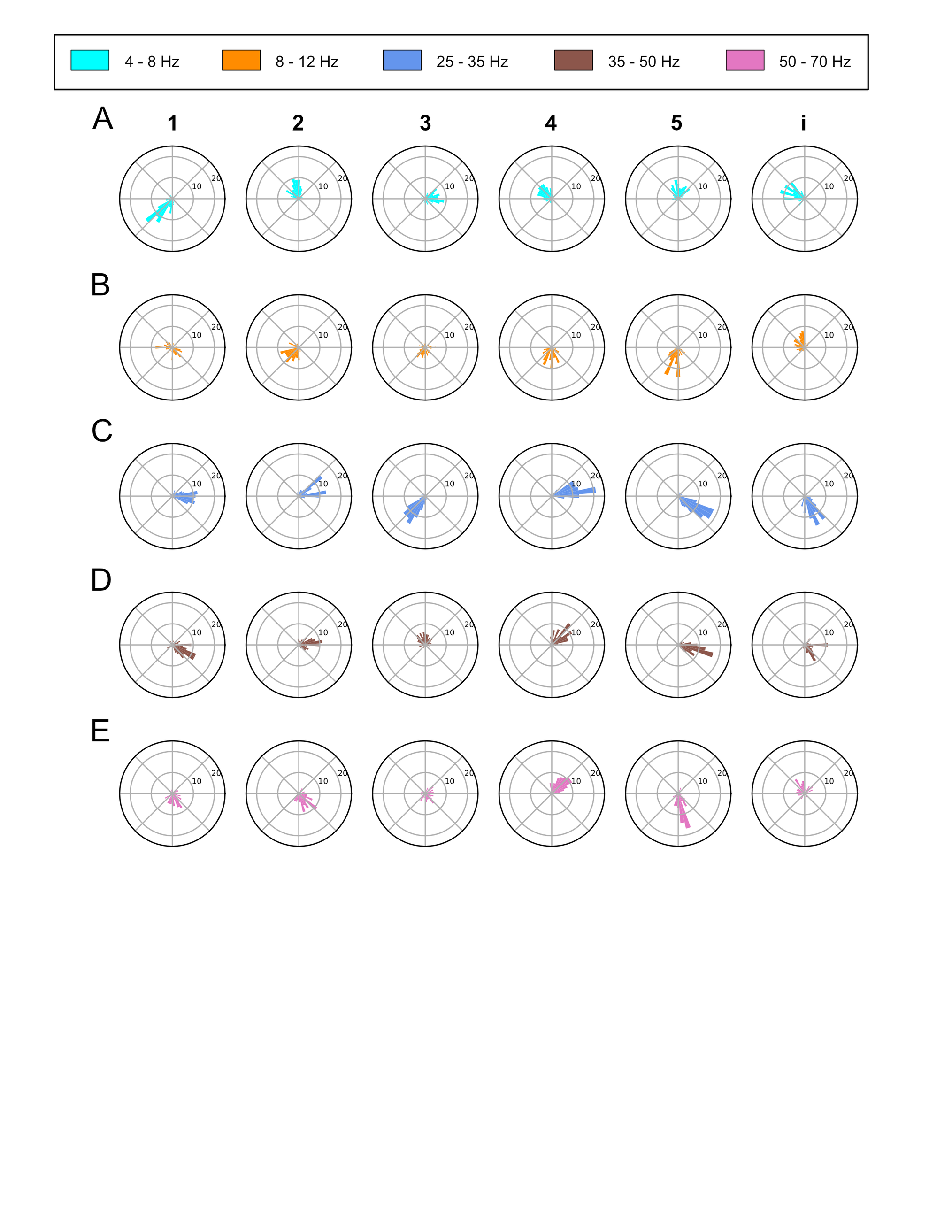

Supplement: S14 Fig — Detailed rendering of the phase preference to syllable onset for z007 shown in Fig 7A. Each row shows a different vocalization type, which includes the five syllables of the motif and the introductory note. Each column shows a different frequency band and is organized top to bottom from least to greatest. As such they are the polar plots for the (A) 4–8 Hz band, (B) 8–12 Hz band, (C) 25–35 Hz band, (D) 35–50 Hz band, and (E) the 50–70 Hz band. The number of instances have been balanced to match the class with the least number of instances (n = 98) for each class. (TIF) [file pcbi.1008100.s025.tif]

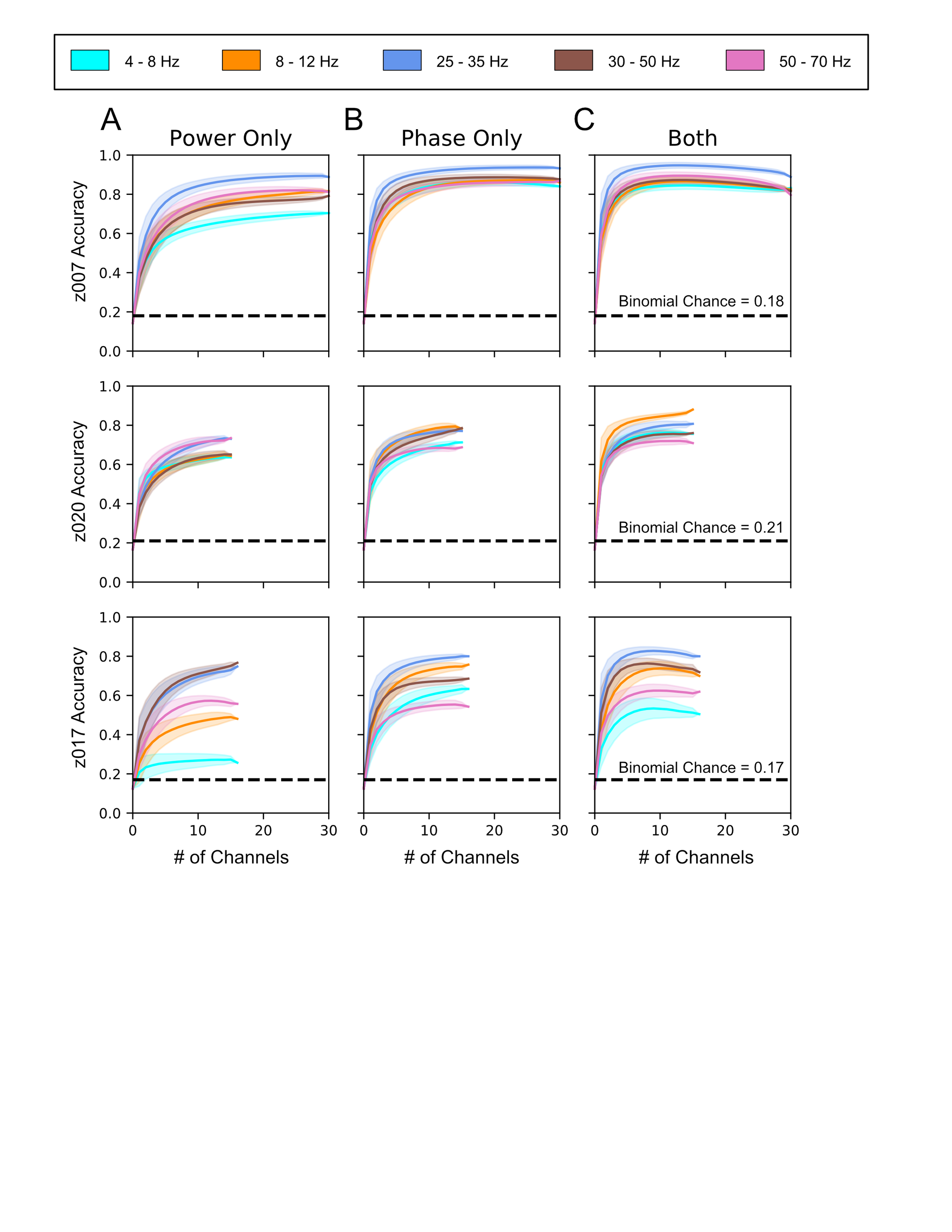

Supplement: S15 Fig — Channel-adding curves calculated by repeatedly training classifiers with an increasing number of randomly selected channels (see Methods). Channel-adding curves of classifier performances with either (A) all information about phase removed, (B) all information about power removed, or (C) with both phase and power used as independent features. Each row corresponds to data from the highest-yield day for each bird. z007 n = 98 for each class n = 7 (1, 2, 3, 4, 5, i, Silence), z020 n = 91 for each class n = 6 (1, 2, 3, 4, i, Silence), and for z017 n = 52* for each class n = 8 (1, 2, 3, 4, 5, 6, 7, Silence). Error bars represent the standard deviation over the bootstrapped analysis using n = 5,000 repetitions across 5 cross- validation folds. The p-value for all of the binomial chances calculated for each bird was 0.05. *The number of instances for each class was limited by Syllable 7, which is an intra-motif note. (TIF) [file pcbi.1008100.s026.tif]

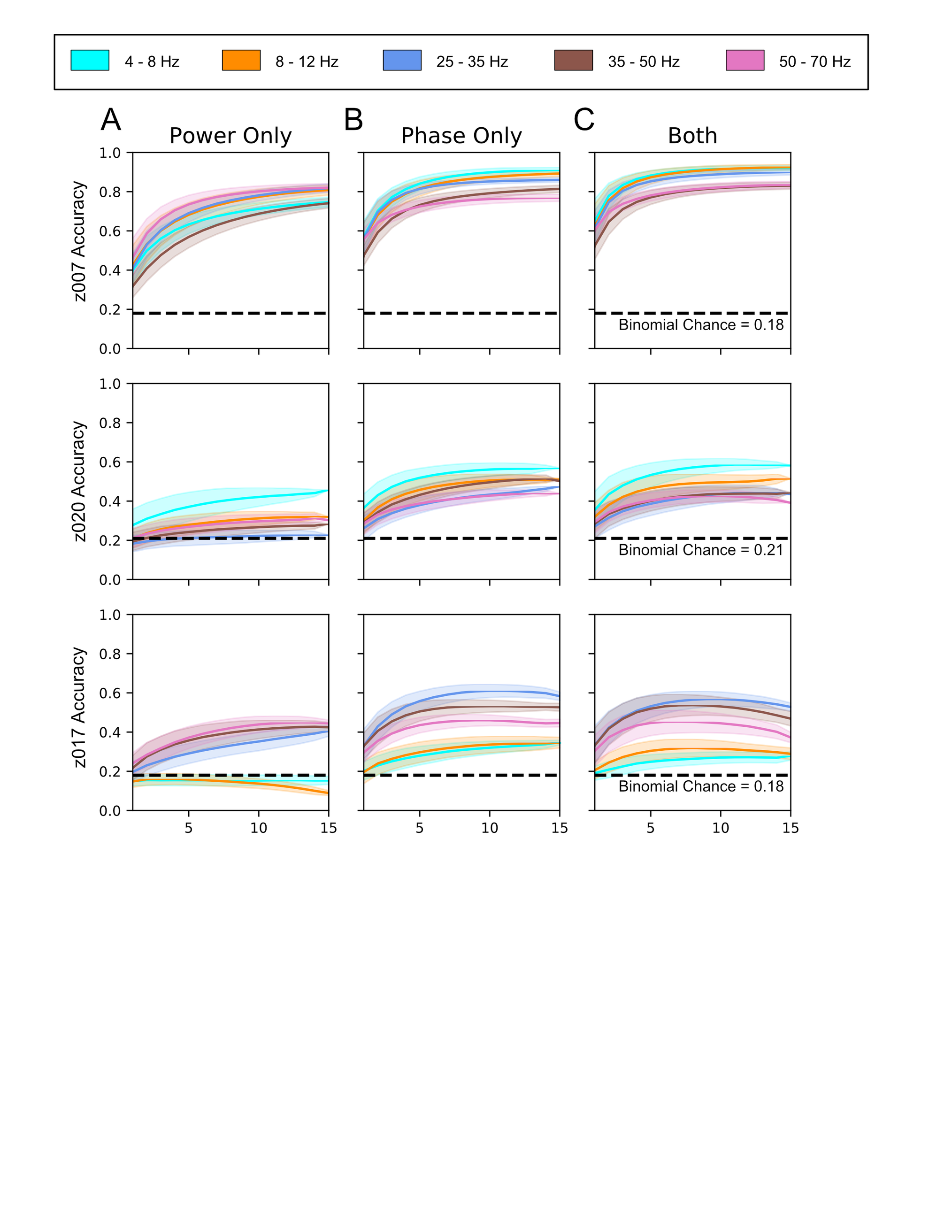

Supplement: S16 Fig — Channel-adding curves calculated by repeatedly training classifiers with an increasing number of randomly selected channels (see Methods). Channel-adding curves of classifier performances with either (A) all phase related information removed, (B) all power related information removed, or (C) with both phase and power used as independent features. Each row corresponds to data from the second highest yielding day for each bird. z007 n = 71 for each class n = 7 (1, 2, 3, 4, 5, i, Silence), z020 n = 75 for each class n = 6 (1, 2, 3, 4, i, Silence), and for z017 n = 41* for each class n = 8 (1, 2, 3, 4, 5, 6, 7, Silence). Error bars represent the standard deviation over the bootstrapped analysis using n = 5,000 repetitions across 5 cross-validation folds. The p-value for all of the binomial chances calculated for each bird was 0.05. *The number of instances for each class was limited by Syllable 7, which is an intra-motif note. (TIF) [file pcbi.1008100.s027.tif]

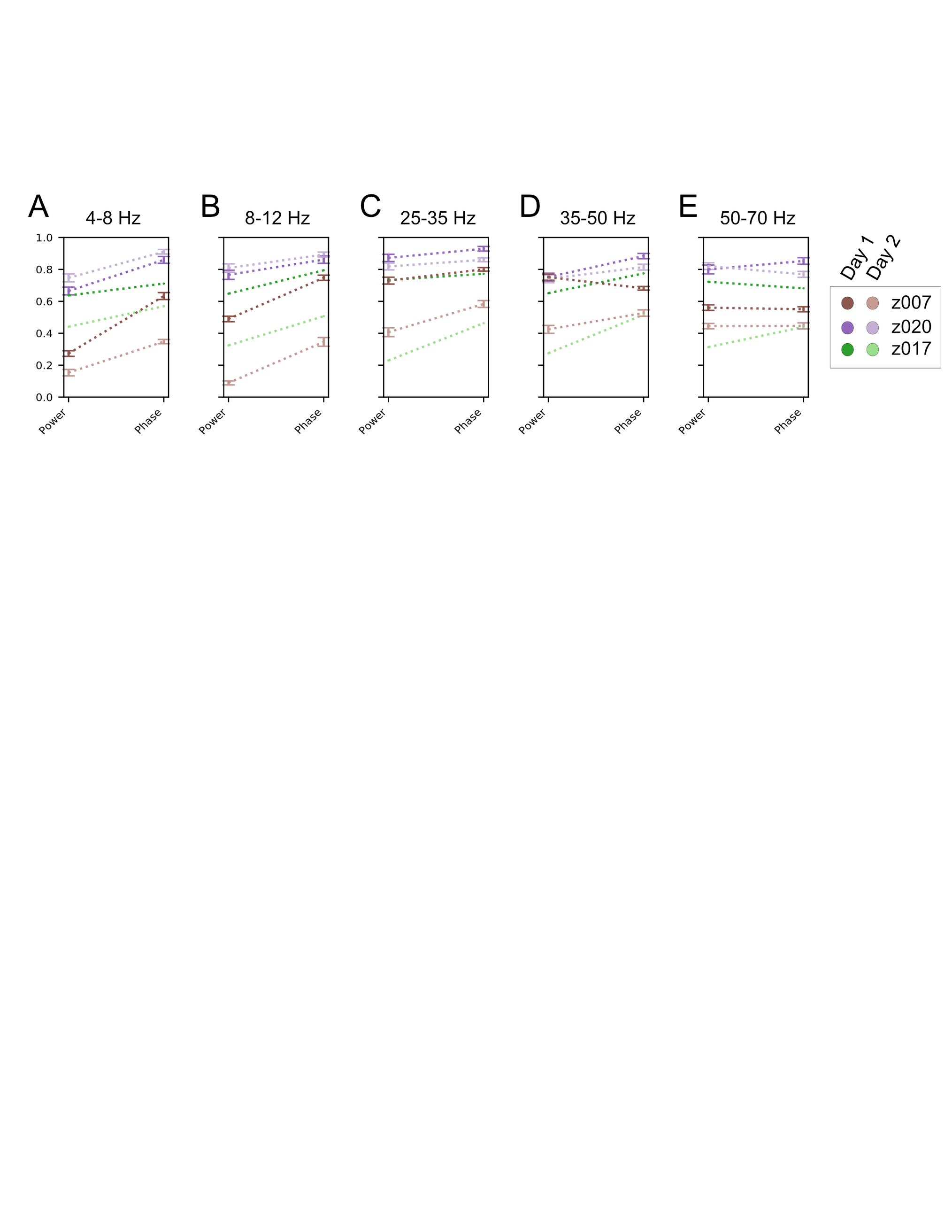

Supplement: S17 Fig — Classification accuracy for each high-yield day for each bird with 15 channels of neural data when all phase-related information is removed, left, and all power-related information is removed, right, for (A) 4–8 Hz band, (B) 8–12 Hz band, (C) 25–35 Hz band, (D) 35–50 Hz band, and 50–70 Hz band. (TIF) [file pcbi.1008100.s028.tif]

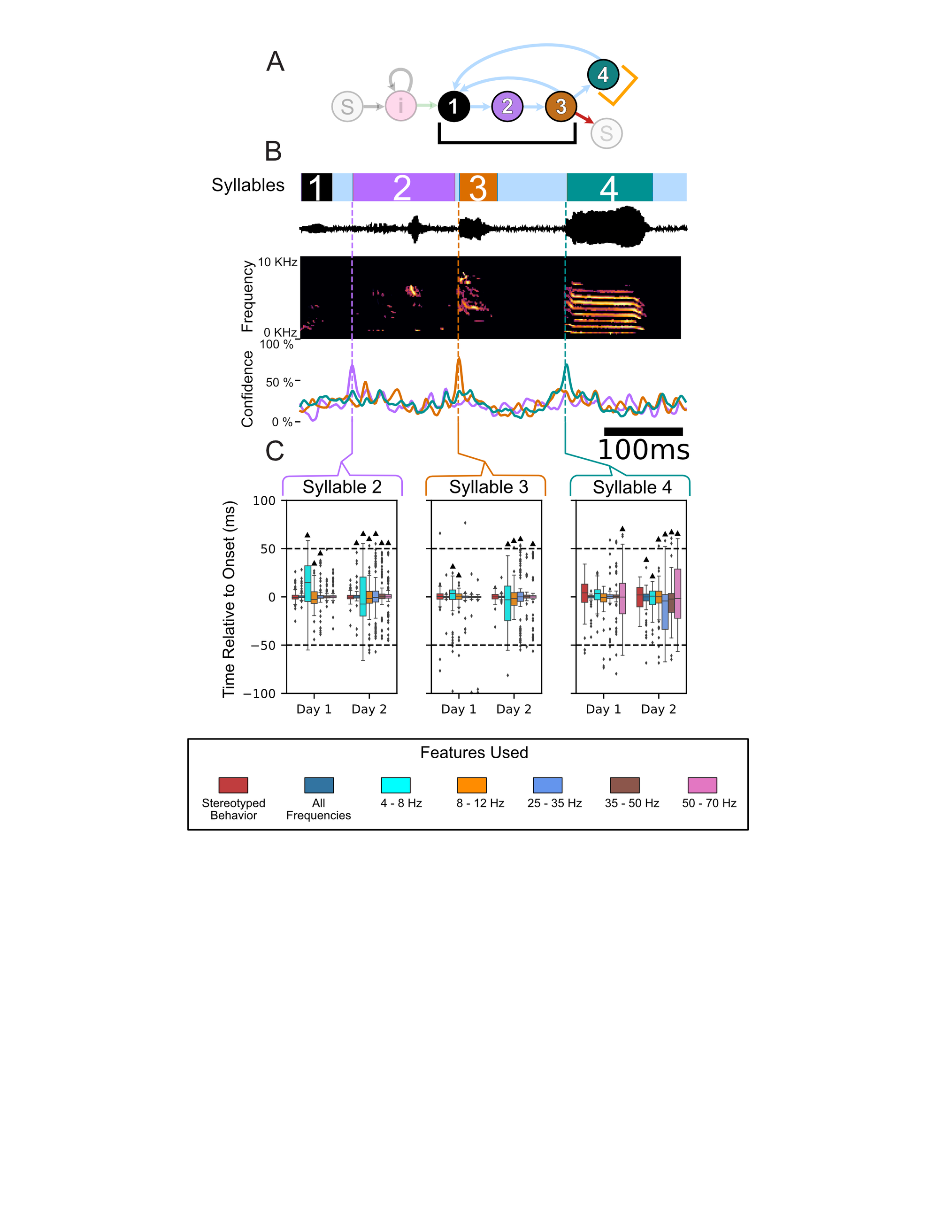

Supplement: S18 Fig — (A) State diagram of z020’s observed song structure. Syllable colors are the same as in Fig 3. (B) Example motif from the highest-yield day for subject z020. Annotated behavior (top) using the same color scheme as in Fig 3, sound pressure waveform and the corresponding time-aligned spectrogram (middle), and the time-varying naïve confidence of the onset prediction (bottom) for each syllable in this example motif. Confidence signal traces are the same color as the syllable they are meant to predict. (C) Boxplot of onset prediction times relative to the labeled onset time for both of the high-yield days for z020. The order of each feature used is the same, going left to right, as is shown in Fig 10. The time window that the neural based predictor must make a prediction within is represented by the dotted black line (see Methods). Statistical significance was calculated using the one-sided Wilcoxon signed-rank test, and ▲ denotes results that are not statistically significant when using the Benjamini-Hochberg False Discovery Rate. All other results p<0.05 and q<0.05. (TIF) [file pcbi.1008100.s029.tif]

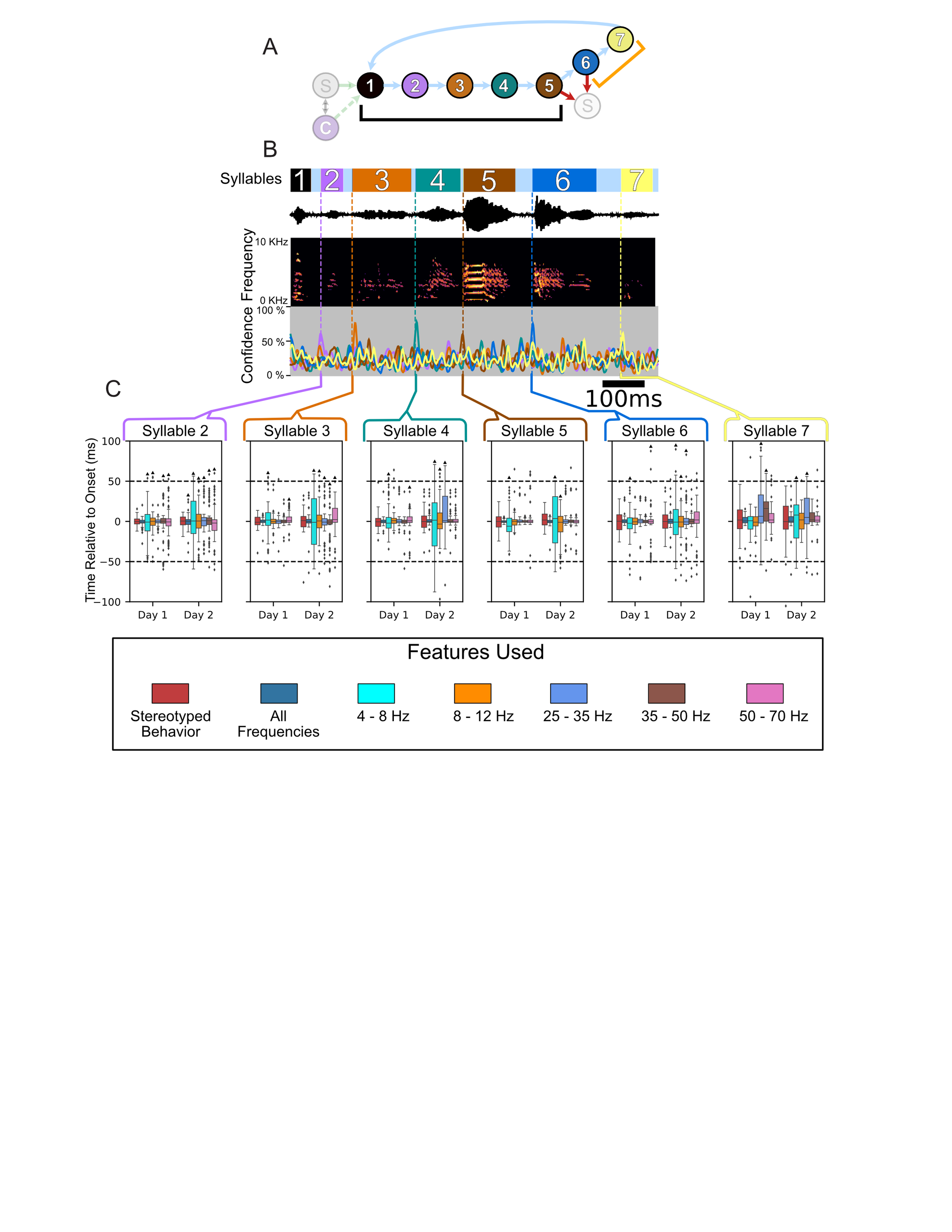

Supplement: S19 Fig — (A) State diagram of z017’s observed song structure. Syllable colors are the same as in Fig 3. (B) Example motif from the highest yield day for subject z017. Annotated behavior (top) using the same color scheme as in Fig 3, sound pressure waveform and the corresponding time-aligned spectrogram (middle), and the time-varying naïve confidence of the onset prediction (bottom) for each syllable in this example motif. Confidence signal traces are the same color as the syllable they are meant to predict. (C) Boxplot of onset prediction times relative to the labeled onset time for both of the high-yield days for z017. The order of each feature used is the same, going left to right, as shown in Fig 10. The time window that the neural based predictor must make a prediction within is represented by the dotted black line (see Methods). Statistical significance was calculated using the one-sided Wilcoxon signed-rank test, and denotes results that are not statistically significant when using the Benjamini-Hochberg False Discovery Rate. All other results p<0.05 and q<0.05. (TIF) [file pcbi.1008100.s030.tif]

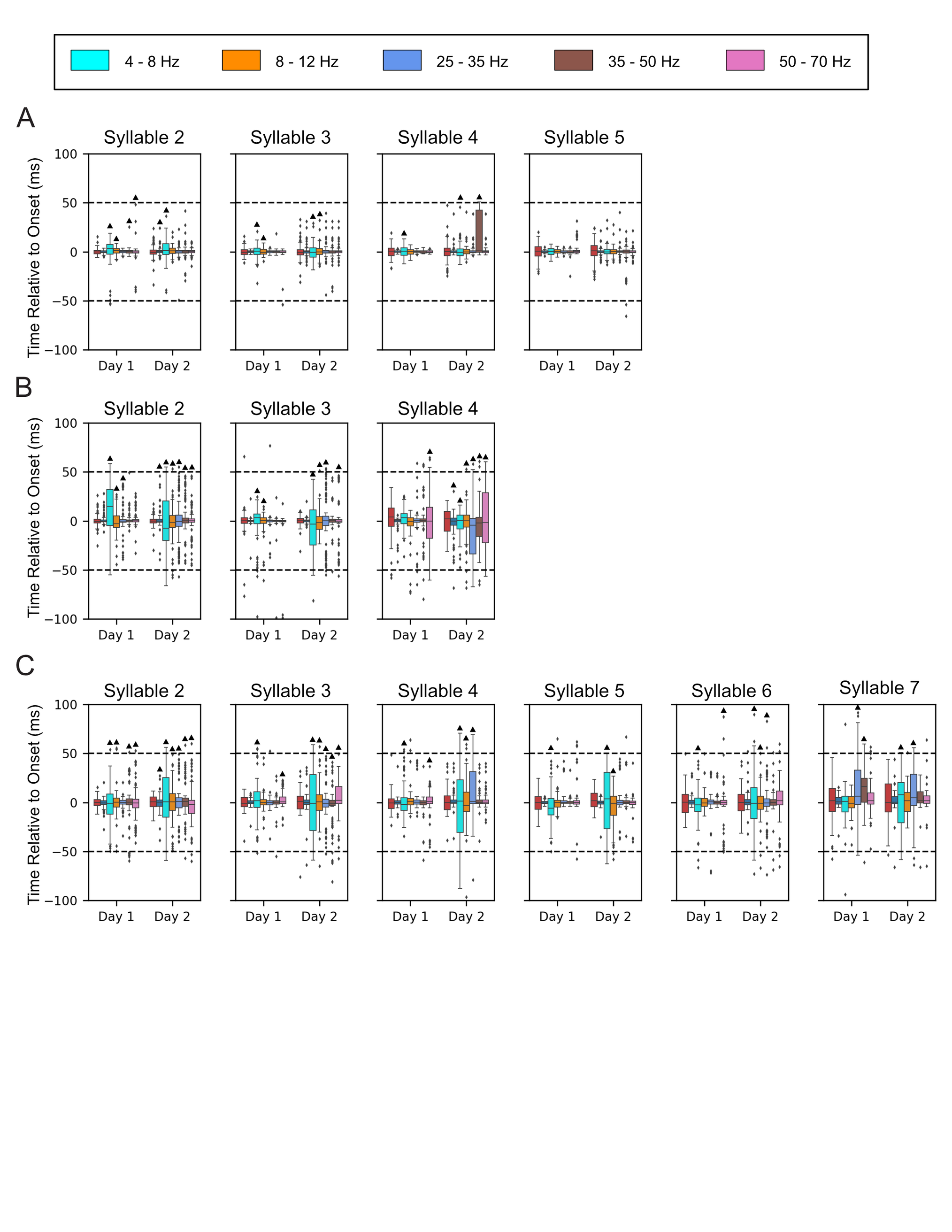

Supplement: S20 Fig — Boxplots of onset prediction times relative to the labeled onset time for each bird for every syllable for two highest-yielding days. Each column reflects the result for syllable number within the motif. Each row is for a specific bird with (A) corresponding to z007, (B) corresponding to z020, and (C) corresponding to z017. The order of each feature used is the same, going left to right: first is the stereotyped onset time using only the deterministic behavior, next is the results using all of the neural features, then each frequency band only in order from least to greatest (4–8 Hz, 8–12 Hz, 25–35 Hz, 35–50 Hz, and finally 50–70 Hz). The recording day designation number refers to the chronological order that the recordings took place. Statistical significance was calculated using the one-sided Wilcoxon signed-rank test, and ▲ denotes results that were not statistically significant when using the Benjamini-Hochberg False Discovery Rate. All other results p<0.05 and q<0.05. (TIF) [file pcbi.1008100.s031.tif]
